# Bicyclic guanidine superbase carboxylate salts for cellulose dissolution†

**DOI:** 10.1039/d4ra01734j

**Published:** 2024-04-16

**Authors:** Eva Gazagnaire, Jussi Helminen, Alistair W. T. King, Thomas Golin Almeida, Theo Kurten, Ilkka Kilpeläinen

**Affiliations:** a Department of Chemistry, Material Division, University of Helsinki FI-00560 Helsinki Finland ilkka.kilpelainen@helsinki.fi; b VTT Technical Research Centre of Finland Ltd Tietotie 4e 02150 Espoo Finland; c Institute for Atmospheric and Earth System Research/Chemistry, Faculty of Science FI-00560 Helsinki Finland

## Abstract

Bicyclic guanidines are utilized in organic synthesis as base catalysts or reagents. They also offer a platform for coordination chemistry, for example in CO_2_ activation, and their carboxylate salts offer an efficient media for cellulose dissolution. We have studied a series of bicyclic guanidines with varying ring sizes and with varying methyl substituents with a specific aim to find hydrolytically stable acetate salts for dissolution and processing of cellulose. Different superbase synthesis pathways were tested, followed by hydrolytic stability and cellulose dissolution capacity tests. The synthesis pathways were designed to enable the scale up of the production of the superbases considering the availability of the starting molecules and the feasibility of the synthesis. As a result, we found several hydrolytically stable bicyclic guanidine structures, which can overcome many of the reoccurring problems as carboxylate salts or free bases.

## Abstract 2

Bicyclic guanidines are utilized in organic synthesis as base catalysts or reagents.

## Graphical abstract

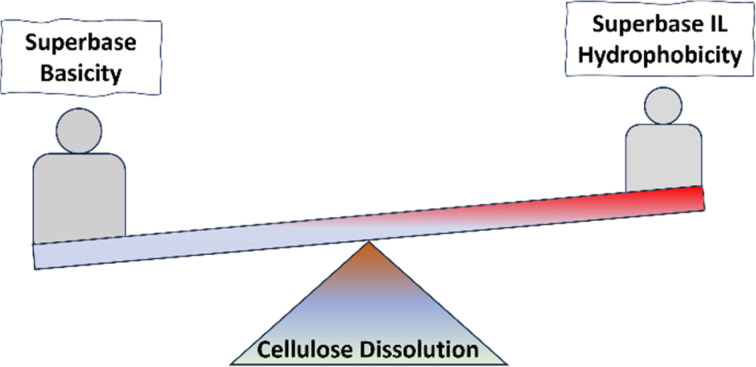

## Introduction

Strong Brønsted bases are widely used in organic chemistry due to the number of reactions requiring a deprotonation step.^1^ Organic bases offer some inherent advantages over the inorganic ones – they are usually soluble in organic reaction media enabling homogenous reaction conditions. Bicyclic guanidines and amidines belong to the family of organic superbases.^2,3^ Their exceptional basicity and their solubility in most of the organic solvents made them highly interesting as strong base catalysts and reagents.^2,4–6^ The rigid bicyclic structure enables strong resonance stabilization of charge throughout the molecule increasing their basicity, but also increases their overall chemical and physical stability.^3,7^ In general, guanidines are stronger bases than amidines due to stronger resonance stabilization provided by three nitrogen atoms.^3^ However, in bicyclic structures the size of the rings contributes strongly to the basicity.^3^ Changes the ring sizes may inhibit adaptation of a favourable conformation for the protonated form, which can jeopardize the effect from the possible resonance stabilization.^3^ From commercially available bicyclic amidines, 1,8-diazabicyclo[5.4.0]undec-7-ene (DBU) and 1,5-diazabicyclo[4.3.0]non-5-ene (DBN) are considered as non-nucleophilic superbases and they are highly efficient, for example, in dehydrohalogenation reactions.^3^ DBU appears to be the strongest of the common amidine bases with a basicity close to guanidines. However, DBN is somewhat less basic, as the second 5-membered ring is somewhat strained in the protonated form.^3,8^ Amidines are somewhat prone to hydrolysis by water producing aminolactams or aminoureas, which not only limits their applicability in aqueous media, but may also introduce difficulties in the work-up of the product.^7,9^

Guanidines are the strongest nitrogen-containing bases, which makes them very useful as basic catalysts in several organic reactions. From all the possible structures, 1,1,3,3-tetramethyl guanidine (TMG) is a very well-known, non-nucleophilic superbase. Despite its commercial availability and its wide applicability, TMG has limitations, like poor hydrolytic stability and instability under oxidative conditions.^10^

The commercially available bicyclic guanidines, 1,5,7-triazabicyclo[4.4.0]dec-5-ene (TBD) and its analogue 7-methyl-1,5,7-triazabicyclo[4.4.0]dec-5-ene (mTBD) offer improved chemical stability due to their bicyclic structures.^3,9^

The salts of organic superbases have raised attention due to their rather unique properties.^11–14^ The melting point of superbase carboxylate salts are usually low, allowing their use as solvents or reaction media.^15^ Our interest in these superbase ionic liquids (SBILs) arises from their efficiency for dissolution of cellulose.^16–19^ The dissolved cellulose can be regenerated for example in the form of textile fibres by addition of water as an antisolvent.^20–23^ Our work started with amidine salts, like [DBNH][OAc], which is an excellent solvent for cellulose, but suffers from poor hydrolytic stability.^24^ Therefore, the work continued towards mapping properties of bicyclic guanidines. It was soon found that [mTBDH][OAc] already has improved hydrolytic stability and also shows excellent dissolution capability towards cellulose and pulp.^25,26^ As the commercial price of these types of superbases can be rather high, we have also developed an efficient method for their synthesis.^27^

In the pathway from wood cellulose to man-made textile fibres, the SBIL encounters varying stages with different water contents and temperatures.^28,29^ Water is used as the antisolvent in the spinning bath to regenerate the cellulose fibres. The SBIL is then recovered for reuse by removal of water. During this step, elevated temperatures are required which may lead to hydrolysis of the SBIL.^29^ Even though [mTBDH][OAc] is rather resistant towards hydrolysis, it still undergoes slow degradation in successive recycling series.^24,25,29^ We undertook the challenge to find hydrolytically stable SBILs to be used for cellulose dissolution.

## Results and discussion

**Chart 1:**
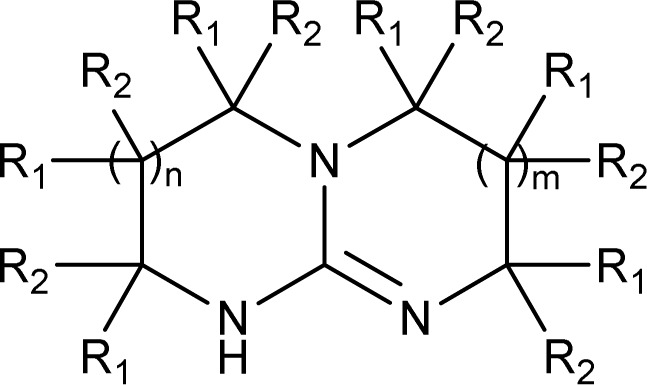
Generic structure of bicyclic guanidine superbases. R_1_ and R_2_ are hydrogen or alkyl (or any other substituent) independently. If the ring sizes are limited from 5 to 7 (*n*, *m* = 0–2, independently), and R_1_ & R_2_ to H and/or Me, the number of possible structures is well over 500.

In principle, an almost unlimited number of different bicyclic superbases can be designed by varying the ring sizes and introducing different substituents (Chart 1).

**Scheme 1:**
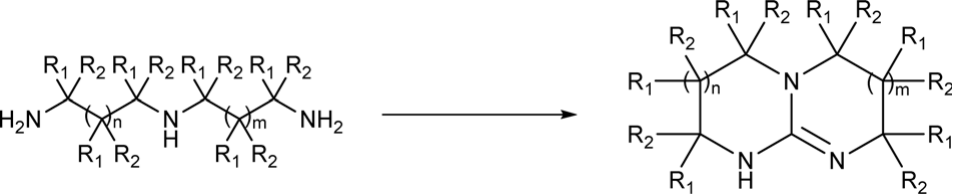
Bicyclic guanidines are directly available from suitable triamines (R_1_, R_2_ = H or Me and *n*, *m* = 0–2 independently). The structure of the starting triamine defines the structure of the resulting bicyclic guanidine.

A straightforward pathway to bicyclic guanidines is utilizing triamines as starting compounds (Scheme 1). The structure of the obtained superbase can be designed by varying the structure of the starting triamine, both in terms of ring sizes and substitution pattern. Depending on the individual case, the product is obtained as a mixture of isomers with different location of the double bond.

Only few possible triamines are available commercially: diethylene-, dipropylene- and ethylene propylene triamines are used as the amine component in epoxy glues and are thus readily available and relatively cheap.^30^ On the other hand, spermidine (*N*1-(3-aminopropyl)butane-1,4-diamine) is well known, but usually used only in small quantities in mostly biological research, making it expensive.^31^ A few other branched triamines are known, but available only in minor quantities. Therefore, we decided to explore possible pathways towards various triamines to allow for the facile synthesis of various bicyclic guanidines. As the structural space of the possible bicyclic guanidine superbase structures is large (Chart 1), we limited the size of the rings to 5–7, and the ring substituents to 1–4 methyl groups for practical reasons (Scheme 1).

**Scheme 2:**
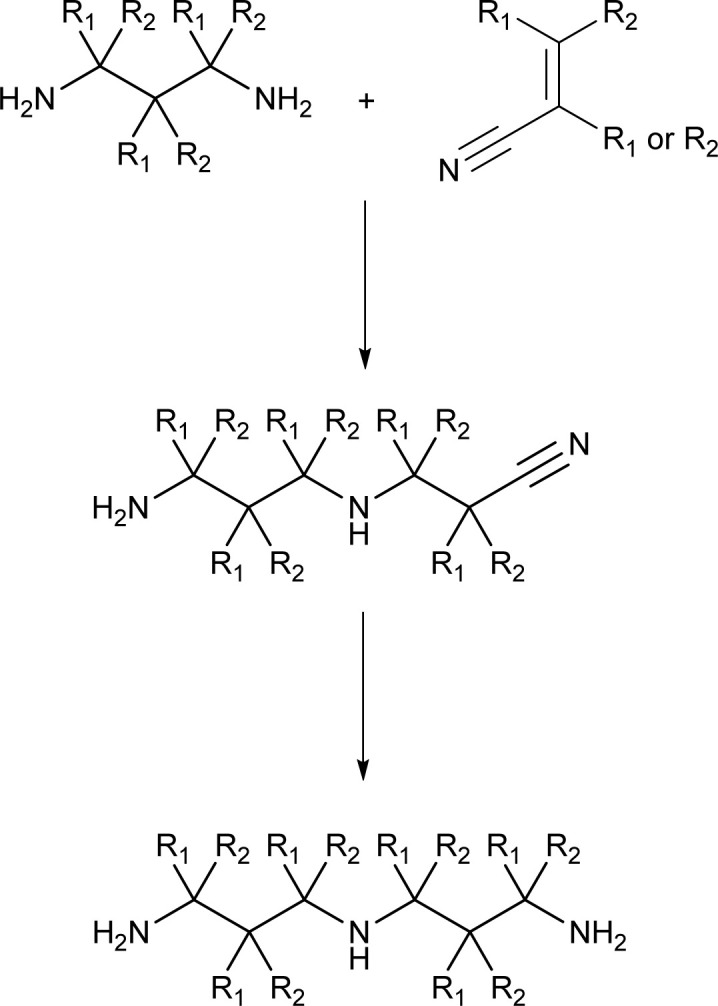
The targeted triamine can be synthesized from a suitable diamine and (substituted) conjugated nitrile by a Michael addition reaction followed by reduction step. (R_1_, R_2_ = H or Me independently).

Retrosynthetic analysis of the triamines suggests several possible pathways (see ESI†), but during the progress of the work, we utilized only two of them (Scheme 2). A facile approach was to utilize Michael reaction of a diamine with acrylonitrile, followed by reduction to the triamine (Scheme 2).^32,33^

**Chart 2:**
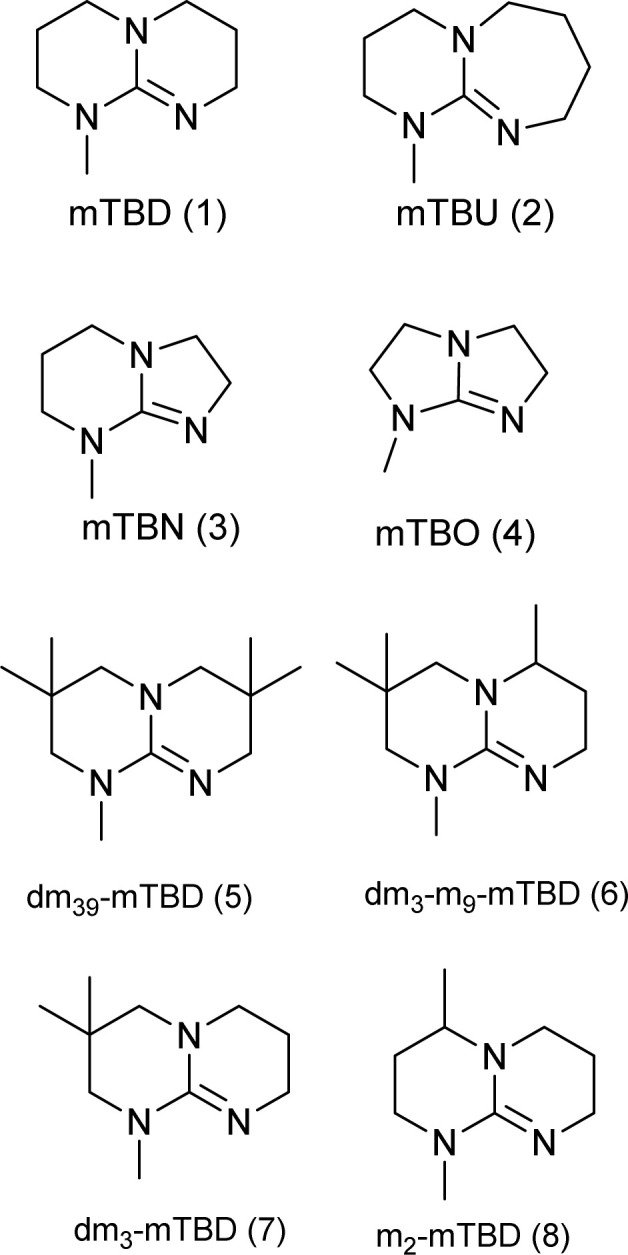
Structures of the final set of bicyclic guanidine superbases. mTBD (1) is commercially available and was utilized for comparison.

From the synthetically feasible structures, the targeted superbases were refined towards predicted hydrolytic stability with the help of molecular modelling (further discussion about hydrolytic stabilities below and full details are given in the ESI†). This allowed us to select the most promising structures for experimental studies. In addition, we also selected some synthetically attractive candidates (such as mTBO and m2-mTBD, Chart 2) to be used as a reference for the predicted hydrolytic stability.

We have earlier demonstrated that the predicted proton affinity of a superbase correlates well with the capability of the conjugate acetate salt to dissolve cellulose.^26^ Also now, we first utilized molecular modelling as a tool to us guide towards targeted cellulose dissolving SBIL structures. However, during the progress of the work, we noticed that while the calculated proton affinity indeed has a correlation with the capability for cellulose dissolution, the hydrophobicity of the superbase also seems to play a role, as the cellulose dissolution capability of the SBILs decreases with an increasing number of methyl substituents.

As a result of refining the targeted molecules both in terms of predicted hydrolytic stability and capability for dissolution of cellulose of the acetate salt, we ended up with a refined set of targeted structures (Chart 2 and ESI†).

### Triamine syntheses

In most cases, the targeted superbases (Chart 2) were synthesized reacting suitable diamines with acrylonitrile or crotonitrile, followed by catalytic hydrogenation (Scheme 2, see ESI† for detailed Experimental procedure).

Using an excess of the diamine will reduce the likelihood of obtaining the double reacted diamine in the first step (Michael addition) of the synthesis. The normal method to conduct these types of reactions is slow addition of the acrylo- or crotonitrile into a solution of the diamine to maximize the yield of the desired product. It was enough to utilize only a small excess of 1.1–1.5 of diamine yielding ≤20% of the bis-adduct indicating a rather good selectivity.

We also attempted controlling the reaction with temperature and addition rates, but all cases resulted in a similar composition, which indicates that the reaction is kinetically controlled by the reactivity of the diamine *vs.* the product reacting further yielding the bis-adduct. In all cases, the product was purified by direct vacuum distillation from the crude reaction mixture. As always, there are numerous possibilities for the synthesis of targeted structures. An alternative pathway towards triamines is using an (amino) alkyl bromide salt and a diamine as starting materials and is described in the ESI† (general procedure for nucleophilic attack Route B).

### Synthesis of the superbase(s)

Bicyclic guanidines are usually synthesized using a suitable triamine as the starting structure, which is reacted with guanidine hydrochloride or dicyandiamide (DCD) to provide the targeted superbase.^34,35^

At the beginning of this work, we decided to focus on the guanidine route for known performance.^35^ However, from this route, the guanidine superbases are obtained in a salt form and need to be released in a similar way than for the triamine synthesis described earlier, complicating the work-up of the product prior to the methylation step. Similar to the guanidine route, also the reaction of the triamine with DCD is classically conducted under acidic conditions yielding the formed superbase in a salt form.^34^

We have already earlier demonstrated that the DCD pathway also offers the possibility to obtain the superbase directly, *i.e.* avoiding the costly work-up from the salt form (and ready for methylation).^27^ This alternative is particularly attractive for large scale synthesis but utilizes the triamine in excess to minimize formation of side products. However, the excess of the triamine can be easily recovered and re-used.^27^

From our data, it is difficult to find guidelines which cyclization method should be used for the synthesis of a given superbase and comparison between these two approaches requires detailed analysis of the reaction pathways (mechanisms), which is beyond the scope of the current work.^36^ The utilized DCD pathway worked well for synthesis of structures 1, 3, 7 and 8, and is clearly the method of choice for larger scale production (scaling up structures 5 and 6 was not attempted). However, for production of 2, the guanidine pathway was efficient, but the DCD approach failed.

For the final *N*-methylation step, we used dimethyl carbonate, which yielded the targeted structures essentially quantitatively, except for structure 2, where the yield was only modest (∼20%). However, no attempts were made using other methylation pathways for 2, as the corresponding SBIL was not particularly attractive for cellulose dissolution (see below).

Notably, when the bicyclic core of the superbase is not symmetrical (location of methyl branches or difference in rings sizes), the *N*-methylation results in a mixture of two (or more) isomers. However, having the superbase as a mixture of isomers actually lowers the melting point of the corresponding acetate salt (Table 1) which is beneficial for the targeted use in dissolution of cellulose. Therefore, no attempt was made to purify the isomers as pure compounds.

**Table 1:** Comparison of the hydrolytic stabilities of the acetate salts of the superbases. The acetate salts of 2,3,6,7 and 8 are mixtures due to the location of the double bond. In addition, 6,8 are mixtures of stereoisomers (see Tables S1 and S2)^a^

	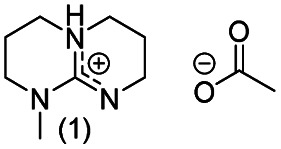	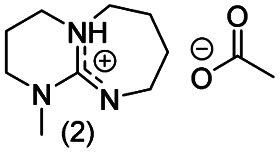	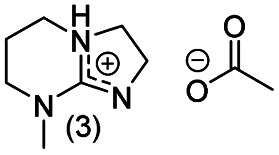	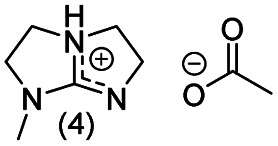	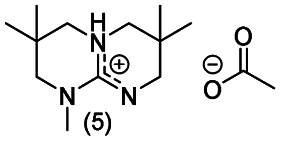	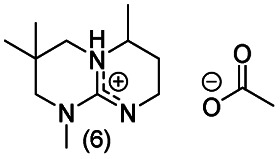	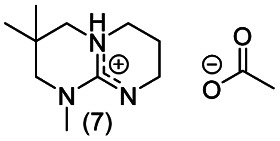	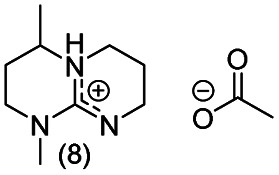
Hyd (mol%) at 90 °C	0	0	0	21	0	0	0	0
Hyd (mol%) at 130 °C	10	40	8	76	0	3	2	2
m.p	83	<RT	<RT	<RT	60	<RT	<RT	<RT
Cellulose dissolution (10 w/w%)	+	+	+	−	−	+	+	+
Viscosity of cellulose solution	**	***	*			***	*	*

a *The cellulose solution is elastic (‘stretchable’) at room temperature. **The cellulose solution is more viscous/elastic but can still be stretched at RT. ***The cellulose solution is too viscous to be stretched at RT (solid gel like). +The cellulose is completely dissolved as followed by optical microscope. −Some cellulose remains undissolved. For detailed experimental conditions, see ESI. Also, the relative hydrolytic stabilities of the free bases are provided in the ESI (Table S3).

### Hydrolytic stability of the SB-ILs

A reoccurring problem of guanidine superbases is their inherent hydrolytic instability.^24,25,29^ While this issue is well recognized, there is only a limited amount of published work on the systematic screening alternative structures that could circumvent this re-occurring issue. Even though it is well known that the salt forms of superbases are generally more stable than the superbase itself,^37^ the bicyclic guanidine acetate salts used in cellulose dissolution undergo slow hydrolysis. This may jeopardize the whole process, as the SBIL undergoes repeated cycles of water additions and removals in a circulating process.^29^

To assess the relative hydrolytic stabilities of the synthesized structures, we set up a straightforward and robust approach to test the stability between series superbase acetate salts (Table 1, see Table S3† for free superbases) in water. While it is recognized that actual hydrolysis speeds are also dependent on the amount of water, we chose a constant ratio of 1 : 1 (mol mol^−1^), where the hydrolysis speed is easy to follow and allows to directly compare the relative stabilities.

As can be seen in Table 1, in general all the methyl substituted bicyclic guanidine ILs are more stable than the commercially available [mTBDH][OAc]. Already a slightly increasing hydrophobicity seems to increase stability in water, as even one methyl substituent is enough to reduce hydrolysis, while when the number exceeds ∼3, the SBIL becomes stable even at elevated temperatures. However, the stability of a given structure is not only dependent on the number of methyl substituents, but also on the position of the methyl(s) (*cf.* [dm3-m0-mTBDH] and [m2-mTBDH][OAc]). Overall, both the number of methyl substituents and the substitution pattern contribute to the stability of these SBIL.

A clear exception in hydrolytic behaviour is seen for mTBO. While the free base is relatively stable (see Table S3†), the acetate salt is highly prone to hydrolysis. Even though mTBO is a guanidine, its 5–5 bicyclic structure inhibits the adoption of a planar geometry for the protonated structure making it less basic.^3^ In addition to cellulose dissolution with SBILs, the free superbases find use as strongly basic catalysts and reagents. Also in these applications, the rather poor hydrolytic stability is a reoccurring problem. Many of the current superbases show considerably improved hydrolytic stability, as shown in the ESI,† but their comparison is out from the scope of the current work.

As discussed earlier, we used molecular modelling as a qualitative tool to guide the selection of targeted bicyclic guanidine structures. The selected method for the modelling is relatively simple and robust (COSMO-RS, see ESI†), but seems to provide sufficient accuracy for estimating relative hydrolytic stabilities.

The predicted hydrolysis free-energy (ΔhG) values from molecular modelling show a qualitative agreement with ΔhG values estimated from experimental conversion percentages (Fig. S48† for details). Closer agreement with experimental data was perhaps not achieved due to a number of factors: (1) experimental conversion percentages may include a contribution from the two different hydrolysis products, whose ratios are not known (except for mTBD) due to their convoluted NMR signals. (2) Possible influence of kinetic control. (3) Uncertainties associated with the computational methods themselves. Nevertheless, the computational methods employed in this work captured the general trend in the hydrolytic stability of SB-ILs.

### Capability of SB-ILs to dissolve cellulose

The capability of the acetate salts of different structures (Chart 1 and Table 1) to dissolve cellulose was assessed using hardwood (birch) kraft pulp as substrate, as it is highly demanding for the dissolution efficiency, but is also an industrially relevant source for cellulose. The dissolution tests were first conducted using 10 w/w% pulp in dry SB-IL with mechanical stirring at 85 °C, 3 hours. The dissolution of the samples was followed with an optical microscope utilizing cross-polarizing filters, to highlight birefringence from residual undissolved cellulose, and confirmed with ^1^H NMR. The results from the dissolution trials are collected in Tables 1 and 2.

**Table 2:** Microscope pictures of cellulose dissolved in SBILs. The round objects are air bubbles. For [dm30-mTBDH][OAc] and [mTBOH][OAc], undissolved cellulose fibers are remaining visible under these dissolution conditions (10 w/w% pulp, mechanical stirring at 85 °C for 3 hours). An example of ^1^H NMR and diffusion edited ^1^H NMR of birch hard wood kraft pulp dissolved in SBILs can be found in the ESI

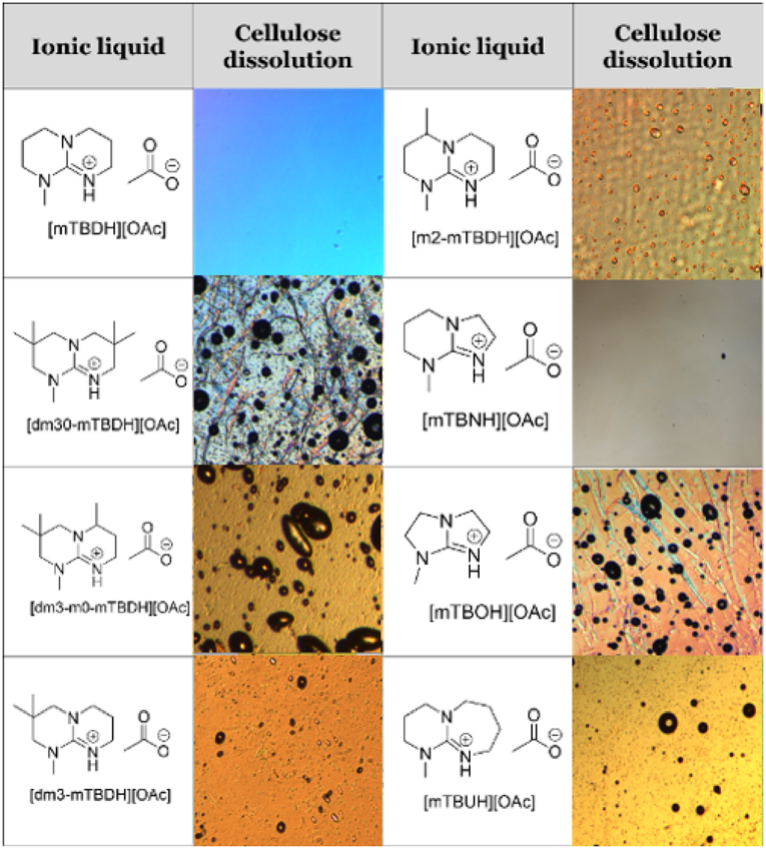

Most of the novel SBILs could dissolve cellulose well. We have earlier postulated that the ability for cellulose dissolution correlates with increasing basicity of the base in protic ionic liquids, which consistent with the poor dissolution capability of [mTBOH][OAc].^26^ However, increasing amount of methyl substituents increases the hydrophobicity of the superbase, which seems eventually limit the dissolution capability, as is apparent for [dm3-m0-mTBDH][OAc] and [dm30-mTBDH][OAc]. This can be attributed to the hydrophilic character of cellulose. This ‘immiscibility’ of the solvent and substrate then leads to loss of dissolution capability. Supporting this hypothesis, addition of a small amount (10 wt% from SB-IL) of DMSO as an amphiphilic co-solvent already allowed for complete dissolution of the pulp with both of these more hydrophobic SBILs.

In terms of reusability, [mTBNH][OAc] is a good example as it can be used repeatedly to dissolve cellulose without significant degradation of the SB-IL or cellulose.^15^

^1^H and diffusion edited ^1^H NMR spectra of cellulose dissolved in SB-ILs are typical for polymeric cellulose dissolved in SB-ILs (Fig. S43 and S44,† [m2-mTBDH][OAc] is taken as an example) and show no chemical changes taking place during dissolution. Three months later, the same sample was run again resulting identical spectra, *i.e.*, no degradation of cellulose took place even at prolonged storage.

**Fig. 1:**
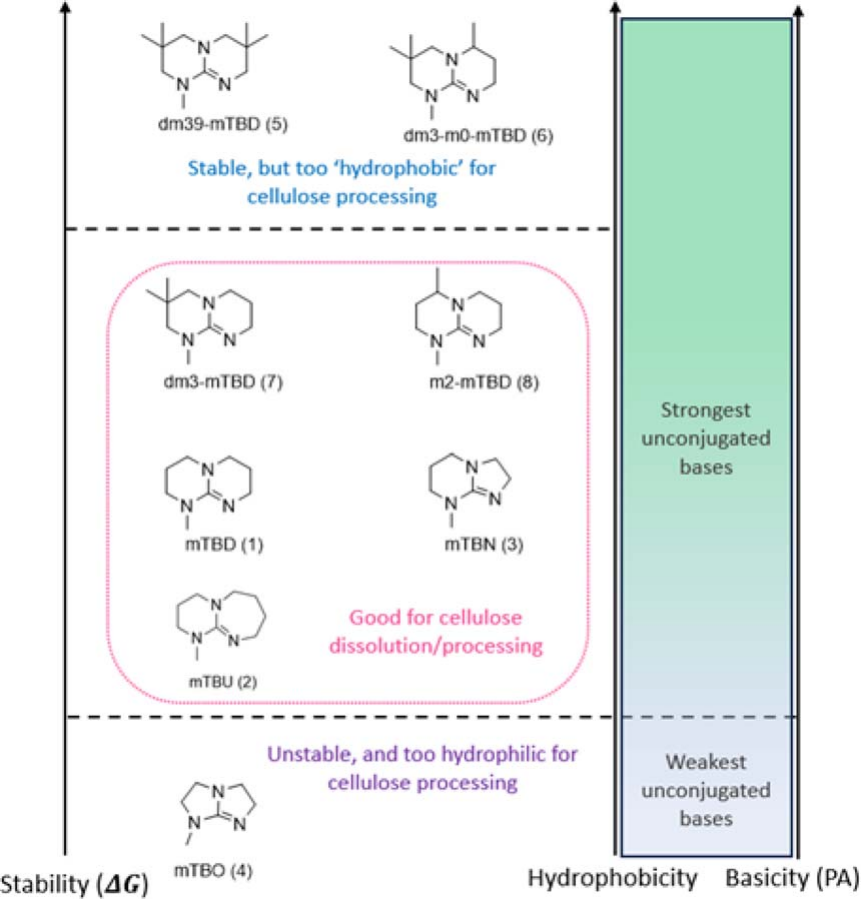
Ranking of the SBILs structure by order of hydrophobicity (for simplification, only the structure of the superbase of the SBIL is shown).

To summarize, for cellulose dissolution, a rather hydrophilic SB-IL is beneficial, but the more hydrophobic structures show better hydrolytic stabilities. These properties are contradictory, but balancing between these is possible and there is a ‘window’, where the SB-IL shows good hydrolytic stability while maintaining good capacity for dissolution of cellulose (Fig. 1).

When using SBILs for spinning of textile fibres, the requirements for the solution physical properties are essential. While the regeneration/spinning for fibres is out of the scope of the current work, some general comments can be made. Most of the SBILs in the ‘cellulose dissolution window’ are likely to perform well in spinning processes. Their capacity for cellulose dissolution is high enough, and their viscosity is close to the reference, [mTBDH][OAc], or less, thus enabling operation at lower spinning temperatures.

## Conclusions

In summary, we have addressed the water stability of bicyclic guanidine superbases (*c.f.* ESI†) and their acetate salts. By varying methyl substituents and ring sizes, several superbases with good tolerance to water were identified.

The selection of targeted structures was conducted with the help of molecular modelling to predict water stability and cellulose dissolution capacity. The final set of structures was selected based on synthesis feasibility. The synthesis pathways were selected with aim to enable scaling up the production to larger scales, from the viewpoint of starting compounds, reaction conditions and easiness of workup of the final product.

In the current work, we have demonstrated small modifications in structure is enough to stabilize bicyclic guanidines against hydrolysis (*c.f.* ESI†). It is likely that stable amidines can be prepared with similar approach. In most cases, the acetate salts of these new guanidine structures showed very good dissolution capacity for cellulose making them attractive for large scale applications, *e.g.*, processes for man-made cellulose fibres. The improved hydrolysis stability of the developed new superbases can overcome many of the reoccurring problems as catalysts or reagents in more general organic reactions.

## Author contributions

Eva Gazagnaire: investigation, methodology, validation, writing – original daft. Jussi Helminen: writing – review & editing. Alistair King: formal analysis, writing – review & editing. Thomas Golin Almeida and Theo Kurten: formal analysis, writing – review & editing. Ilkka Kilpeläinen: conceptualization, investigation, supervision, resources, writing – review & editing, funding acquisition.

## Conflicts of interest

There are no conflicts to declare.

## Supplementary Material

RA-014-D4RA01734J-s001
