# Supplementary material for: Bicyclic guanidine superbase carboxylate salts for cellulose dissolution
Source: RSC Adv. 2024 Apr 16;14(17):12119–24. doi: 10.1039/d4ra01734j (PMC11019349; doi:10.1039/d4ra01734j)
Supplement: RA-014-D4RA01734J-s001 [file RA-014-D4RA01734J-s001.pdf]

Electronic Supplementary Material (ESI) for RSC Advances.
This journal is © The Royal Society of Chemistry 2024

Supporting information

Bicyclic Guanidine Superbase Carboxylate Salts for Cellulose Dissolution

Eva Gazagnaire, Jussi Helminen, Alistair W. T. King, Thomas Golin Almeida, Theo Kurten and Ilkka Kilpeläinen

[a] Materials Chemistry Division, Department of Chemistry, University of Helsinki, FI-00560 Helsinki, Finland.

[b] Institute for Atmospheric and Earth System Research/Chemistry, Faculty of Science, FI-00560 Helsinki, Finland.

[c] VTT Technical Research Centre of Finland Ltd, Tietotie 4e, 02150 Espoo, Finland.

Table of content

I. Structures **5**

II. Possible pathways to prepare the novel triamines **7**

III. Synthesis of Superbases and their carboxylate salts. **7**

1. Synthesis of 7-methyl-1,5,7-Triazabicyclo[4.4.0]dec-5-enium Acetate (1). 9
   - *a. Cyclisation of 1,3-Propanediamine (a):* *9*
   - *b. Methylation of 1,5,7-Triazabicyclo[4.4.0]dec-5-ene.* *10*
   - *c. Synthesis of 7-methyl-1,5,7-Triazabicyclo[4.4.0]dec-5-enium Acetate.* *10*
   - *d. Hydrolysis of 7-methyl-1,5,7-Triazabicyclo[4.4.0]dec-5-enium Acetate.* *10*
2. Synthesis of 5-methyl-1,5,7-triazabicyclo[4.5.0]undec-6-enium Acetate (2). 10
   - *e. Cyclisation of N1-(3-aminopropyl)butane-1,4-diamine (c)* *10*
   - *f. Methylation of 1,5,7-triazabicyclo[4.5.0]undec-5-ene.* *10*
   - *g. Synthesis of 5-methyl-1,5,7-triazabicyclo[4.5.0]undec-6-enium Acetate.* *10*
   - *h. Hydrolysis of 5-methyl-1,5,7-triazabicyclo[4.5.0]undec-6-enium Acetate.* *11*
3. Synthesis of 5-methyl-1,5,7-triazabicyclo[4.3.0]non-6-enium Acetate (3). 11
   - *i. Cyclisation of N1-(2-aminoethyl)propane-1,3-diamine (b).* *11*
   - *j. Methylation of 1,5,7-triazabicyclo[4.3.0]non-5-ene.* *11*
   - *k. Synthesis of 5-methyl-1,5,7-triazabicyclo[4.3.0]non-6-enium Acetate.* *11*
   - *l. Hydrolysis of 5-methyl-1,5,7-triazabicyclo[4.3.0]non-6-enium Acetate.* *11*
4. Synthesis of 4-methyl-1,4,6-triazabicyclo[3.3.0]oct-5-ene Acetate (4): 11
   - *m. Cyclisation of N1-(2-aminoethyl)ethane-1,2-diamine (d)* *11*
   - *n. Methylation of 1,4,6-triazabicyclo[3.3.0]oct-4-ene.* *12*
   - *o. Synthesis of 4-methyl-1,4,6-triazabicyclo[3.3.0]oct-5-enium Acetate.* *12*
   - *p. Hydrolysis of 4-methyl-1,4,6-triazabicyclo[3.3.0]oct-5-enium Acetate* *12*
5. Synthesis of 7-methyl-3,3-dimethyl-9,9-dimethyl-1,5,7-Triazabicyclo[4.4.0]dec-5-enium Acetate (5). 12
   - *q. Synthesis of 3,3’-Iminobis[2,2-dimethylpropanal] 1,1’-dioxime.* *12*
   - *r. Synthesis of N1-(3-amino-2,2-dimethylpropyl)-2,2-dimethylpropane-1,3-diamine.* *12*
   - *s. Cyclisation of N1-(3-amino-2,2-dimethylpropyl)-2,2-dimethylpropane-1,3-diamine.* *13*
   - *t. Methylation of 3,3-dimethyl-9,9-dimethyl-1,5,7-Triazabicyclo[4.4.0]dec-5-ene.* *13*

u. Synthesis of 7-methyl-3,3-dimethyl-9,9-dimethyl-1,5,7-Triazabicyclo[4.4.0]dec-5-enium Acetate. *13*

v. Hydrolysis of 7-methyl-3,3-dimethyl-9,9-dimethyl-1,5,7-Triazabicyclo[4.4.0]dec-5-enium Acetate. *13*

6. Synthesis of 7-methyl-3,3-dimethyl-10-methyl-1,5,7-Triazabicyclo[4.4.0]dec-5-enium Acetate (6). 14

w. Synthesis of 3-((3-amino-2,2-dimethylpropyl)amino)butanenitrile. *14*

x. Synthesis of N3-(3-amino-2,2-dimethylpropyl)butane-1,3-diamine. *14*

y. Cyclisation of N3-(3-amino-2,2-dimethylpropyl)butane-1,3-diamine. *14*

z. Methylation of 3,3-dimethyl-10-methyl-1,5,7-Triazabicyclo[4.4.0]dec-5-ene. *14*

aa. Synthesis of 7-methyl-3,3-dimethyl-10-methyl-1,5,7-Triazabicyclo[4.4.0]dec-5-enium Acetate. 14

bb. Hydrolysis of 7-methyl-3,3-dimethyl-10-methyl-1,5,7-Triazabicyclo[4.4.0]dec-5-enium Acetate. 15

7. Synthesis of 7-methyl-3,3-dimethyl-1,5,7-Triazabicyclo[4.4.0]dec-5-enium Acetate (7). 15

cc. Synthesis of 3-((3-amino-2,2-dimethylpropyl)amino)propanenitrile. *15*

dd. Synthesis of N1-(3-aminopropyl)-2,2-dimethylpropane-1,3-diamine. *15*

ee. Cyclisation of N1-(3-aminopropyl)-2,2-dimethylpropane-1,3-diamine. *15*

ff. Methylation of 3,3-dimethyl-1,5,7-Triazabicyclo[4.4.0]dec-5-ene. *16*

gg. Synthesis of 7-methyl-3,3-dimethyl-1,5,7-Triazabicyclo[4.4.0]dec-5-enium Acetate. *16*

hh. Hydrolysis of 7-methyl-3,3-dimethyl-1,5,7-Triazabicyclo[4.4.0]dec-5-enium Acetate. *16*

8. Synthesis of 7-methyl-2-methyl-1,5,7-Triazabicyclo[4.4.0]dec-5-enium Acetate. 16

ii. Synthesis of 3-((3-aminopropyl)amino)butanenitrile. *16*

jj. Synthesis of N3-(3-aminopropyl)butane-1,3-diamine. *16*

kk. Cyclisation of N3-(3-aminopropyl)butane-1,3-diamine. *16*

ll. Methylation of 2-methyl-1,5,7-Triazabicyclo[4.4.0]dec-5-ene. *17*

mm. Synthesis of 7-methyl-2-methyl-1,5,7-Triazabicyclo[4.4.0]dec-5-enium Acetate. *17*

nn. Hydrolysis of 7-methyl-2-methyl-1,5,7-Triazabicyclo[4.4.0]dec-5-enium Acetate. *17*

IV. Calculation of hydrolysis for SB and SBILs from ^{1}H NMR **17**

V. 1D NMR spectra **18**

VI. Microscope images **35**

VII. Prediction of hydrolysis free energies with quantum chemistry **38**

a. Computational methods 38

b. Investigated systems 39

c. Free energies of hydrolysis 41

d. Interpretation of computational results 42

e. Comparison to hydrolysis experiments 43

References...44

I. Structures.

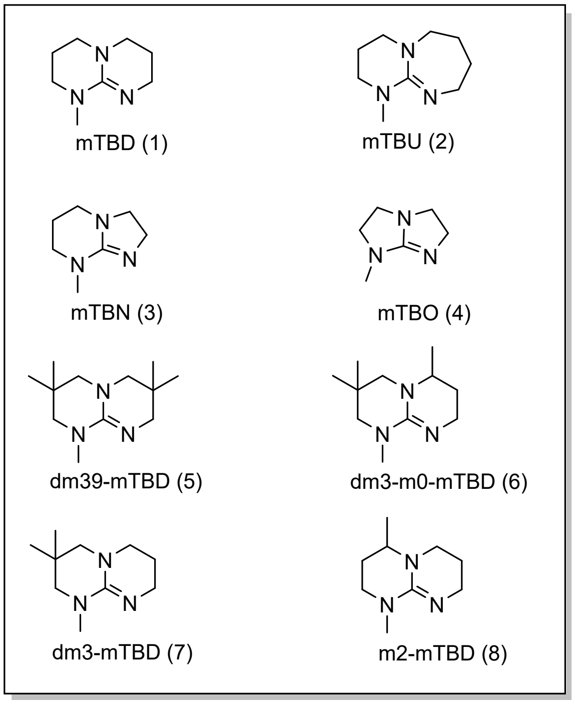

***Figure S1*:** structures of superbases used in the presen work.

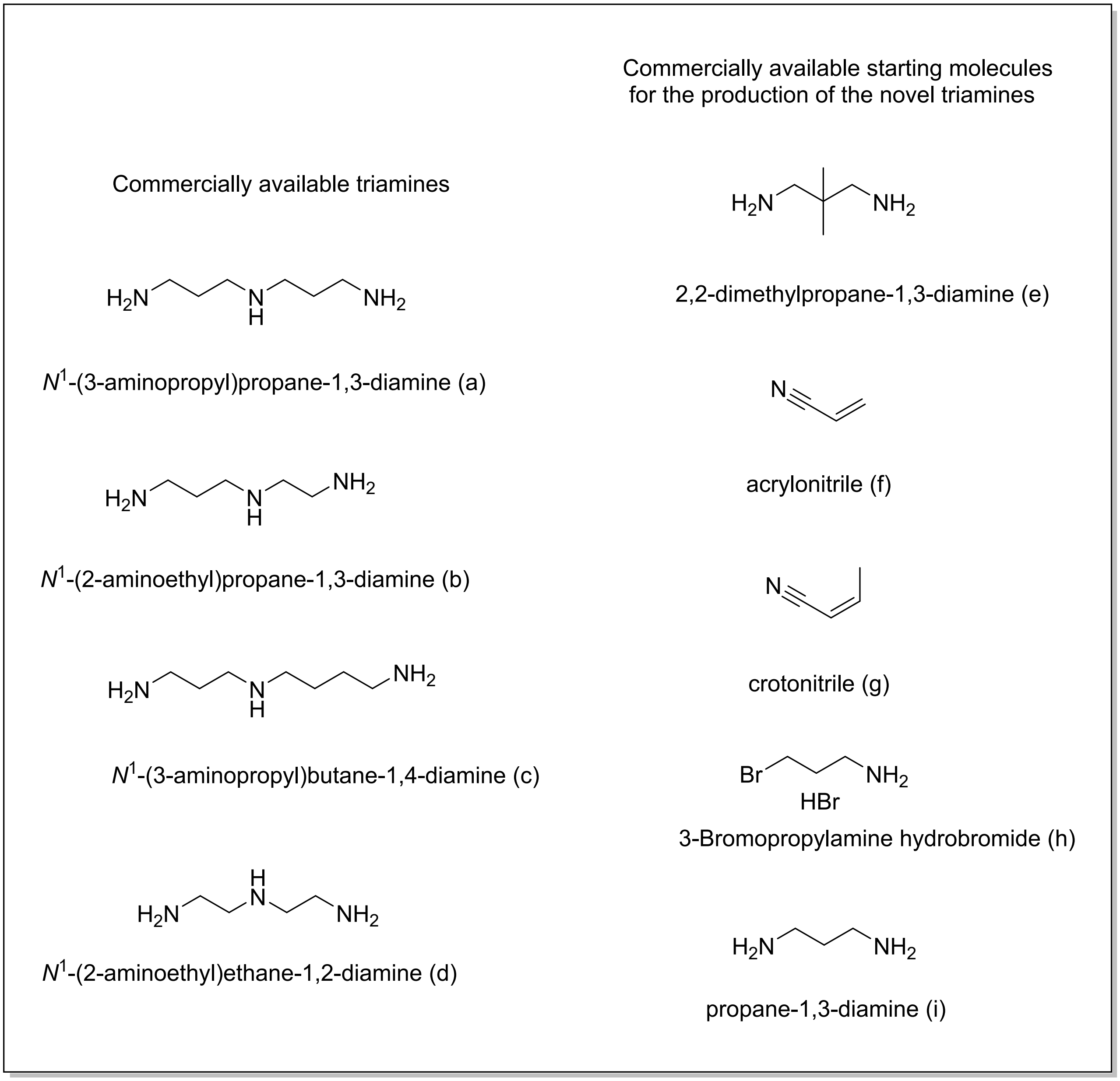

Figure S2: starting molecules used to prepare the novel triamines and superbases.

II. Possible pathways to prepare the novel triamines.

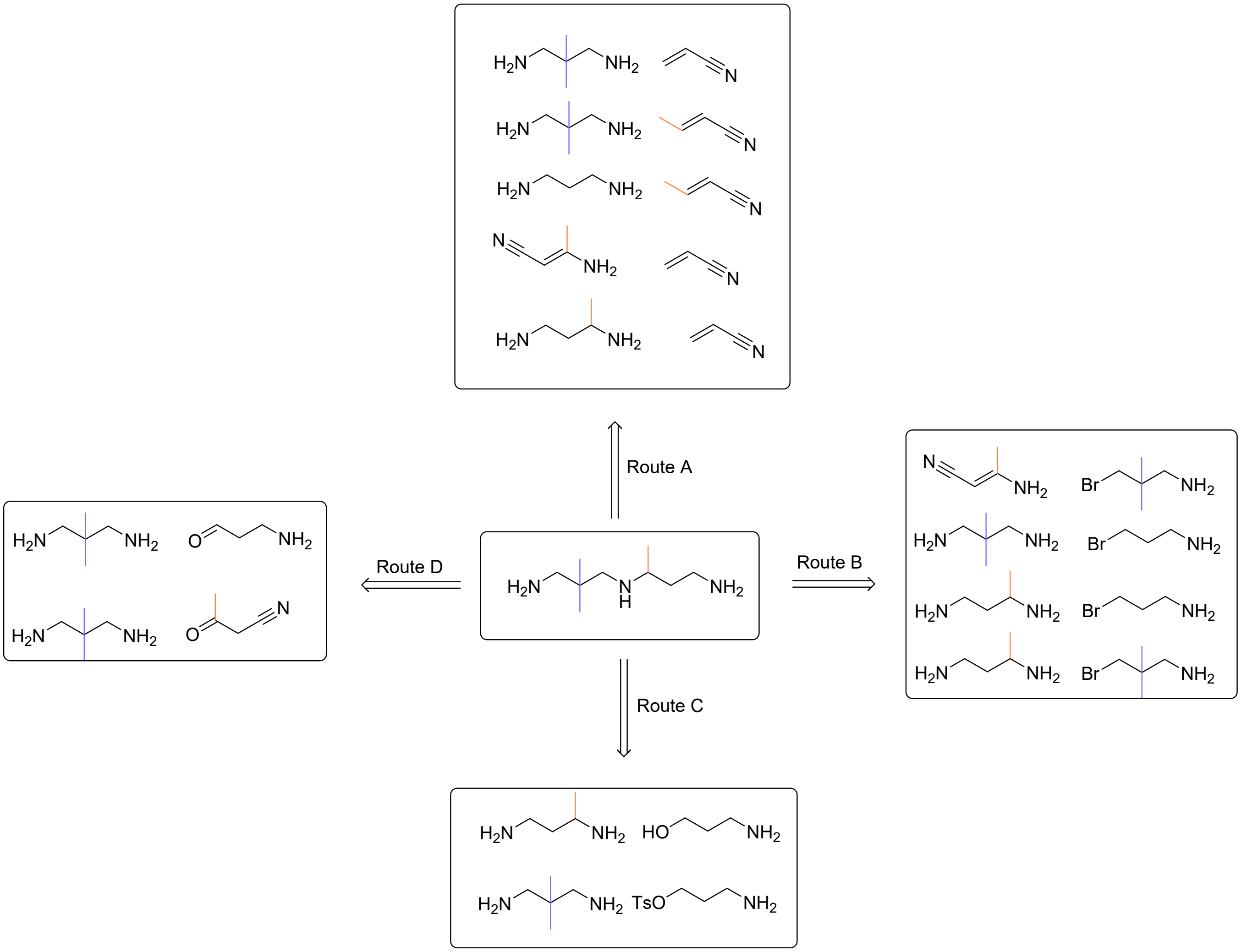

Figure S3**:** Scheme of the possible routes for synthesis of the novel triamines. In this work, only Route A and Route B were used.

As said in the main manuscript, the classical S_N2 reaction of suitable diamines with (amino)alkyl bromides. Clearly, this approach may suffer from (internal) reactions, but depending on the substitution pattern of both the diamine and (amino)alkyl bromide, the yields were still satisfactory (se general procedure for nucleophilic attack).

III. Synthesis of Superbases and their carboxylate salts.

General procedure for Michael addition reaction (Route A) (A): In a 250 mL round bottom flask (RBF) equipped with a reflux condenser and 6 equivalents of diamine dissolved in 100 mL of ethanol, 1 molar equivalent of conjugated nitrile compound was added dropwise (N.B! exothermic reaction). Then the rection mixture was refluxed for 18 hours. The day after the ethanol phase was removed with a rotavapor

and the excess of diamine distilled off under high vacuum. The triamine was used without further purification.

Warning: for the reaction through the Michael addition requiring the use of acrylonitrile, pay attention to its toxicity and its low boiling point.

General procedure for nucleophilic attack (Route B) (B):

As said in the main manuscript, the classical S_N2 reaction of suitable diamines with (amino)alkyl bromides. In this work, we only utilized alkyl halides as staring material for Route B, but using other leaving groups (potentially avoiding stable salt formation) are a feasible option. Clearly, this approach may suffer from (internal) reactions, but depending on the substitution pattern of both the diamine and (amino)alkyl bromide, the yields were still satisfactory (se general procedure for nucleophilic attack). The alternative route was to react diamine with an (amino)alkyl bromide salt though a S_N2 mechanism (Figure S3). In practice, an excess of the diamine throughout the reaction was necessary to avoid possible side reactions and to speed-up the conversion. From the reaction of diamine with alkyl halide, the triamine is obtained in a halide salt form, which was released using an excess of strong inorganic base, and finally distilled. Because this route requires an excess of base to free the triamine from the salt form, this be problematic at a large scale. Further, because of the large amount of solid present in the reaction mixture, the distillation of the final product becomes a challenge.

In a three neck 100 mL RBF, 4 molar equivalents of diamine were dissolved in isopropanol and heated to reflux (around 1g of reagent per 10 ml of solvent, can be diluted further). In a beaker, 1 molar equivalent of aminobromide salt was dissolved in isopropanol and added dropwise with a syringe pump to the flask. After the complete addition, the reaction was refluxed for 3 hours. To this mixture, 10 molar equivalents of KOH were added portion-wise and the mixture was refluxed for an extra hour. Once the mixture was cooled to room temperature, the white solid (excess of KOH and KBr) is filtered through a Buchner fritted filter (porosity 4) and the isopropanol dried with a rotavapor. The triamine needs to be distilled before the next synthesis step.

Warning: the addition of the aminobromide and the KOH quenching are exothermic reactions, it is recommended to adapt the speed of the addition to the temperature evolution.

General procedure for reduction by hydrogenation (C)**:** In a Parr reactor (450 mL volume capacity), 1 molar equivalent of triamine precursor was dissolved in a mixture of ethanol: water (2:1 v/v) containing 1.1 molar equivalent of sodium hydroxide. 10 wt% (according to triamine precursor) of Raney nickel was used as a catalyst. Once the reactor was sealed, it was pressurised with 50 bars of hydrogen and heated to 50 °C. The mixture was stirred for 20 hours. The day after, the catalyst was filtered off through a fritted filter (porosity 4) and the ethanol: water phase evaporated with a rotavapor. The solid-liquid mixture was distilled under high vacuum and gave a colourless liquid as a distillate. The solid inorganic base remained undistilled.

Warning:

- Before the reaction: Once the Parr reactor loaded and closed, pressure it at least two times to 20 bars of Nitrogen then a third time with the pressure needed for the reaction (here 50 bars). Once done, pressure the reactor to the pressure needed for the reaction with Hydrogen (here 50 bars), then release it and refill it for the reaction. In addition, do not pressure the reactor over half of its

pressure capacity as during the heating of the reactor the pressure increases of few bars (due to hydrogen and solvent). Once the reaction is ongoing the pressure decreases, an increase of 5-10 degrees can be observed so it is advised to heat up the reactor slowly (here for 50°C we took 30 min to reach the reaction temperature).

- After the reaction: let the Parr reactor cool to room temperature and slowly purge the hydrogen. Before opening the reactor, repeat the same steps as before the reaction: purge with nitrogen three times.
- During the filtration of the catalyst: it is advised to filter the catalyst over wet celite (use water) and to pay attention to have the Raney nickel always under the surface of the liquid as this is a highly pyrophoric compound. For higher safety, the filter can be rinsed using water instead of ethanol.

General procedure for cyclisation by guanidine route (D)**:** In a 100 mL round-bottom flask, 1 molar equivalent of triamine was mixed with 1 molar equivalent of guanidine chloride. The neat mixture was stirred for 20 hours at 150 °C. The next day the solid was dissolved in methanol and 2 molar equivalents of sodium hydroxide were added. After one-hour, extra methanol was added to decrease the viscosity of the mixture and the white solid (excess of NaOH and NaCl) were filtered using a Buchner fritted filter (porosity 4). The methanol is then removed with a rotavapor to give a white to pale yellow solid. The product can be used without further purification for the methylation step.

Warning: Ammonia is released during the reaction, it is advised to connect a tube from the flask to a container containing cold water. Careful during the reaction cooling as it can suck out the water to the reaction flask (vacuum induced)

General procedure for cyclisation of triamine by DCD route (E)**:** In a 1 L three neck flask equipped with a syringe pump and a reflux condenser, 4 molar equivalents of triamine and 0.003 molar equivalent of *p*-toluenesulphonic acid monohydrate were charged. The mixture was heated up to 200 °C. To this, a solution of 1 molar equivalent of dicyandiamine mixed with 2.5 molar equivalents of triamine was added over a period of three hours. Once the addition was done, the mixture was heated up to 220 °C for 30 minutes. After this, the reaction was cooled to 130 °C and the excess of triamine was distilled out and the product obtained as a white solid. The product was not further purified before the next step.

Warning: same recommendations as for the guanidine route.

General procedure for methylation of the superbase (F): In a 3-neck round bottom flask equipped with a reflux condenser, 1 molar equivalent of unmethylated superbase was dissolved in xylene and 1.3 molar equivalent of dimethyl carbonate were added dropwise with a syringe pump over a period of 3 hours, then the reaction mixture was refluxed overnight. The day after, the solvent was dried and the crude mixture was distilled under high vacuum to give a colourless liquid.

General procedure for synthesis of the superbase acetate salt (G): In a vial, 1 equivalent of superbase was mixed with 1 molar equivalent of acetic acid and were stirred at room temperature for 15 min (N.B! exothermic reaction). The product was obtained as a pale yellow to orange viscous liquid. In the case of [dm39-mTBDH][OAc], the SBIL is a white solid at room temperature.

General procedure for hydrolysis for the superbase (H): In a vial, 1 molar equivalent of SB was mixed with 1 molar equivalent of water. Then the vial was heated to 90 °C (or 130 °C) for an hour. The result of the test was analysed by proton NMR.

General procedure for hydrolysis for the superbase ionic liquid (I): In a vial, 1 molar equivalent of SBIL was mixed with 1 molar equivalent of water. Then the vial was heated to 90 °C (or 130 °C) for an hour. The result of the test was analysed by proton NMR.

General procedure for cellulose dissolution into the SBILs (J): In a vial, 0.9 g of SBIL (90 wt%) with 0.1 g of kraft pulp cellulose (10 wt%) were mixed and heated to 85 °C for 3 h. All the samples were studied under a microscope with a polarised light.

1. Synthesis of 7-methyl-1,5,7-Triazabicyclo[4.4.0]dec-5-enium Acetate (1).

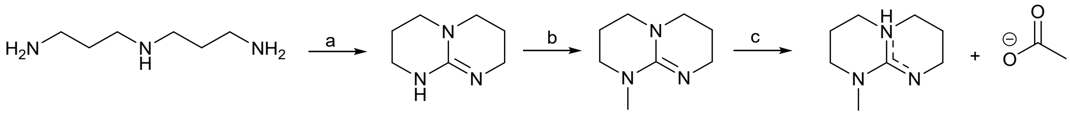

Scheme S1: General overview of the 7-methyl-1,5,7-Triazabicyclo[4.4.0]dec-5-enium Acetate synthesis.

a. Cyclisation of 1,3-Propanediamine (a):

The synthesis followed the general procedure **E** with 20 g (152 mmol) of 1,3-Propanediamine 14.56 g (152 mmol) of guanidine hydrochloride and 12.2 g (304 mmol) of sodium hydroxide the product was obtained as a white solid (Yield: 19.67 g, 93%).

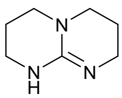

Characterization: ^{1}**H NMR** (600 MHz; DMSO-d_6) δ_h 3.05 – 2.97 (m, 8H), 1.73 (p, J = 5.9 Hz, 4H). **HRMS** calculated: 139.1109 found: 139.1105

b. Methylation of 1,5,7-Triazabicyclo[4.4.0]dec-5-ene.

The synthesis followed the general procedure **F** with 5 g of TBD (40 mmol) and 3.9 g (44 mmol) of dimethyl carbonate. The yellow oil was distilled (1 mmbar, 180 °C) to give a colourless liquid (Yield: 5.08 g, 83%).

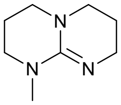

Characterization: ^{1}**H NMR** (400 MHz, DMSO-d_6) δ_h 3.21 – 3.13 (m, 2H), 3.00 (dt, J = 12.1, 6.0 Hz, 6H), 2.65 (s, 3H), 1.86 (tt, J = 6.7, 5.7 Hz, 2H), 1.72 – 1.62 (m, 2H). ^{13}C NMR (101 MHz, DMSO) δ 151.32, 48.62, 48.32, 48.26, 44.01, 37.15, 23.43, 23.20. **HRMS** calculated: 153.1266 found: 153.1265

c. Synthesis of 7-methyl-1,5,7-Triazabicyclo[4.4.0]dec-5-enium Acetate.

The synthesis followed the general procedure **G** with 2.5 g (16.31 mmol) of mTBD and 0.98 g (16.31 mmol) of acetic acid.

Characterization: ^{1}**H NMR** (400 MHz, DMSO-d_6) δ_h 2.84 – 2.72 (m, 8H), 2.50 (s, 3H), 1.44 (tdd, J = 6.8, 5.6, 4.5 Hz, 2H), 1.33 (dq, J = 7.1, 5.8 Hz, 2H), 1.13 (s, 3H).

d. Hydrolysis of 7-methyl-1,5,7-Triazabicyclo[4.4.0]dec-5-enium Acetate.

The synthesis followed the general procedure **I** with 1g (4.6 mmol, 1 equivalent) of 5-methyl-1,5,7-triazabicyclo[4.5.0]undec-6-enium Acetate and 84 µL (4.6 mmol, 1 equivalent) of water.

2. Synthesis of 5-methyl-1,5,7-triazabicyclo[4.5.0]undec-6-enium Acetate (2).

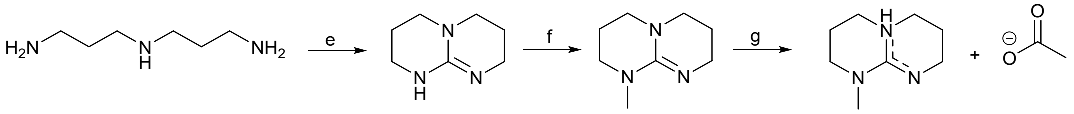

Scheme S2: General overview of the 5-methyl-1,5,7-triazabicyclo[4.5.0]undec-6-enium Acetate synthesis.

e. Cyclisation of N1-(3-aminopropyl)butane-1,4-diamine (c)

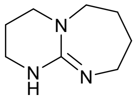

The synthesis followed the general procedure **D** with 10 g (68.8 mmol) of N1-(3-aminopropyl)butane-1,4-diamine 6.57 g (68.8 mmol) of guanidine hydrochloride and 5.5 g (137.6 mmol) of sodium hydroxide. The product was obtained as a white solid (Yield: 9.59 g, 91%).

Characterization: ^{1}H NMR (400 MHz, DMSO-d_6) δ_h 3.14 (dd, J = 6.7, 5.5 Hz, 2H), 3.11 – 2.98 (m, 4H), 2.76 (dd, J = 6.6, 3.3 Hz, 2H), 1.67 – 1.57 (m, 2H), 1.53 – 1.39 (m, 4H). **HRMS** calculated: 153.1266 found: 153.1265.

f. Methylation of 1,5,7-triazabicyclo[4.5.0]undec-5-ene.

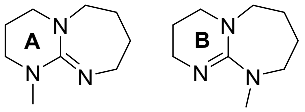

The synthesis followed the general procedure **F** with 2 g of TBU (13 mmol) and 1.52 g (16.5 mmol) of dimethyl carbonate. The yellow oil was distilled (1 mmbar, 180 °C) to give a colourless liquid (Yield: 0.5 g, 23%).

Characterization: ^{1}H NMR (400 MHz, DMSO-d_6) δ_h 3.19 – 3.07 (m, 2H), 3.09 – 3.01 (m, 4H), 2.90 – 2.82 (m, 2H), 2.68 (s, 3H), 1.78 (qd, J = 7.3, 5.5 Hz, 2H), 1.59 – 1.49 (m, 2H), 1.32 (dq, J = 8.0, 5.8 Hz, 2H). ^{13}C NMR (101 MHz, DMSO) δ 158.84, 53.31, 50.58, 48.05, 47.98, 37.83, 28.85, 27.74, 21.41. Isomers ratio A:B ➔ 100:0 **HRMS** calculated: 167.1422 found: 167.1419

g. Synthesis of 5-methyl-1,5,7-triazabicyclo[4.5.0]undec-6-enium Acetate.

The synthesis followed the general procedure **G** with 3 g (17,9 mmol) of mTBU and 1,07 g (17.9 mmol) of acetic acid.

Characterization: ^{1}H NMR (400 MHz, DMSO-d_6) δ_h 3.30 (dt, J = 5.9, 2.0 Hz, 6H), 3.29 – 3.14 (m, 2H), 3.06 (s, 3H), 1.94 – 1.77 (m, 2H), 1.61 (s, 6H), 1.53 – 1.43 (m, 2H).

h. Hydrolysis of 5-methyl-1,5,7-triazabicyclo[4.5.0]undec-6-enium Acetate.

The synthesis followed the general procedure **I** with 1g (4.4 mmol, 1 equivalent) of 5-methyl-1,5,7-triazabicyclo[4.5.0]undec-6-enium Acetate and 79 µL (4.4 mmol, 1 equivalent) of water.

3. Synthesis of 5-methyl-1,5,7-triazabicyclo[4.3.0]non-6-enium Acetate (3).

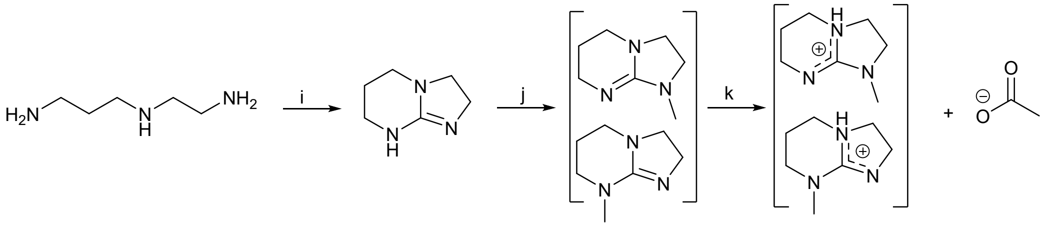

Scheme S3: General overview of the 5-methyl-1,5,7-triazabicyclo[4.3.0]non-6-enium Acetate synthesis.

i. Cyclisation of N1-(2-aminoethyl)propane-1,3-diamine (b).

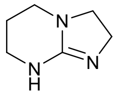

The synthesis followed the general procedure **E** with 146.5 g (1250 mmol) of N1-(2-aminoethyl)propane-1,3-diamine 0.43 g (2500 mmol) of toluene sulphonic acid monohydrate, 21.02 g of (250 mmol) dicyandiamine in 74.40 g (636 mmol). The product was obtained as a white solid (Yield: 125 g, 80%).

Characterization: **^{1}H NMR** (400 MHz, DMSO-d_6) δ_h 3.20 (t, J = 7.7 Hz, 2H), 3.09 (dd, J = 8.1, 6.3 Hz, 2H), 3.03 (t, J = 5.8 Hz, 2H), 2.98 (t, J = 5.8 Hz, 2H), 1.75 (p, J = 5.9 Hz, 2H). **HRMS** calculated: 125.0953 found: 125.0952

j. Methylation of 1,5,7-triazabicyclo[4.3.0]non-5-ene.

The synthesis followed the general procedure **F** with 102.5 g (819 mmol) of TBN and 129 g (1640 mmol) of dimethyl carbonate. The product was obtained as a colourless liquid (1 mbar, 75 °C, Yield: 85.5 g, 85%).

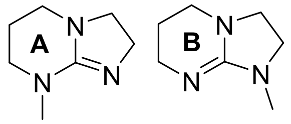

Characterization: **^{1}H NMR** (400 MHz, DMSO-d_6) δ_h 3.26 (td, J = 8.0, 0.8 Hz, 2H), 3.13 – 3.03 (m, 8H), 3.00 – 2.96 (m, 2H), 2.95 – 2.89 (m, 2H), 2.74 (s, 3H), 2.57 (s, 3H), 1.95 – 1.85 (m, 2H), 1.68 – 1.57 (m, 2H). ^{13}C NMR (101 MHz, DMSO) δ 161.21, 155.57, 53.25, 49.13, 48.55, 47.52, 46.95, 46.40, 44.18, 43.14, 37.54, 32.80, 22.71, 22.03. Isomers ratio A:B ➔ 60:40 **HRMS** calculated: 139.1109 found: 139.1105.

k. Synthesis of 5-methyl-1,5,7-triazabicyclo[4.3.0]non-6-enium Acetate.

The synthesis followed the general procedure **G** with 2.1279 g (15.28 mmol) of mTBN and 0.918 g (15.28 mmol) of acetic acid the product was obtained as a pale-yellow liquid.

Characterization: **^{1}H NMR** (400 MHz, DMSO-d_6) δ_h 3.51 – 3.33 (m, 9H), 3.32 – 3.15 (m, 6H), 3.12 (t, J = 5.8 Hz, 2H), 2.93 (s, 3H), 2.85 (s, 3H), 1.95 (p, J = 5.9 Hz, 2H), 1.86 – 1.76 (m, 2H), 1.66 (s, 6H).

l. Hydrolysis of 5-methyl-1,5,7-triazabicyclo[4.3.0]non-6-enium Acetate.

The synthesis followed the general procedure **I** with 1g (5 mmol, 1 equivalent) of 5-methyl-1,5,7-triazabicyclo[4.3.0]non-6-enium Acetate and 90 µL (5 mmol, 1 equivalent) of water.

4. Synthesis of 4-methyl-1,4,6-triazabicyclo[3.3.0]oct-5-ene Acetate (4):

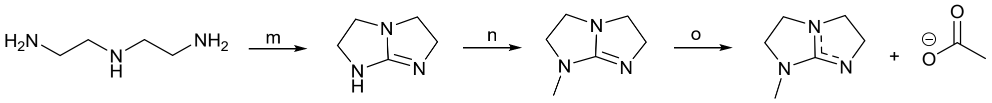

Scheme S3: General overview of the 4-methyl-1,4,6-triazabicyclo[3.3.0]oct-5-ene Acetate synthesis.

m. Cyclisation of N1-(2-aminoethyl)ethane-1,2-diamine (d)

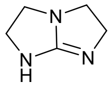

The synthesis followed the general procedure **E** 100 g (969.27 mmol) of N1-(2-aminoethyl)ethane-1,2-diamine with 0.1 g (5.8 mmol) of *p*-toluenesulphonic acid monohydrate and 16.3 g of (193.8 mmol) dicyandiamine mixed with 50 g (484 mmol) of diethylenetriamine. The product obtained as a white solid (Yield: 62.48 g, 58%).

Characterization: ^{1}**H NMR** (400 MHz, DMSO-d_6) δ_h 3.60 (t, J = 7.0 Hz, 4H), 2.92 (t, J = 7.0 Hz, 4H). **HRMS** calculated: 111.0796 found: 111.0796.

n. Methylation of 1,4,6-triazabicyclo[3.3.0]oct-4-ene.

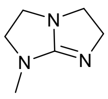

The synthesis followed the general procedure **F** with 5 g (44.9 mmol,) of TBO and 6.07 g (67.4 mmol) of dimethyl carbonate were added with a syringe. The product obtained as a colourless liquid (1 mbar, 100°C, Yield: 2.41 g, 43%).

Characterization: ^{1}**H NMR** (400 MHz, DMSO-d_6) δ_h 3.67 (t, J = 7.3 Hz, 2H), 3.51 (t, J = 6.7 Hz, 2H), 2.96 (t, J = 6.7 Hz, 2H), 2.92 (t, J = 7.3 Hz, 2H), 2.69 (s, 3H). ^{13}C NMR (101 MHz, DMSO) δ 169.64, 58.50, 53.83, 51.64, 46.31, 32.86. **HRMS** calculated: 125.0953 found: 125.0954.

o. Synthesis of 4-methyl-1,4,6-triazabicyclo[3.3.0]oct-5-enium Acetate.

The synthesis followed the general procedure **G** with 1.713 g (9.25 mmol) of mTBO and 0.55 g (9.25 mmol) of acetic acid. The product was obtained as a pale-yellow liquid.

Characterization: ^{1}**H NMR** (400 MHz, DMSO-d_6) δ_h 3.76 (t, J = 7.5 Hz, 2H), 3.65 (t, J = 7.1 Hz, 2H), 3.16 – 3.05 (m, 4H), 2.79 (s, 3H), 1.79 (s, 3H).

p. Hydrolysis of 4-methyl-1,4,6-triazabicyclo[3.3.0]oct-5-enium Acetate.

The synthesis followed the general procedure **I** with 1g (5.4 mmol, 1 equivalent) of 4-methyl-1,4,6-triazabicyclo[3.3.0]oct-5-enium Acetate and 97 µL (5.4 mmol, 1 equivalent) of water.

5. Synthesis of 7-methyl-3,3-dimethyl-9,9-dimethyl-1,5,7-Triazabicyclo[4.4.0]dec-5-enium Acetate (5).

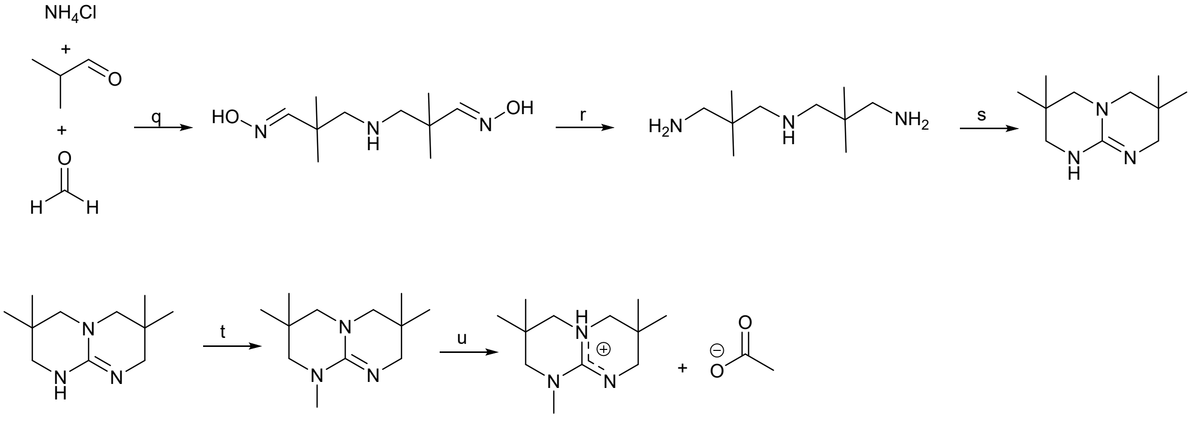

Scheme S4: General overview of the 7-methyl-3,3-dimethyl-9,9-dimethyl-1,5,7-Triazabicyclo[4.4.0]dec-5-enium Acetate synthesis.

q. Synthesis of 3,3′-Iminobis[2,2-dimethylpropanal] 1,1′-dioxime.

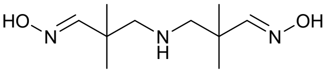

In a 2L flask, 158.15 g (1390 mmol 1 equivalent) of isobutyraldehyde, 45 g (1500 mmol, 1.08 equivalent) of paraformaldehyde, 33.4 g (625 mmol, 0.45 equivalent), 20 mL of water and 1 mL of hydrochloric acid were charged, stirred and heated to 90 °C during 4.5 h. To the reaction, 100 g (1430 mmol, 1.03 equivalent) of Hydroxylamine hydrochloride were dissolved in a mixture of 1.3 L of 96% ethanol with 400 mL of water and added to the flask over a period of 30 min. The final mixture was reflux during 30 min and cooled overnight. The day after, the solvent is dried and 50 g (170 mmol, 1 equivalent) of white solid were dissolved in methanol and mixed with 13.88 g (340 mmol, 2 equivalents) of NaOH. After 1 h, the white solid was filtered with a Buchner fritted filter (porosity 4) and the methanol dried. The product obtained as a white solid (Yield: 20.8 g, 57%).

Characterization: ^{1}H NMR (600 MHz, DMSO-d_6) δ_h 7.19 (s, 2H), 2.43 (s, 2H), 2.42 (s, 2H), 0.97 (s, 12H). The obtained data are in agreement with the published literature. [6]

r. Synthesis of N1-(3-amino-2,2-dimethylpropyl)-2,2-dimethylpropane-1,3-diamine.

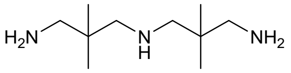

In a Parr reactor, 12.14 g (78 mmol, 1 equivalent) of triamine derivative were dissolved in 40 mL of 96% ethanol and mixed with 20 mL solution of aqueous ammonia then 1.2 g of Raney nickel (10 wt% of the starting material) were added. The reactor was loaded with 50 bars of hydrogen, heated up to 50 °C and stirred overnight. The day after, the catalyst was filtered off and the ethanol/water evaporated with a rotavapor. The product was obtained as a pale- yellow liquid and used without further purification (Yield: 14.61 g, 65%).

Characterization: ^{1}H NMR (400 MHz, DMSO-d_6) δ_h 2.33 (s, 4H), 2.28 (s, 4H), 0.77 (s, 12H). The obtained data are in agreement with the published literature. [6]

s. Cyclisation of N1-(3-amino-2,2-dimethylpropyl)-2,2-dimethylpropane-1,3-diamine.

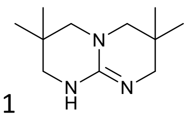

The cyclisation was done by following the general procedure **D** with 12.85 g (65 mmol, 1 equivalent) of triamine, 6.55 g (65 mmol, 1 equivalent) of guanidine hydrochloride and 5.26 g (130 mmol, 2 equivalents) of sodium hydroxide (Yield: 10.6 g, 84%).

Characterization: ^{1}**H NMR** (300 MHz, DMSO-d_6) δ_h 2.96 (s, 4H), 2.88 (s, 4H), 0.97 (s, 12H). The obtained data are in agreement with the published literature. [6]

t. Methylation of 3,3-dimethyl-9,9-dimethyl-1,5,7-Triazabicyclo[4.4.0]dec-5-ene.

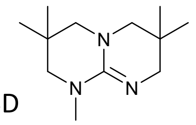

The methylation was done by following the general procedure **F** with 19.93 g of dm3-TBD (102 mmol, 1 equivalent) and 12 g (132 mmol, 1.3 equivalents) of dimethyl carbonate The day after, the solvent was dried and the yellow oil was distilled (1mmbar, 180 °C, Yield: 14.94 g, 70%) to give a colourless liquid.

Characterization: ^{1}**H NMR** (600 MHz, DMSO-d_6) δ_h 2.87 (s, 1H), 2.87 (s, 2H), 2.76 (s, 2H), 2.76 (s, 2H), 2.73 (s, 2H), 2.73 (s, 2H), 2.68 (s, 1H), 2.66 (s, 3H), 2.66 (s, 2H), 0.98 (s, 6H), 0.86 (s, 6H). ^{13}C NMR (101 MHz, DMSO) δ 149.05, 60.75, 60.58, 60.02, 56.95, 37.42, 30.10, 28.98, 25.30, 25.14. **HRMS** calculated: 195.1735 found: 195.1741.

u. Synthesis of 7-methyl-3,3-dimethyl-9,9-dimethyl-1,5,7-Triazabicyclo[4.4.0]dec-5-enium Acetate.

The SBILs was synthesized by following the general procedure **G** with 2 g of 7-methyl-3,3-dimethyl-9,9-dimethyl-1,5,7-Triazabicyclo[4.4.0]dec-5-ene (9.55 mmol) and 0.57 g of acetic acid (9.55 mmol). The product was obtained as a white to pale yellow solid.

Characterization: ^{1}**H NMR** (600 MHz, DMSO-d_6) δ_h 3.03 (s, 2H), 3.00 (s, 3H), 2.98 (s, 4H), 2.97 (s, 2H), 1.61 (s, 3H), 0.97 (s, 6H), 0.96 (s, 6H).

v. Hydrolysis of *7-methyl-3,3-dimethyl-9,9-dimethyl-1,5,7-Triazabicyclo[4.4.0]dec-5-enium Acetate.*

The synthesis followed the general procedure **I** with 1g (3.7 mmol, 1 equivalent) of 7-methyl-3,3-dimethyl-9,9-dimethyl-1,5,7-Triazabicyclo[4.4.0]dec-5-enium Acetate and 67 µL (3.7 mmol, 1 equivalent) of water.

6. Synthesis of 7-methyl-3,3-dimethyl-10-methyl-1,5,7-Triazabicyclo[4.4.0]dec-5-enium Acetate (6).

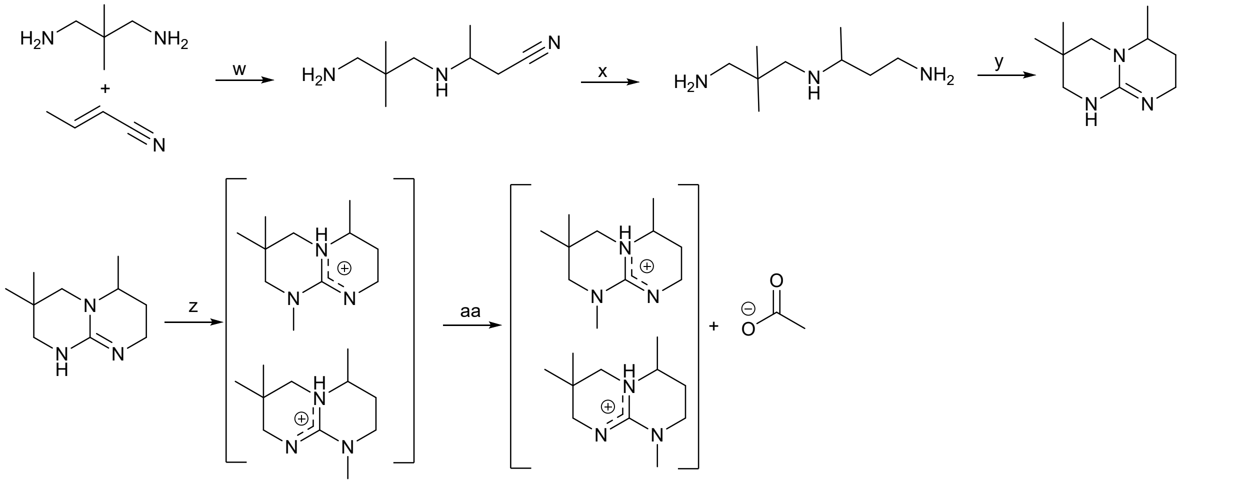

Scheme S5: General overview of the 7-methyl-3,3-dimethyl-10-methyl-1,5,7-Triazabicyclo[4.4.0]dec-5-enium Acetate synthesis.

w. Synthesis of 3-((3-amino-2,2-dimethylpropyl)amino)butanenitrile.

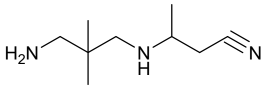

The synthesis followed the general procedure **A** with 10 g of crotonitrile (g) (160 mmol, 1 equivalent) and 98 g of 2,2-dimethylpropane-1,3-diamine (e) (960 mmol, 6 equivalents). The product is obtained as a colourless liquid (Yield: 17.60 g, 65%).

Characterization: ^{1}**H NMR** (400 MHz, DMSO-d_6) δ_h 2.81 (h, J = 6.1 Hz, 1H), 2.54 (d, J = 5.6 Hz, 2H), 2.35 – 2.24 (m, 4H), 1.09 (d, J = 6.3 Hz, 3H), 0.77 (s, 6H). **HRMS** calculated: 169.1579 found: overlapping with PFK-peak.

x. Synthesis of N3-(3-amino-2,2-dimethylpropyl)butane-1,3-diamine.

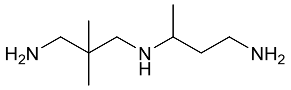

The synthesis followed the general procedure **C** with 15.7 g (90 mmol, 1 equivalent) of triamine derivative, 30 mL solution of aqueous sodium hydroxide (4.1 g, 99 mmol, 1.1 equivalents), 1.5 g of Raney nickel (10 wt% of the starting material). The product was obtained as a yellow liquid (Yield: 9.81 g, 63%).

Characterization: ^{1}**H NMR** (400 MHz, DMSO-d_6) δ_h 2.66 – 2.46 (m, 4H), 2.38 – 2.15 (m, 4H), 1.51 – 1.36 (m, 1H), 1.29 (ddt, J = 13.8, 7.7, 6.2 Hz, 1H), 0.94 (d, J = 6.3 Hz, 3H), 0.76 (s, 6H). **HRMS** calculated: 173.1892 found: overlapping with PFK-peak.

y. Cyclisation of N3-(3-amino-2,2-dimethylpropyl)butane-1,3-diamine.

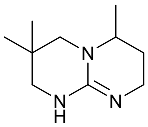

The synthesis followed the general procedure **D** with 10 g (57 mmol, 1 equivalent) of triamine, 5.51 g (57 mmol, 1 equivalent) of guanidine hydrochloride and 4.56 g (114 mmol, 2 equivalents) of sodium hydroxide (Yield: 6.06 g, 59%).

Characterization: ^{1}**H NMR** (400 MHz, DMSO-d_6) δ_h 3.14 (dddd, J = 13.0, 10.9, 7.9, 5.0 Hz, 2H), 3.07 – 2.96 (m, 2H), 2.77 – 2.66 (m, 2H), 2.64 – 2.44 (m, 2H), 1.86 – 1.69 (m, 1H), 1.53 (dq, J = 12.8, 3.8 Hz, 1H), 1.13 – 1.03 (m, 3H), 0.90 (s, 6H). Isomers ratio A:B → 59:41 **HRMS** calculated: 181.1579 found: overlapping with PFK-peak.

z. *Methylation of 3,3-dimethyl-10-methyl-1,5,7-Triazabicyclo[4.4.0]dec-5-ene.*

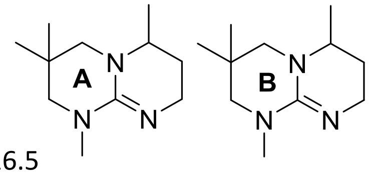

The synthesis followed the general procedure **F** with 3 g of dm3-m9-TBD (16.5 mmol, 1 equivalent) and 2.24 g (25 mmol, 1.5 equivalents) of dimethyl carbonate, The orange oil was distilled (1 mmbar, 180°C) to give a pale-yellow liquid (Yield: 2.03 g, 63%).

Characterization: ^{1}**H NMR** (400 MHz, DMSO-d_6) δ_h 3.23 – 3.09 (m, 4H), 3.06 – 2.89 (m, 2H), 2.89 – 2.81 (m, 4H), 2.70 (s, 3H), 2.66 (s, 3H), 2.00 (ddt, J = 13.0, 10.4, 5.2 Hz, 1H), 1.88 (tt, J = 6.8, 5.7 Hz, 1H), 1.73 – 1.55 (m, 1H), 1.52 – 1.41 (m, 1H), 1.08 (d, J = 6.4 Hz, 3H), 1.03 (d, J = 6.4 Hz, 3H), 0.95 (s, 6H), 0.84 (s, 6H). ^{13}C NMR (101 MHz, DMSO) δ 149.84, 148.93, 60.48, 58.16, 57.53, 56.75, 52.35, 51.51, 44.77, 37.34, 29.93, 29.30, 29.02, 28.58, 25.35, 25.11, 25.04, 24.97, 19.64, 18.93.Isomers ratio A:B →54:46 **HRMS** calculated: 195.1735 found: 195.1741.

aa. *Synthesis of 7-methyl-3,3-dimethyl-10-methyl-1,5,7-Triazabicyclo[4.4.0]dec-5-enium Acetate.*

The synthesis followed the general procedure **G** with 0.7 g of 3,3-dimethyl-10-methyl-1,5,7-Triazabicyclo[4.4.0]dec-5-ene (2.9 mmol) and 0.25 g (2.9 mmol) of acetic acid. The product was obtained as a pale-yellow oil.

Characterization: ^{1}**H NMR** (400 MHz, DMSO-d_6) δ_h 3.49 – 3.31 (m, 4H), 3.30 – 3.16 (m, 4H), 3.02 – 2.90 (m, 12H), 2.08 – 1.69 (m, 4H), 1.59 (s, 6H), 1.16 (dd, J = 6.5, 2.4 Hz, 6H), 1.01 – 0.86 (m, 12H).

bb. Hydrolysis of *7-methyl-3,3-dimethyl-10-methyl-1,5,7-Triazabicyclo[4.4.0]dec-5-enium Acetate.*

The synthesis followed the general procedure **I** with 1g (3.9 mmol, 1 equivalent) of 77-methyl-3,3-dimethyl-10-methyl-1,5,7-Triazabicyclo[4.4.0]dec-5-enium Acetate and 70 µL (3.9 mmol, 1 equivalent) of water.

7. Synthesis of 7-methyl-3,3-dimethyl-1,5,7-Triazabicyclo[4.4.0]dec-5-enium Acetate (7).

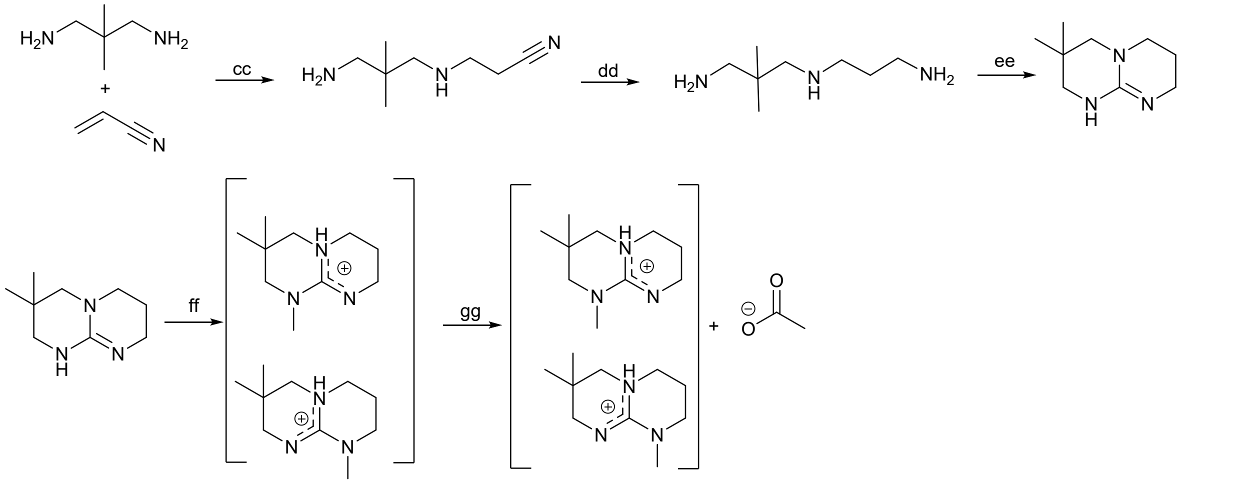

Scheme S6: General overview of the 7-methyl-3,3-dimethyl-1,5,7-Triazabicyclo[4.4.0]dec-5-enium Acetate synthesis.

cc. Synthesis of 3-((3-amino-2,2-dimethylpropyl)amino)propanenitrile.

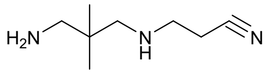

The synthesis followed the general procedure A with 5.9 g of acrylonitrile (e) (112 mmol, 1 equivalent) and 68.6 g of (672 mmol, 6 equivalents) propane-1,3-diamine (i). The product is obtained as a colourless liquid (Yield: 12.51 g, 66%).

Characterization: ^{1}**H NMR** (400 MHz, DMSO-d_6) δ_h 2.73 (t, J = 6.6 Hz, 2H), 2.56 (t, J = 6.6 Hz, 2H), 2.33 (s, 2H), 2.29 (s, 2H), 0.78 (s, 6H). **HRMS** calculated: 155.1422 found: overlapping with PFK-peak.

dd. Synthesis of N1-(3-aminopropyl)-2,2-dimethylpropane-1,3-diamine.

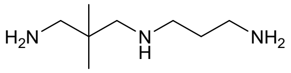

Option 1: Hydrogenation of 3-((3-amino-2,2-dimethylpropyl)amino)propanenitrile following the general procedure **C** with 12.14 g (78 mmol, 1 equivalent) of triamine, 20 mL solution of aqueous sodium hydroxide (3.44 g NaOH, 86 mmol, 1.1 equivalents), 1.2 g of Raney nickel (10wt% of the starting material). The product was obtained as a pale-yellow liquid (Yield: 11. 48 g, 85%).

Option 2: The product can also be synthesized using the general procedure **B** with 7 g (32 mmol, 1 equivalent) of 3-Bromopropylamine hydrobromide with 13 g (117 mmol, 3.6 equivalents) of 2,2-Dimethyl-1,3-propanediamine and 18 g (320 mmol, 10 equivalents) of KOH. The product was obtained as a pale-yellow liquid (Yield: 3.6 g, 65%).

Characterisation: ^{1}**H NMR** (400 MHz, DMSO-d_6) δ_h 2.53 (t, J = 6.8 Hz, 4H), 2.30 (s, 2H), 2.24 (s, 2H), 1.45 (p, J = 6.8 Hz, 2H), 0.76 (s, 6H). **HRMS** calculated: 159.1735 found: 159.1731.

ee. Cyclisation of N1-(3-aminopropyl)-2,2-dimethylpropane-1,3-diamine.

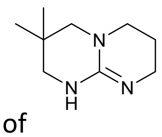

The synthesis followed the general procedure **D** with 10 g (63 mmol, 1 equivalent) of triamine, 6 g (63 mmol, 1 equivalent) of guanidine hydrochloride and 5.04 g (126 mmol, 2 equivalent) of

sodium hydroxide. The product was distilled under vacuum (1mmbar, 180-200 °C) to give a white solid (Yield: 6.84 g, 65%).

Characterisation: ^{1}**H NMR** (400 MHz, DMSO-d_6) δ_h 3.02 (dt, J = 12.4, 5.9 Hz, 4H), 2.74 (s, 2H), 2.69 (s, 2H), 1.75 (p, J = 6.0 Hz, 6H), 0.90 (s, 1H). **HRMS** calculated: 167.1422 found: 167.1425.

ff. Methylation of 3,3-dimethyl-1,5,7-Triazabicyclo[4.4.0]dec-5-ene.

The synthesis followed the general procedure **F** with 2.32 g of dm3-TBD (12 mmol, 1 equivalent), 1.49 g (16mmol, 1.3 equivalents) of dimethyl carbonate. The orange oil was distilled (1 mmbar, 180 °C) to give a pale-yellow liquid (Yield: 1.5 g, 70%).

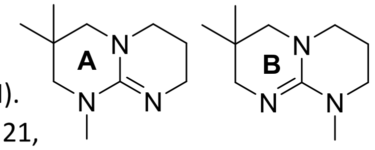

Characterisation: ^{1}**H NMR** (400 MHz, DMSO-d_6) δ_h 3.20 – 3.16 (m, 2H), 3.06 – 2.96 (m, 6H), 2.86 (s, 2H), 2.74 (s, 2H), 2.71 (s, 2H), 2.67 (s, 3H), 2.66 (s, 3H), 1.93 – 1.82 (m, 2H), 1.68 (p, J = 5.8 Hz, 2H), 0.97 (s, 6H), 0.85 (s, 6H). ^{13}C NMR (101 MHz, CDCl3) δ 150.63, 60.09, 59.94, 59.91, 54.02, 48.47, 48.21, 41.87, 37.97, 37.91, 29.36, 28.33, 24.55, 24.52, 22.21, 22.02. Isomers ratio A:B ➔ 45:55 **HRMS** calculated: 81.1579 found: 181.1580.

gg. Synthesis of 7-methyl-3,3-dimethyl-1,5,7-Triazabicyclo[4.4.0]dec-5-enium Acetate.

The synthesis followed the general procedure **G** with of dm3-mTBD (2.7 mmol) 0.165 g (2.7 mmol) of acetic acid. The product was obtained as a pale yellow to orange viscous liquid.

Characterisation: ^{1}**H NMR** (500 MHz, DMSO-d_6) δ_h 3.25 (ddt, J = 17.1, 11.9, 5.9 Hz, 9H), 3.03 – 2.92 (m, 15H), 1.93 (p, J = 6.0 Hz, 2H), 1.84 (p, J = 5.9 Hz, 2H), 1.60 (s, 3H), 0.97 (s, 6H), 0.95 (s, 6H).

hh. Hydrolysis of *7-methyl-3,3-dimethyl-1,5,7-Triazabicyclo[4.4.0]dec-5-enium Acetate.*

The synthesis followed the general procedure **I** with 1g (4.1 mmol, 1 equivalent) of 7-methyl-3,3-dimethyl-1,5,7-Triazabicyclo[4.4.0]dec-5-enium Acetate and 75 µL (4.1 mmol, 1 equivalent) of water.

8. Synthesis of 7-methyl-2-methyl-1,5,7-Triazabicyclo[4.4.0]dec-5-enium Acetate (8).

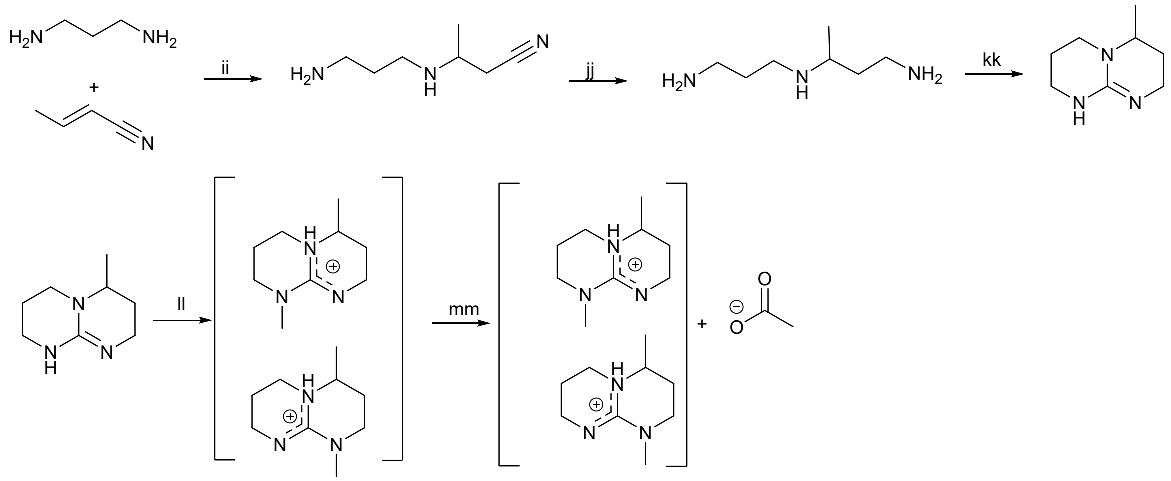

Scheme S6: General overview of the 7-methyl-2-methyl-1,5,7-Triazabicyclo[4.4.0]dec-5-enium Acetate_synthesis.

ii. Synthesis of 3-((3-aminopropyl)amino)butanenitrile.

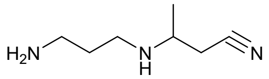

The synthesis followed the general procedure **A** with 2 g of crotonitrile (g) (29 mmol, 1 equivalent), and 12.9 g of propane-1,3-diamine (i) (174mmol, 6 equivalents). The product is obtained as a colourless liquid (Yield: 2.58 g, 63%).

Characterisation: ^{1}**H NMR** (400 MHz, CDCl3) δ_h 2.87 (dtd, J = 12.0, 6.4, 5.5 Hz, 0H), 2.62 (t, J = 6.7 Hz, 2H), 2.53 (t, J = 6.9 Hz, 2H), 2.30 (dd, J = 5.6, 1.2 Hz, 2H), 1.46 (p, J = 6.8 Hz, 2H), 1.08 (d, J = 6.4 Hz, 3H). **HRMS** calculated: 141.1266 found: 141.1265.

jj. Synthesis of N3-(3-aminopropyl)butane-1,3-diamine.

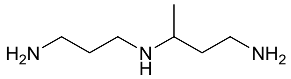

The synthesis followed the general procedure **C** with 5 g (35 mmol, 1 equivalent) of triamine derivative, 20ml solution of aqueous sodium hydroxide (1.55 g NaOH, 38 mmol, 1.1 equivalent) and 0.5 g of Raney nickel (10 wt% of the starting material) The product was obtained as a pale-yellow liquid (Yield: 3.30 g, 65%).

Characterisation: ^{1}**H NMR** (400 MHz, DMSO-d_6) δ_h 2.64 – 2.52 (m, 6H), 1.43 (p, J = 6.9 Hz, 4H), 0.93 (d, J = 6.3 Hz, 3H). **HRMS** calculated: 145.1579 found: overlapping with PFK-peak.

kk. Cyclisation of N3-(3-aminopropyl)butane-1,3-diamine.

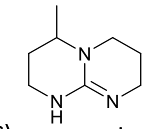

The synthesis followed the general procedure **D** with 4.35 g (27 mmol, 1 equivalent) of triamine, 2.6 g (27 mmol, 1 equivalent) of guanidine hydrochloride, 2.18 g (54 mmol, 2 equivalents) of sodium hydroxide The product was distilled under vacuum (1mmbar, 200 °C) to give a pale-yellow solid (Yield: 2.64 g, 64%).

Characterisation: ^{1}**H NMR** (400 MHz, DMSO-d_6) δ_h 3.21 (dd, J = 11.2, 6.2 Hz, 4H), 3.06 – 2.96 (m, 2H), 2.90 (dt, J = 11.2, 5.5 Hz, 1H), 1.74 – 1.67 (m, 2H), 1.51 (dq, J = 12.9, 4.0 Hz, 1H), 1.08 (d, J = 6.4 Hz, 3H). **HRMS** calculated: 153.1266 found: overlapping with PFK-peak.

ll. Methylation of 2-methyl-1,5,7-Triazabicyclo[4.4.0]dec-5-ene.

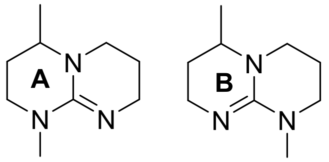

The synthesis followed the general procedure **F** with 3.6 g of m2-TBD (23 mmol, 1 equivalent), 2.32 g (25 mmol, 1.1 equivalent) of dimethyl carbonate were added and the mixture was refluxed overnight The brown oil was distilled (1 mmbar, 180 °C) to give a yellow liquid (Yield: 1.8 g, 46%).

Characterisation: ^{1}**H NMR** (400 MHz, DMSO-d_6) δ_h 3.24 – 3.08 (m, 9H), 3.06 – 2.86 (m, 6H), 2.67 (s, 3H), 2.65 (s, 3H), 1.99 (ddt, J = 13.1, 10.2, 5.2 Hz, 1H), 1.91 – 1.70 (m, 2H), 1.74 – 1.54 (m, 4H), 1.49 – 1.42 (m, 1H), 1.08 (d, J = 6.4 Hz, 3H), 1.05 (d, J = 6.4 Hz, 3H). ^{13}C NMR (101 MHz, DMSO) δ 150.93, 150.25, 52.59, 51.34, 48.25, 46.16, 46.09, 44.98, 44.05, 37.20, 37.18, 29.63, 23.62, 23.38, 19.50, 18.76. Isomers ration A:B ➔ 54:46 **HRMS** calculated: 167.1422 found: 167.1420.

mm. Synthesis of 7-methyl-2-methyl-1,5,7-Triazabicyclo[4.4.0]dec-5-enium Acetate.

The synthesis followed the general procedure **G** with 0.7 g of m2-mTBD (2.9 mmol) and 0.25 g (2.9 mmol) of acetic acid. The product was obtained as a yellow oil.

Characterisation: **^{1}H NMR** (600 MHz, DMSO-d_6) δ_h 3.54 – 3.43 (m, 2H), 3.43 – 3.31 (m, 4H), 3.30 – 3.15 (m, 9H), 2.98 (s, 3H), 2.95 (s, 3H), 2.01 (ddt, J = 13.6, 11.6, 5.3 Hz, 1H), 1.95 – 1.79 (m, 6H), 1.78 – 1.67 (m, 2H), 1.60 (s, 3H), 1.18 (dd, J = 6.3, 6.3 Hz, 6H).

nn. Hydrolysis of 7-methyl-2-methyl-1,5,7-Triazabicyclo[4.4.0]dec-5-enium Acetate.

The synthesis followed the general procedure **I** with 1g (4.3 mmol, 1 equivalent) of 7-methyl-2-methyl-1,5,7-Triazabicyclo[4.4.0]dec-5-enium Acetate and 79 µL (4.3 mmol, 1 equivalent) of water.

IV. Calculation of hydrolysis for SB and SBILs from ^{1}H NMR.

The amount of hydrolysed product was calculated according to the published formula.[32]

Integration calculation was done with MesReNova software, using the Sum method, algorithm: signal picking.

molecule	integrals value for SB	integral value for SB hydrolysed	integrals ratio*100 (%)
1	3	1	25
1.OAc	3	0.11	4
2	3	0.35	10
2.OAc	3	0	0
3	3	0.08	3
3.OAc	3	0	0
4	3	0.06	2
4.OAc	3	0.82	41
5	3	0	0
5.OAc	3	0	0
6	5,6	0.6	10
6.OAc	6	0	0
7	4.59	0.95	17
7.OAc	3	0	0
8	6	0.24	4
8.OAc	3	0	0

Table S1: integrals value and integral ratio used for calculating the level of hydrolysis of the SB and the SBILs at 90°C

molecule	integrals value for SB	integral value for SB hydrolysed	integrals ratio*100 (%)
1	3	2.98	50
1.OAc	3	0.34	10
2	3	2.1	41
2.OAc	3	2	40
3	6.19	1,47	20
3.OAc	3	0.25	8
4	3	0,39	11

4.OAc	3	9.45	76
5	3	0	0
5.OAc	3	0	0
6	6	0.67	10
6.OAc	5.79	0.22	3
7	3.52	1.09	24
7.OAc	12	0.25	2
8	6	1.47	20
8.OAc	6	0.14	2

Table S2: integrals value and integral ratio used for calculating the level of hydrolysis of the SB and the SBILs at 130°C

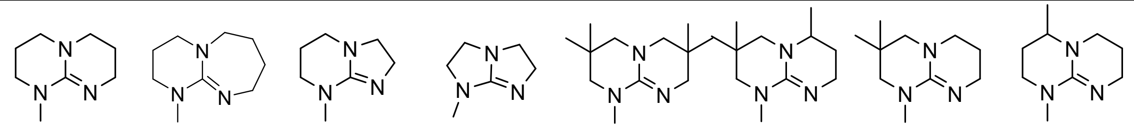

Hyd. 90° C	25	9	2	2	0	10	17	4
Hyd. 130°C	51	41	20	11	0	10	24	20
m.p	<RT	<RT	<RT	<RT	40	<RT	<RT	<RT

Table S3: This table contains the hydrolysis percentage of the free superbases. As it can be seen in Table S3, in general all the methyl substituted bicyclic guanidine bases are more stable than the commercially available mTBD. The experimental conditions and data collection are identical for the free superbase and the corresponding carboxylate salt. Reminder of experimental conditions: SB (or SBIL): Water ration is 1:1 (mol/mol), the mixture is heated to 90°C for an hour. After this, an NMR 1H proton spectra is run and the hydrolysis percentage (mol%) is calculated using integrals method.

V. <u>1D NMR spectra</u>

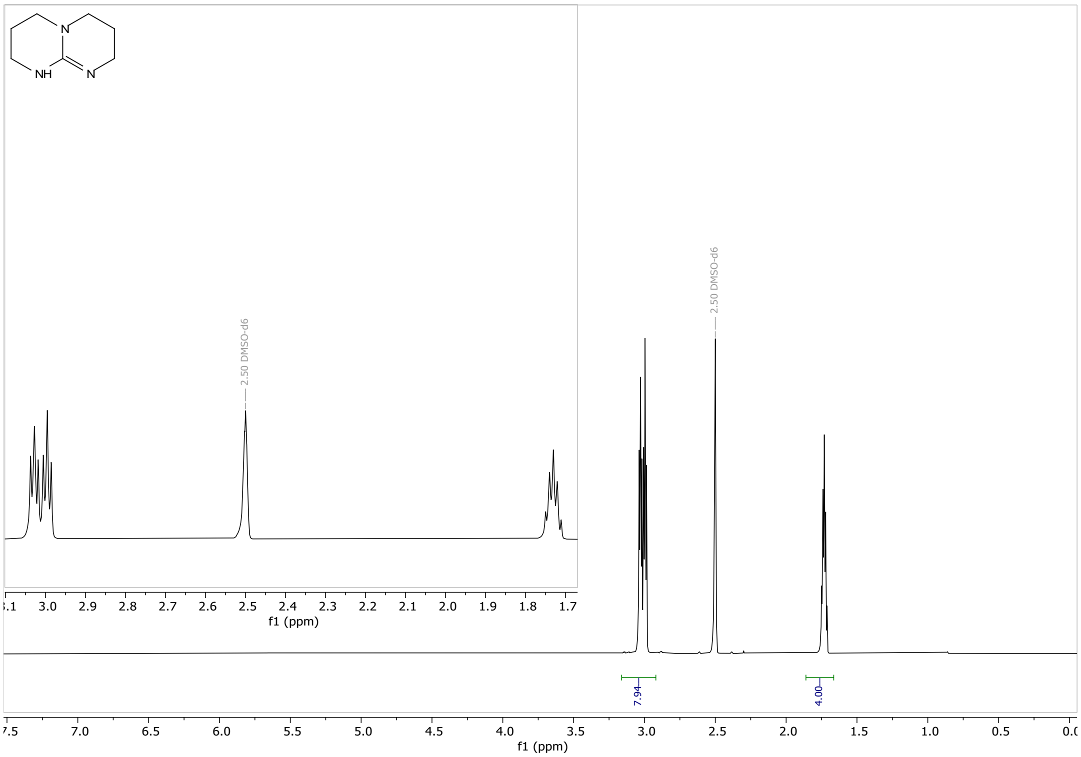

Figure S4: ^{1}H of **1,5,7-Triazabicyclo[4.4.0]dec-5-ene** in DMSO-d_6.

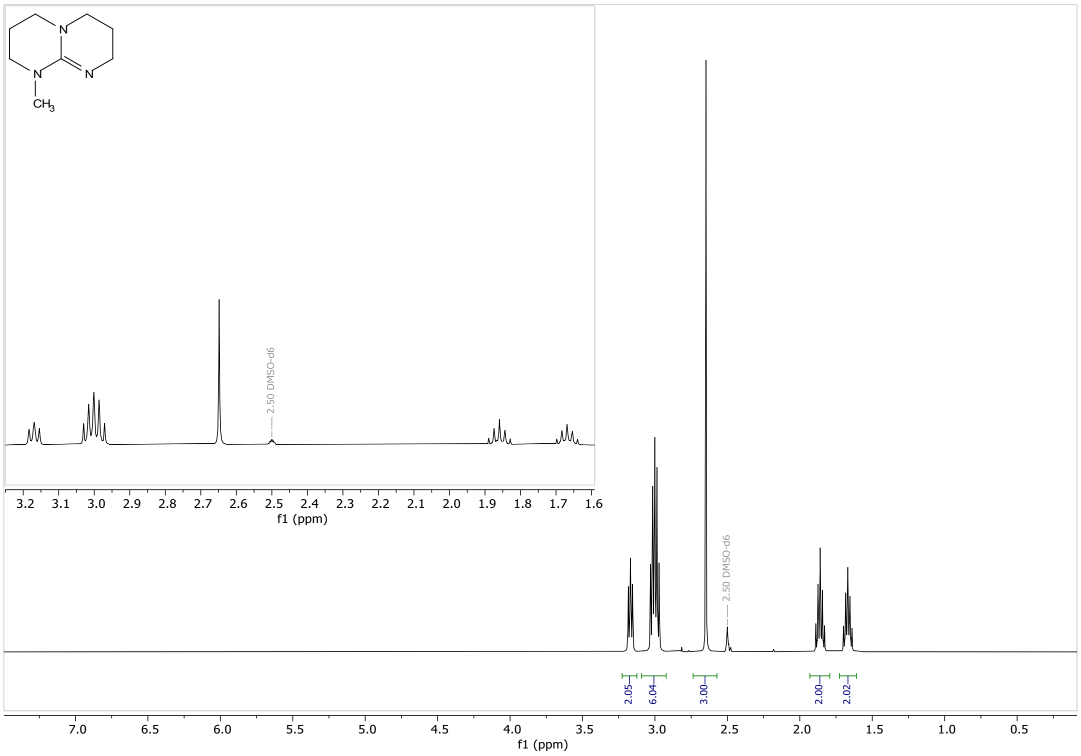

Figure S5: ^{1}H of **7-methyl-1,5,7-Triazabicyclo[4.4.0]dec-5-ene** in DMSO-d_6.

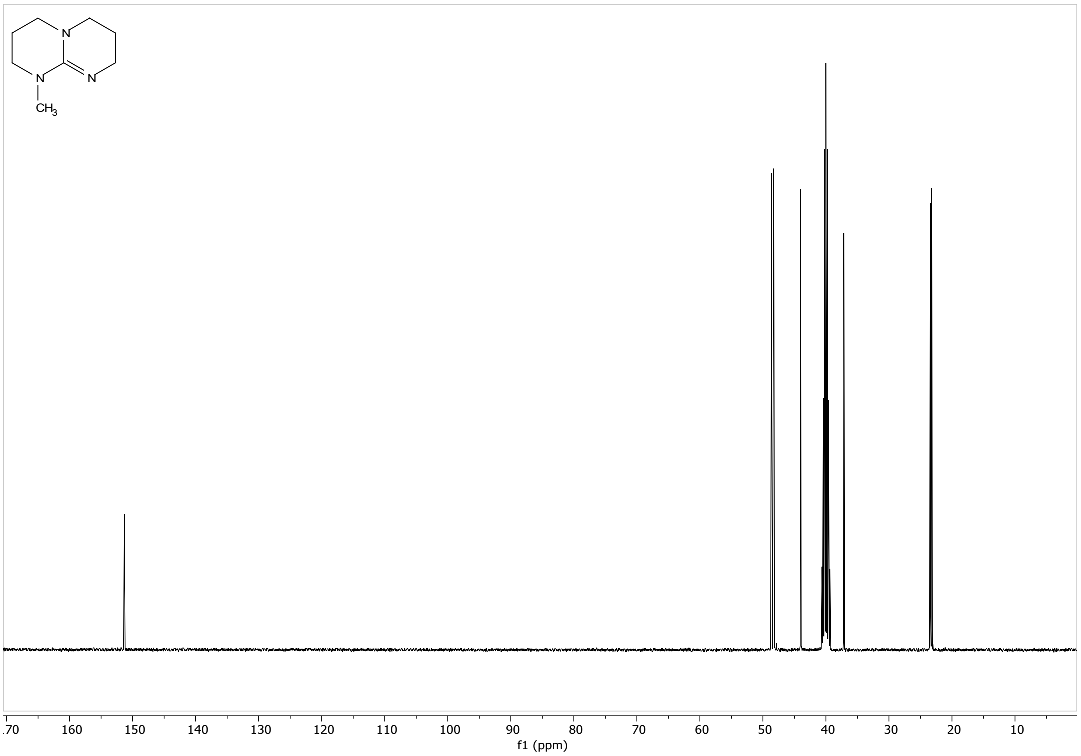

Figure S5: ^{13}C of **7-methyl-1,5,7-Triazabicyclo[4.4.0]dec-5-ene** in DMSO-d_6.

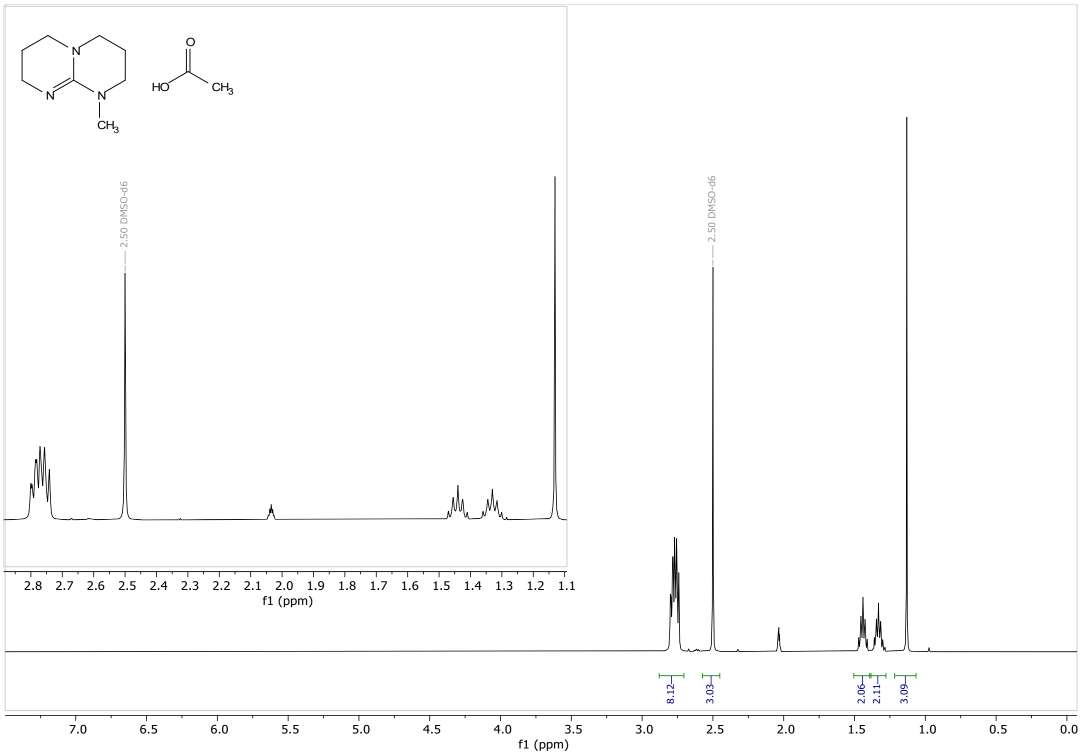

Figure S6: ^{1}H of **7-methyl-1,5,7-Triazabicyclo[4.4.0]dec-5-enium Acetate** in DMSO-d_6.

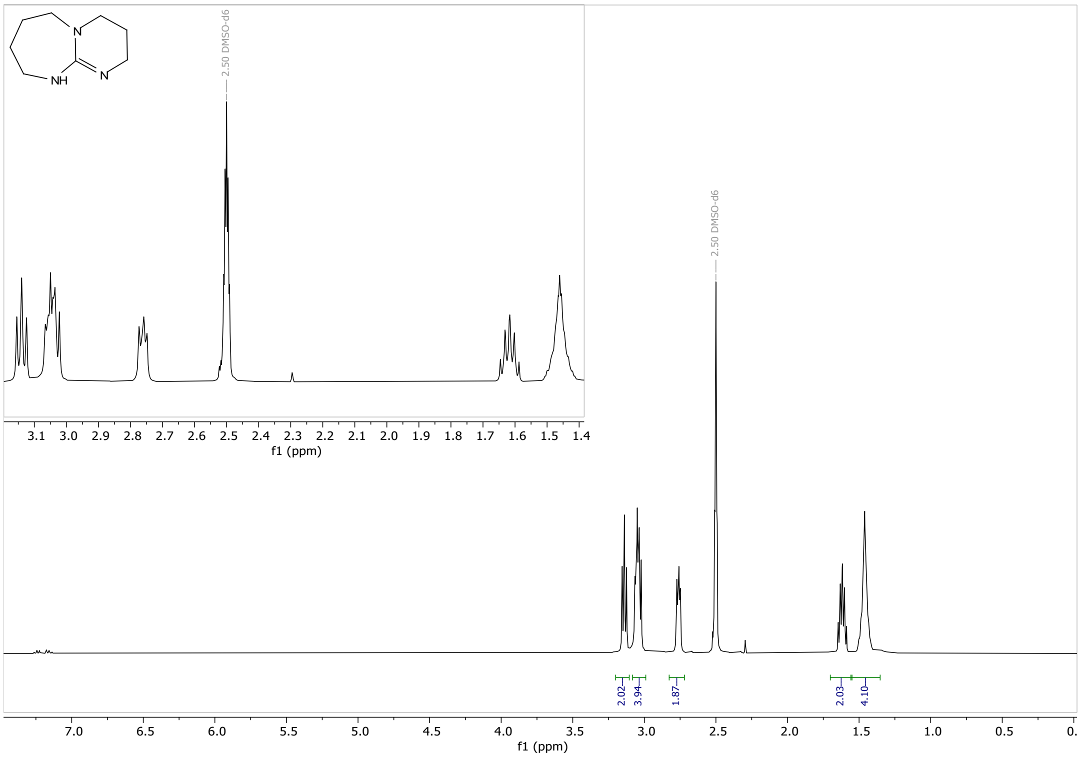

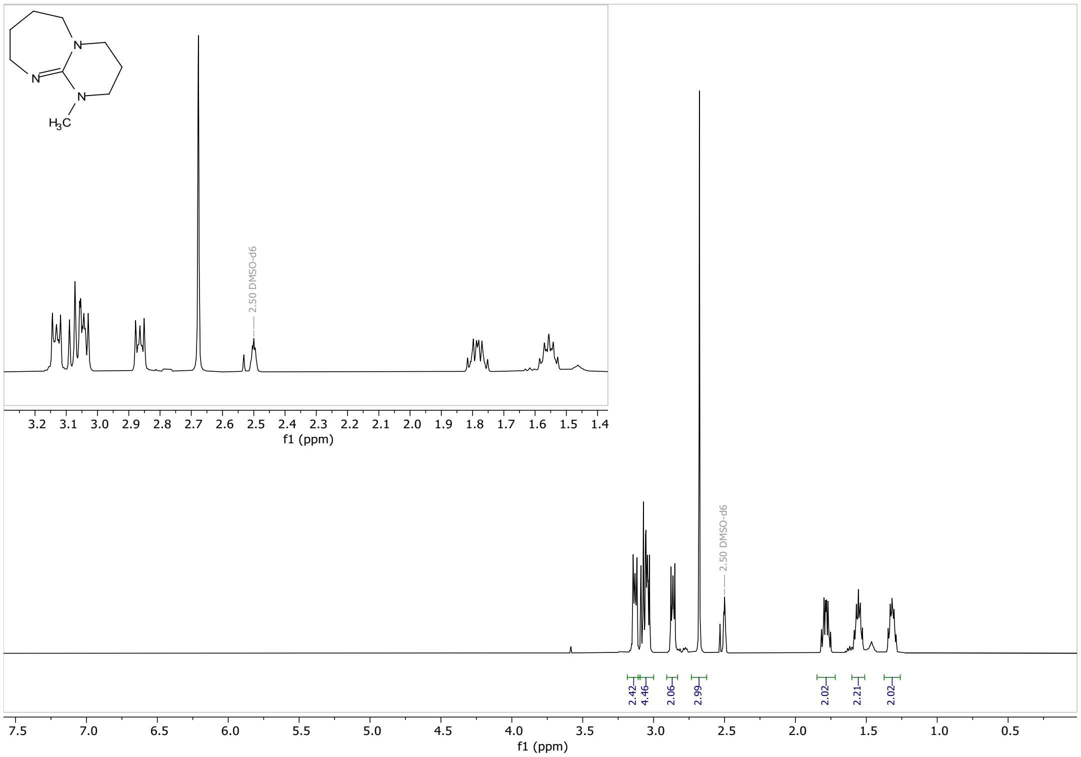

***Figure S7**:* ^{1}H of **1,5,7-triazabicyclo[4.5.0]undec-5-ene** in DMSO-d_6.

Figure S8: ^{1}H of **5-methyl-1,5,7-triazabicyclo[4.5.0]undec-5-ene** in DMSO-d$_6$.

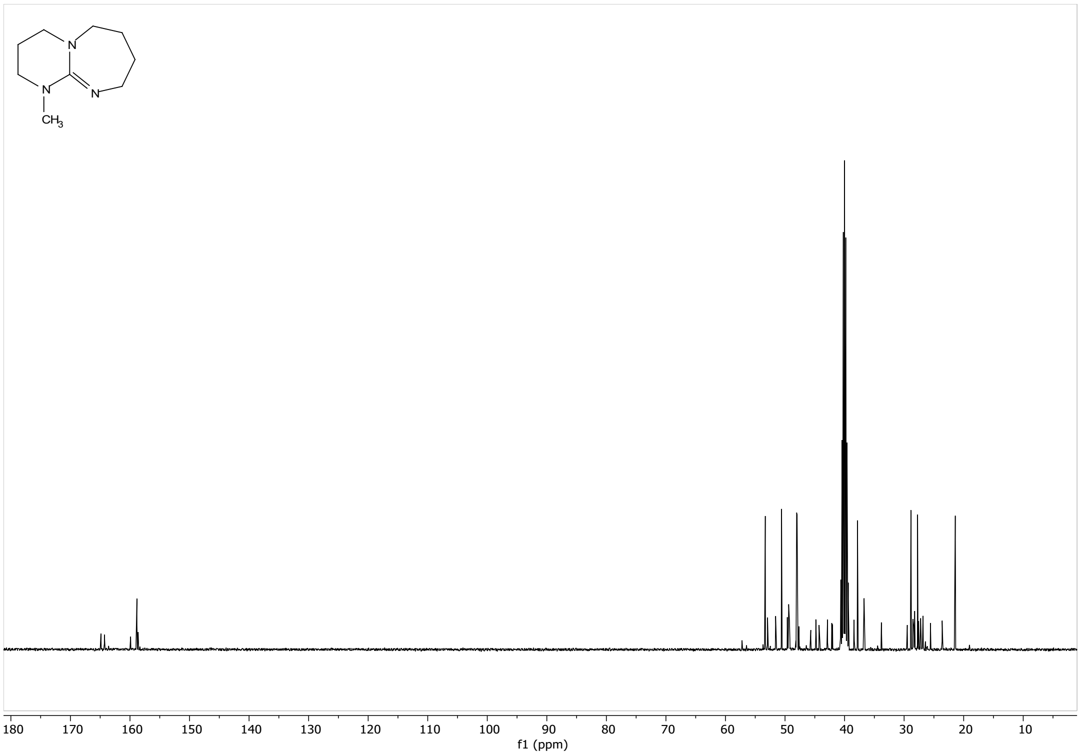

Figure S9: ^{13}C of **5-methyl-1,5,7-triazabicyclo[4.5.0]undec-5-ene** in DMSO-d$_6$.

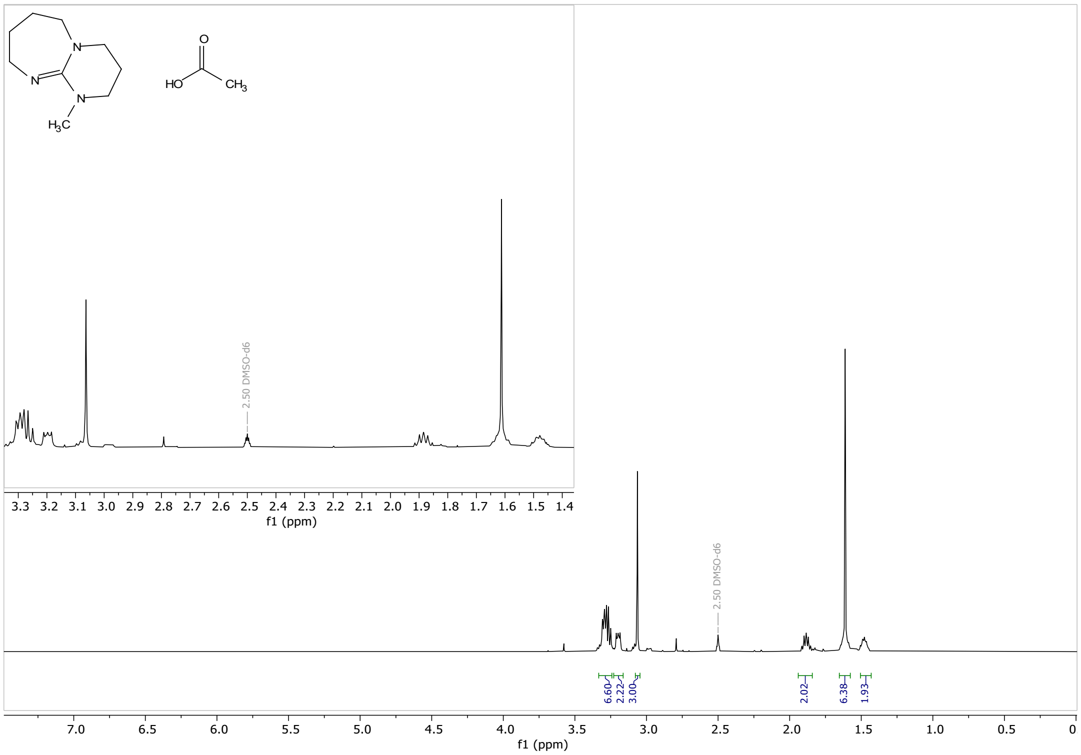

Figure S10: ^{1}H of **5-methyl-1,5,7-triazabicyclo[4.5.0]undec-6-enium Acetate** in DMSO-d_6.

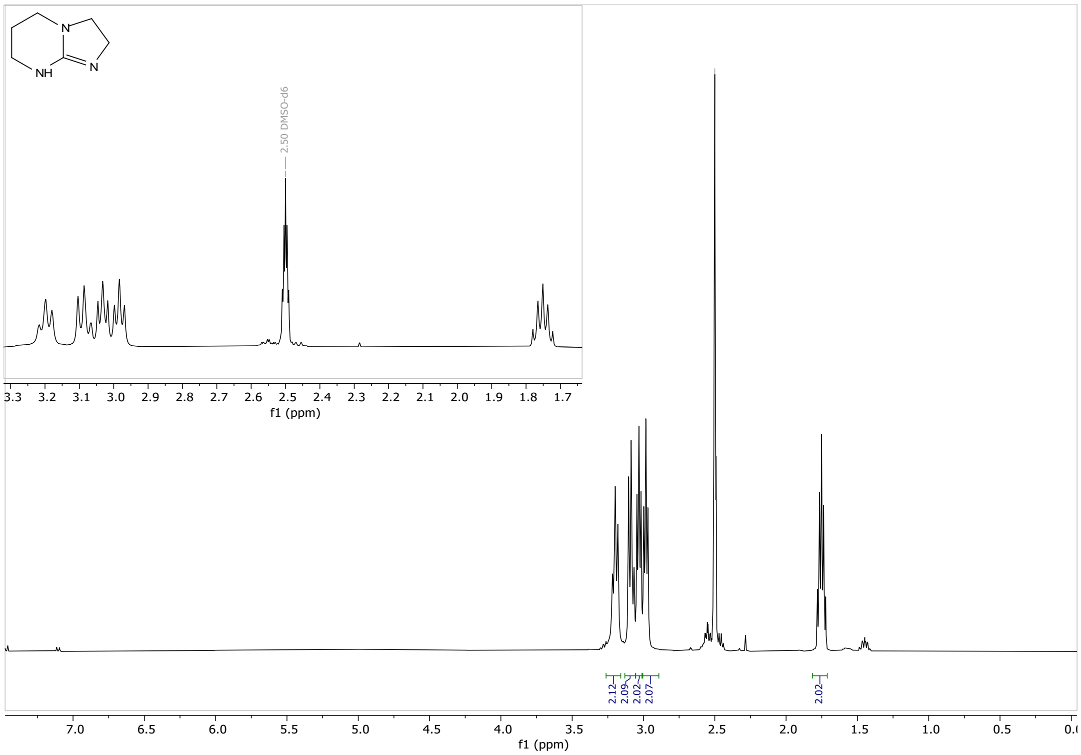

Figure S11: ^{1}H of **1,5,7-triazabicyclo[4.3.0]non-5-ene** in DMSO-d_6.

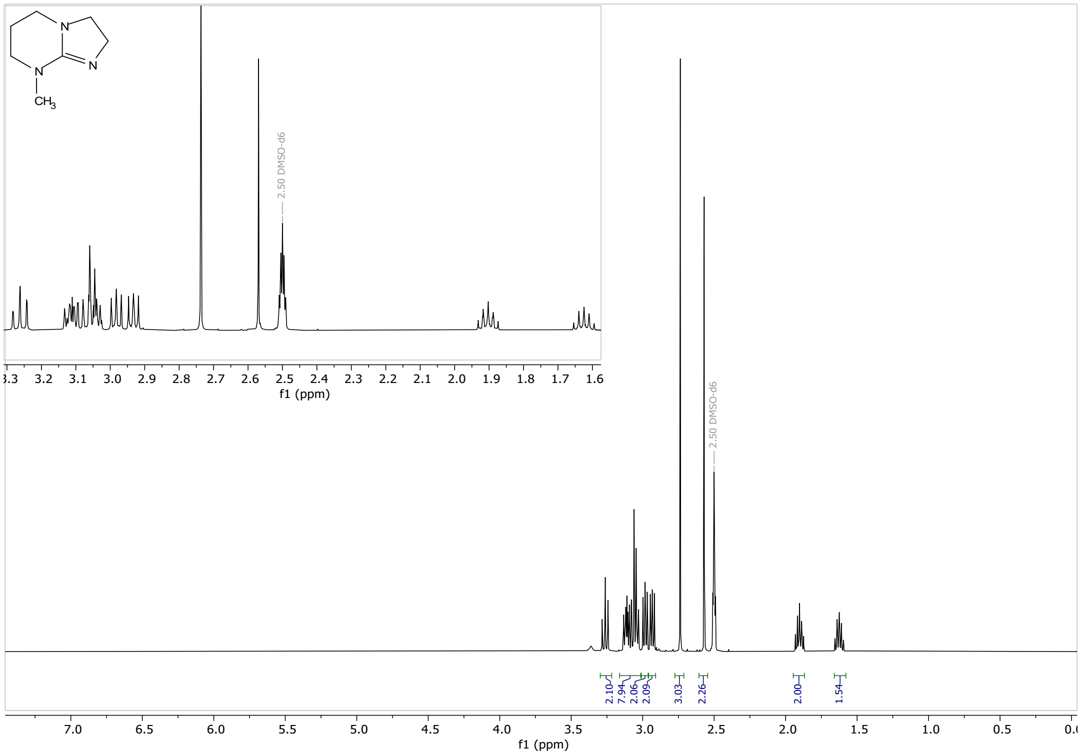

Figure S12: ^{1}H of **5-methyl-1,5,7-triazabicyclo[4.3.0]non-6-ene** in DMSO-d_6.

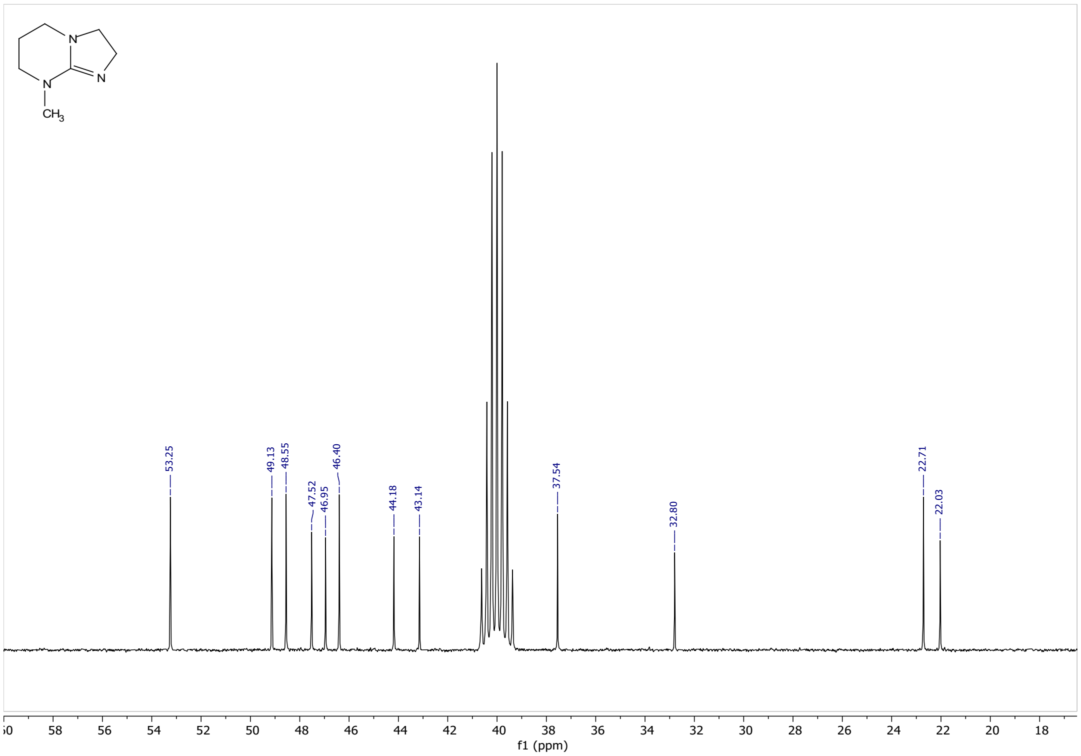

Figure S13: ^{13}C of **5-methyl-1,5,7-triazabicyclo[4.3.0]non-6-ene** in DMSO-d_6.

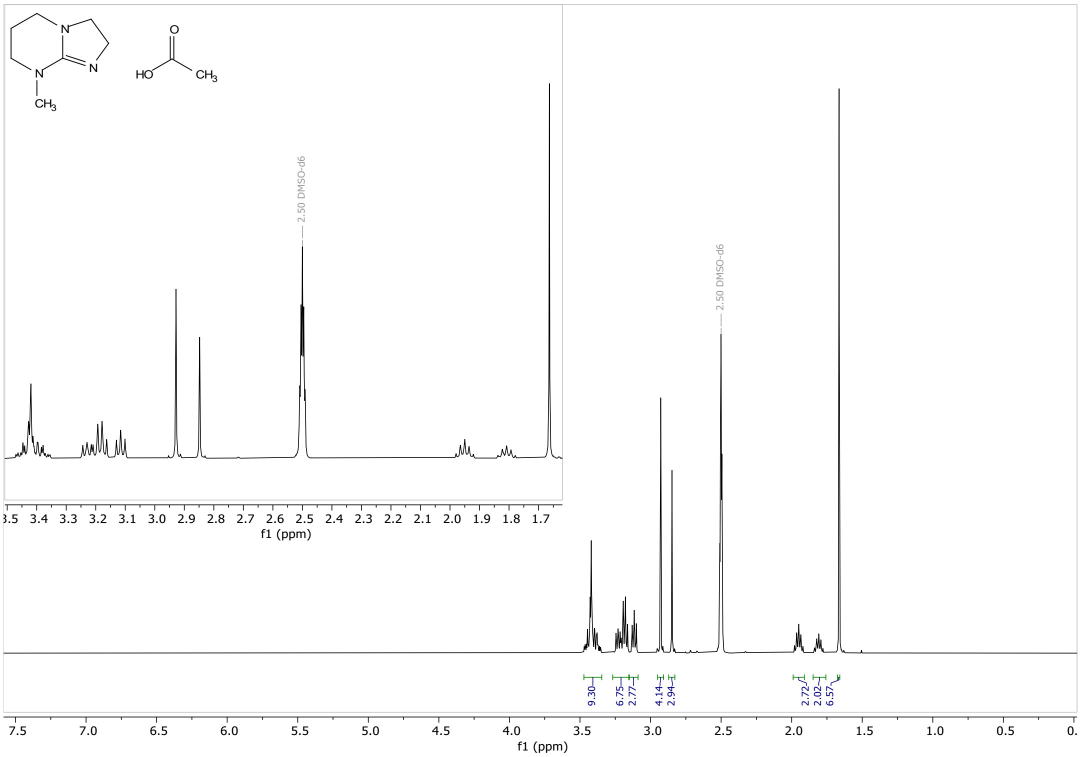

Figure S14: [1]H of **5-methyl-1,5,7-triazabicyclo[4.3.0]non-6-enium Acetate** in DMSO-d_6.

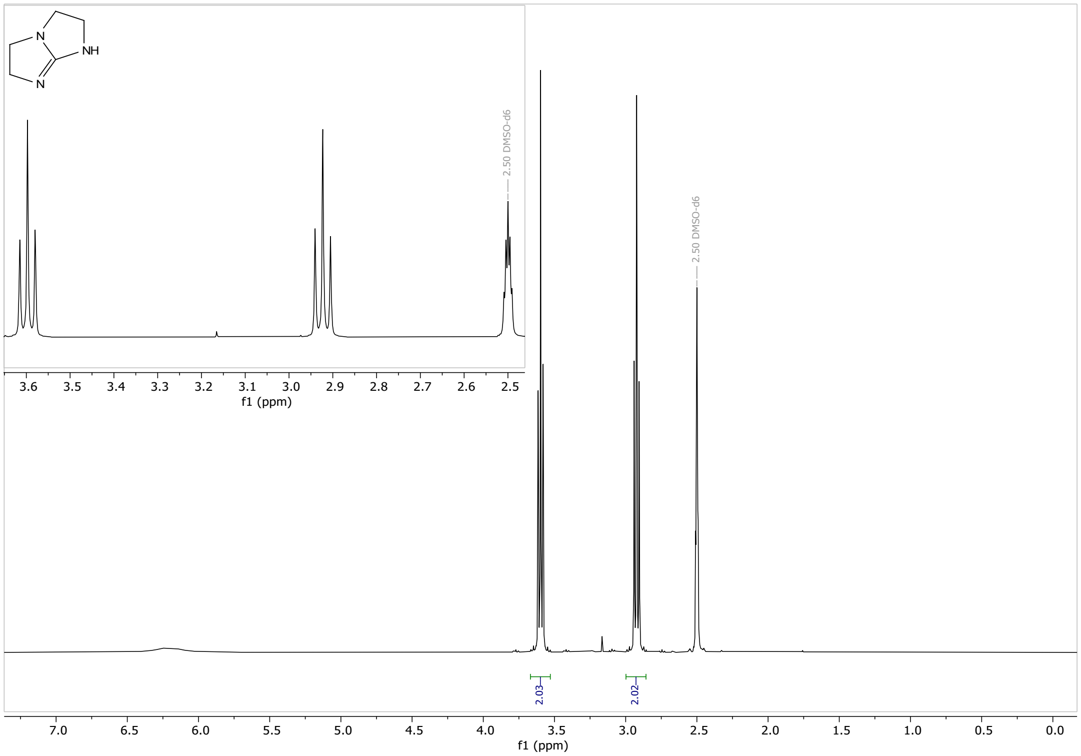

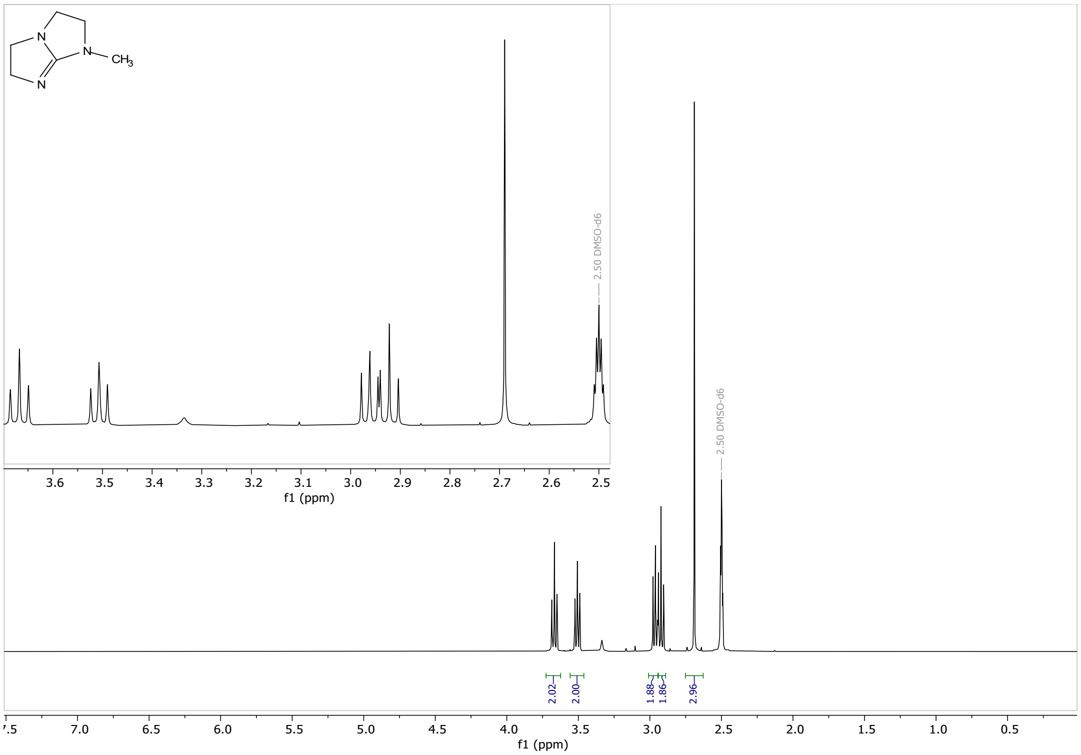

Figure S15: ^{1}H of **1,4,6-triazabicyclo[3.3.0]oct-4-ene** in DMSO-d_6.

Figure S16: ^{1}H of **4-methyl-1,4,6-triazabicyclo[3.3.0]oct-5-ene** in DMSO-d_6.

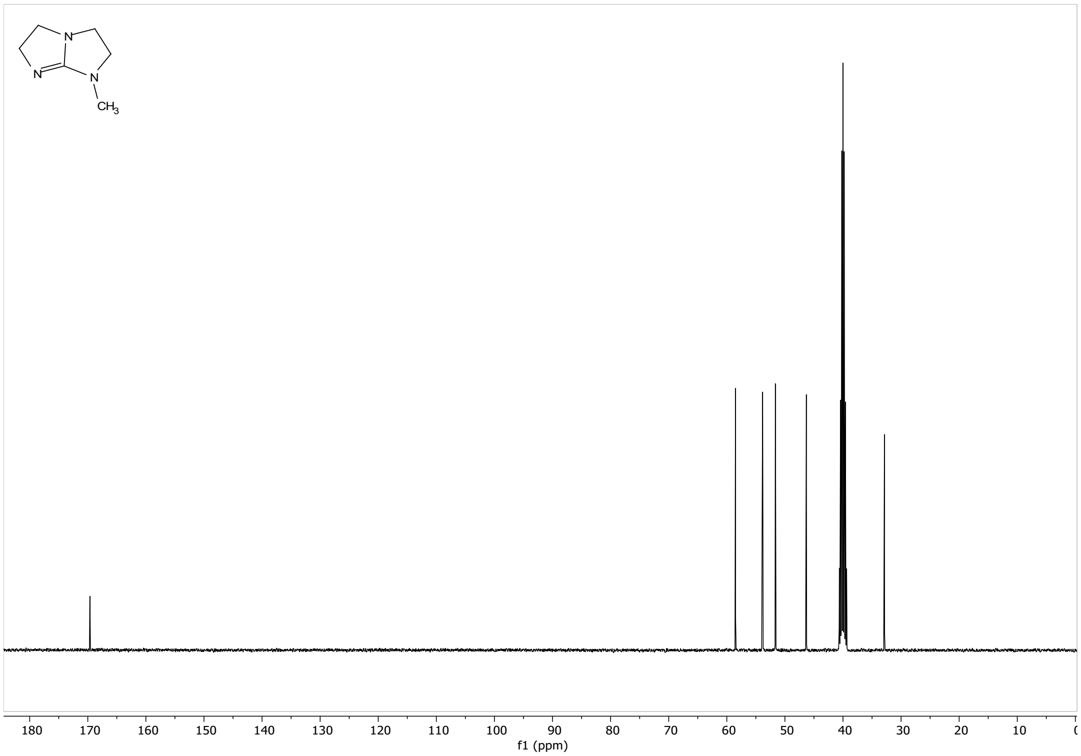

Figure S17: ^{13}C of **4-methyl-1,4,6-triazabicyclo[3.3.0]oct-5-ene** in DMSO-d_6

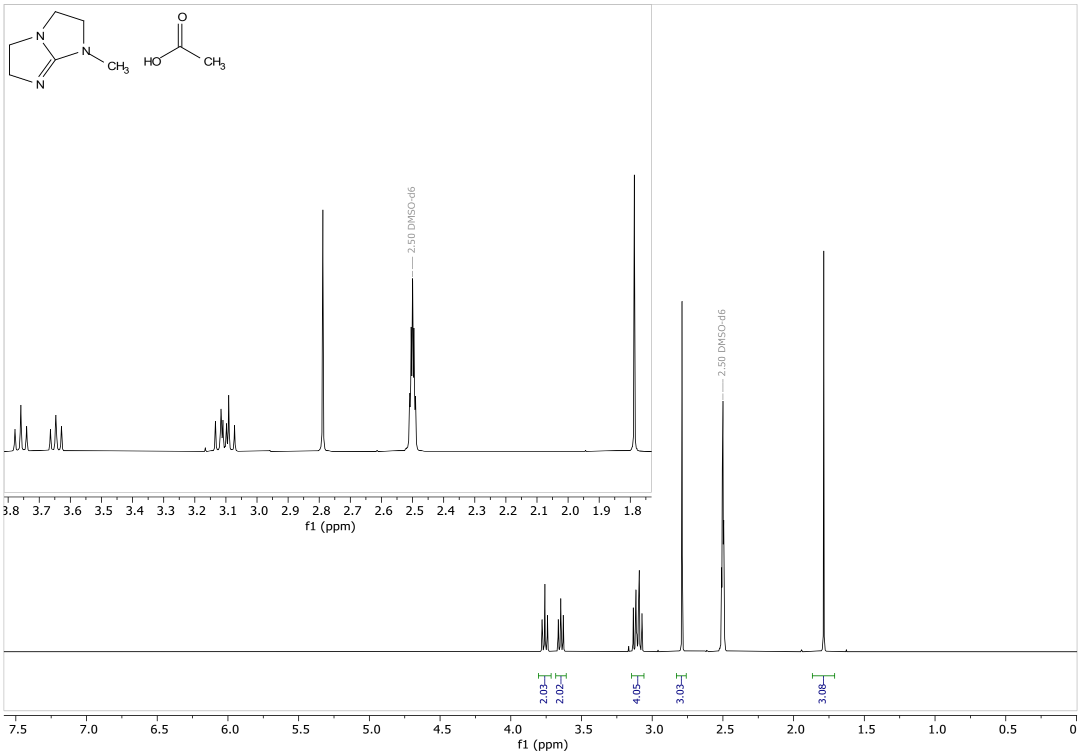

Figure S18: ^{1}H of **4-methyl-1,4,6-triazabicyclo[3.3.0]oct-5-enium Acetate** in DMSO-d_6.

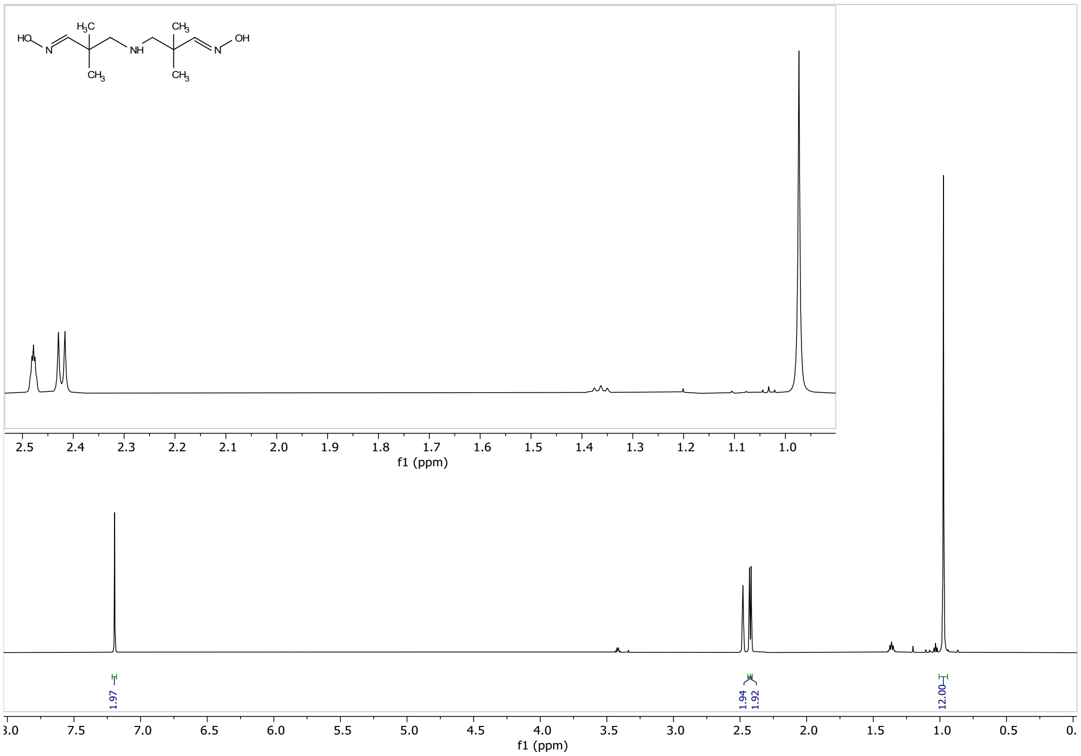

Figure S19: ^{1}H of **3,3'-Iminobis[2,2-dimethylpropanal] 1,1'-dioxime** in DMSO-d$_6$.

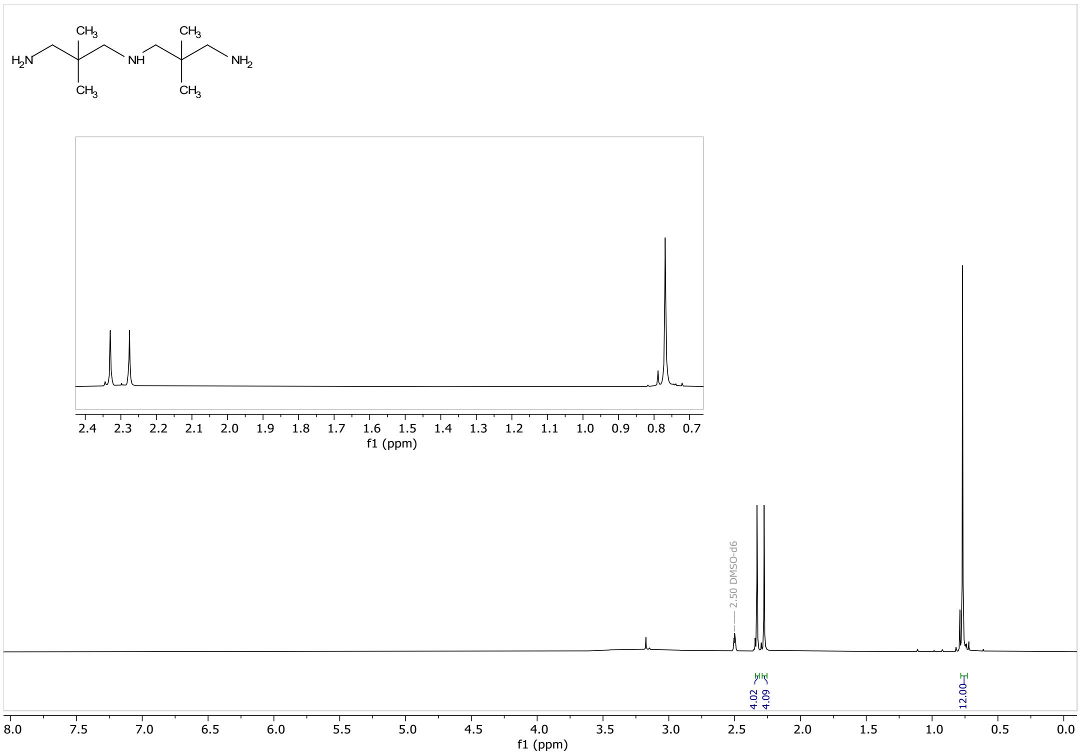

Figure S20: ^{1}H of **N1-(3-amino-2,2-dimethylpropyl)-2,2-dimethylpropane-1,3-diamine** in DMSO-d$_6$.

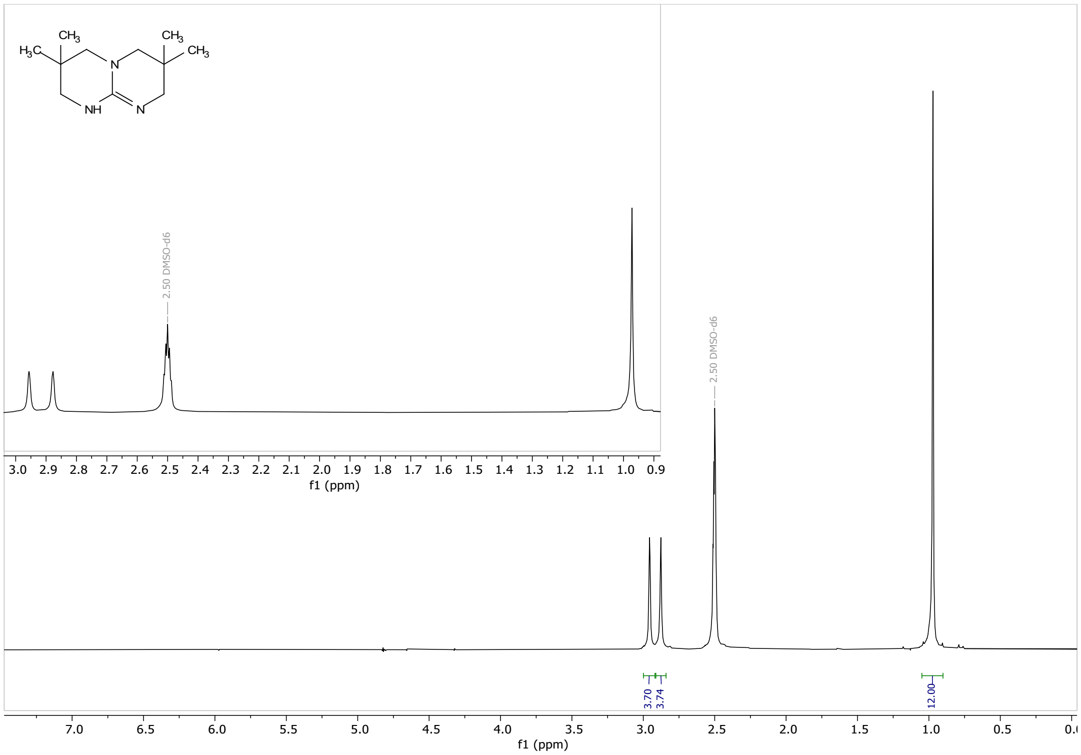

Figure S21: ^{1}H of **3,3-dimethyl-9,9-dimethyl-1,5,7-Triazabicyclo[4.4.0]dec-5-ene** in DMSO-d$_6$.

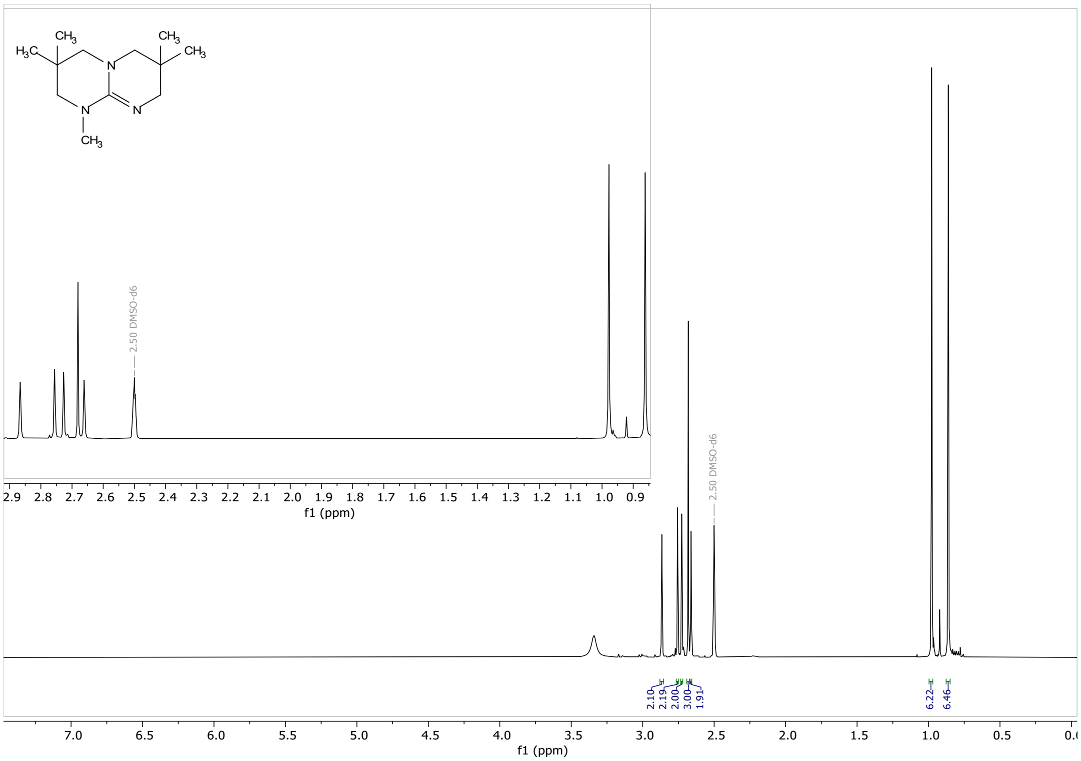

Figure S22: ^{1}H of **7-methyl-3,3-dimethyl-9,9-dimethyl-1,5,7-Triazabicyclo[4.4.0]dec-5-ene** in DMSO-d_6.

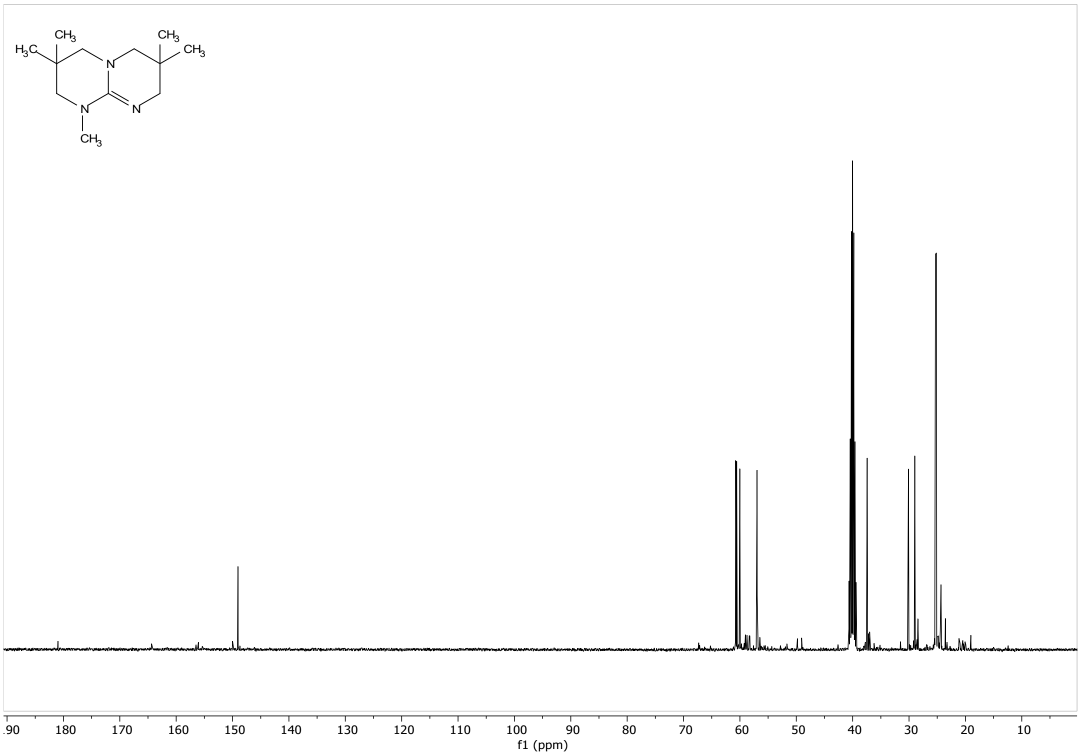

Figure S23: ^{13}C of **7-methyl-3,3-dimethyl-9,9-dimethyl-1,5,7-Triazabicyclo[4.4.0]dec-5-ene** in DMSO-d_6.

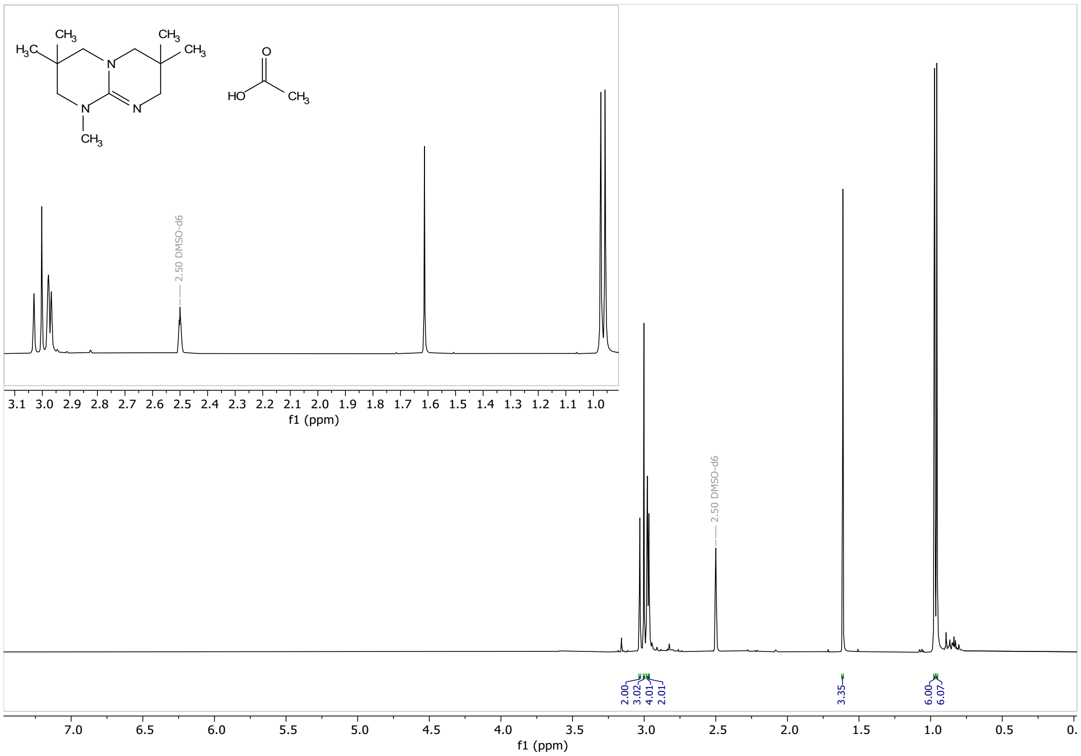

Figure S24: ^{1}H of **7-methyl-3,3-dimethyl-9,9-dimethyl-1,5,7-Triazabicyclo[4.4.0]dec-5-enium Acetate** in DMSO-d_6.

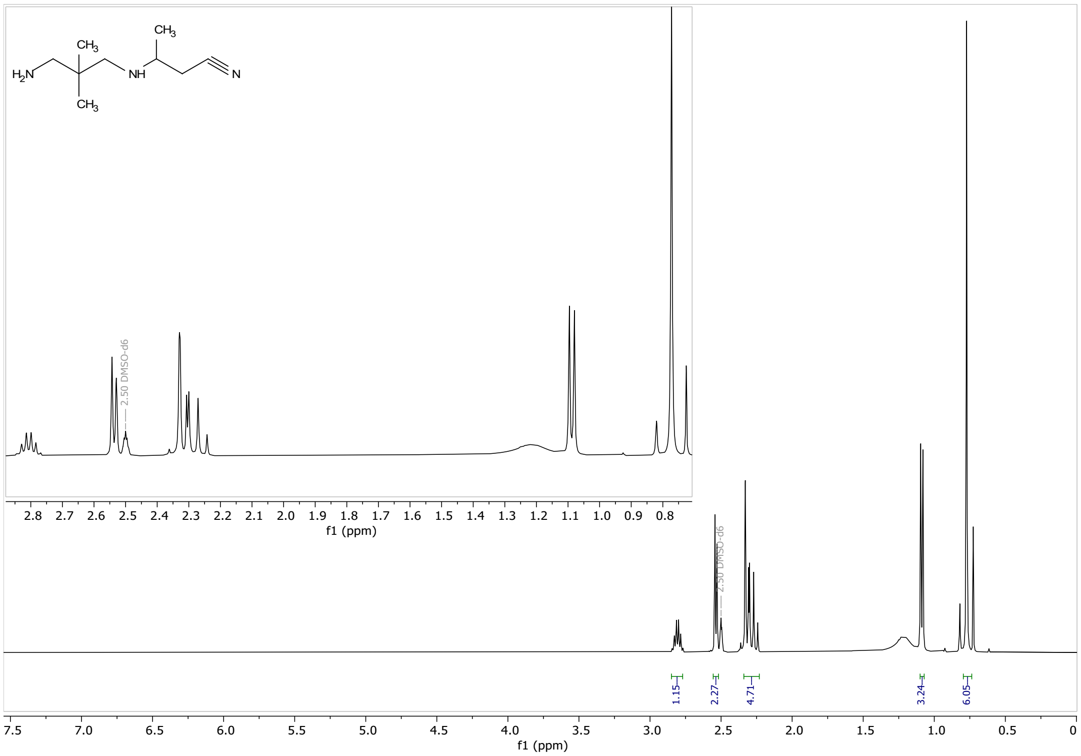

Figure S25: ^{1}H of **3-((3-amino-2,2-dimethylpropyl)amino)butanenitrile** in DMSO-d_6.

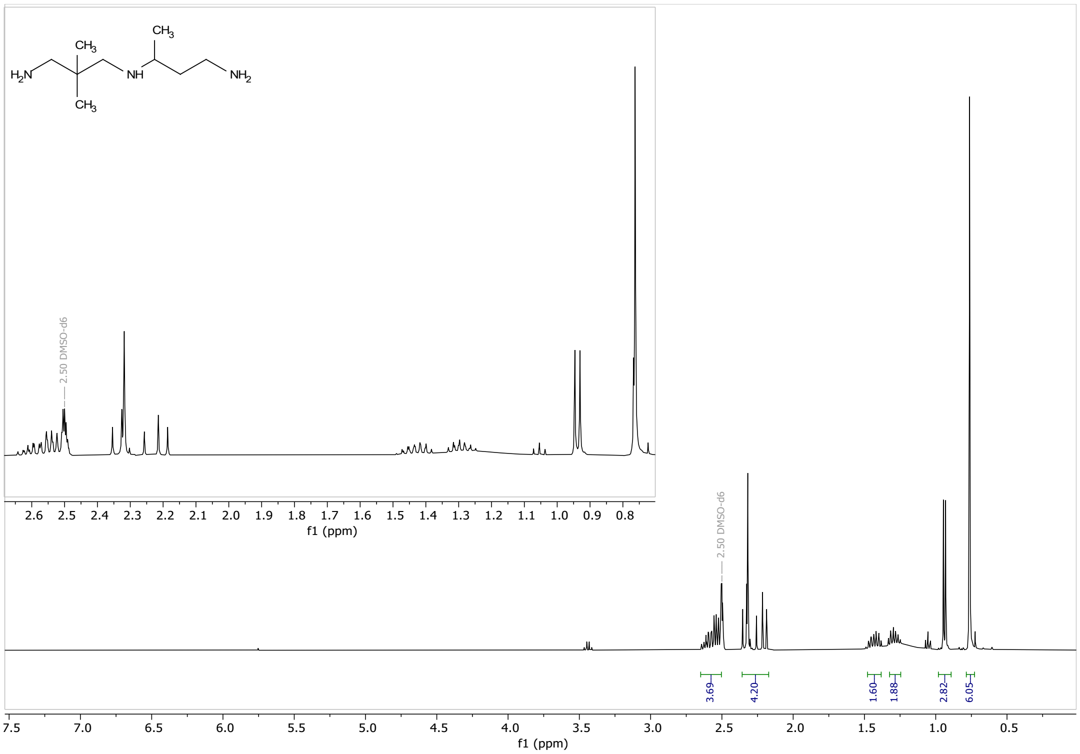

Figure S26: ^{1}H of **N3-(3-amino-2,2-dimethylpropyl)butane-1,3-diamine** in DMSO-d_6.

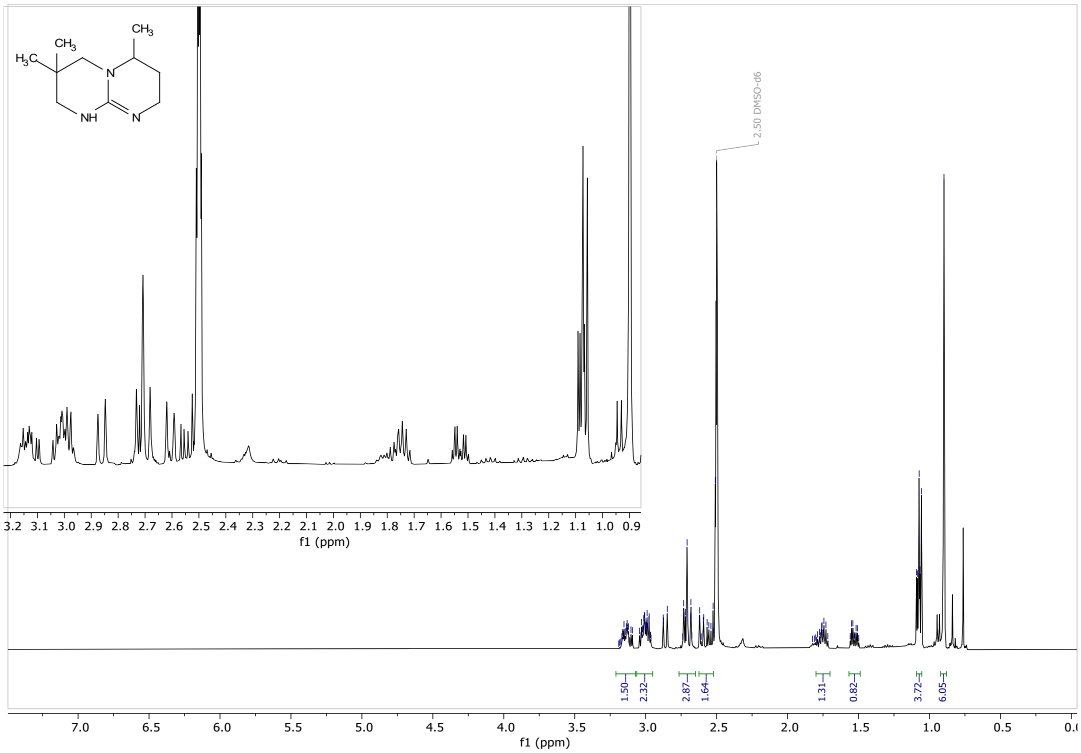

Figure S27: ^{1}H of **3,3-dimethyl-10-methyl-1,5,7-Triazabicyclo[4.4.0]dec-5-ene** in DMSO-d_6.

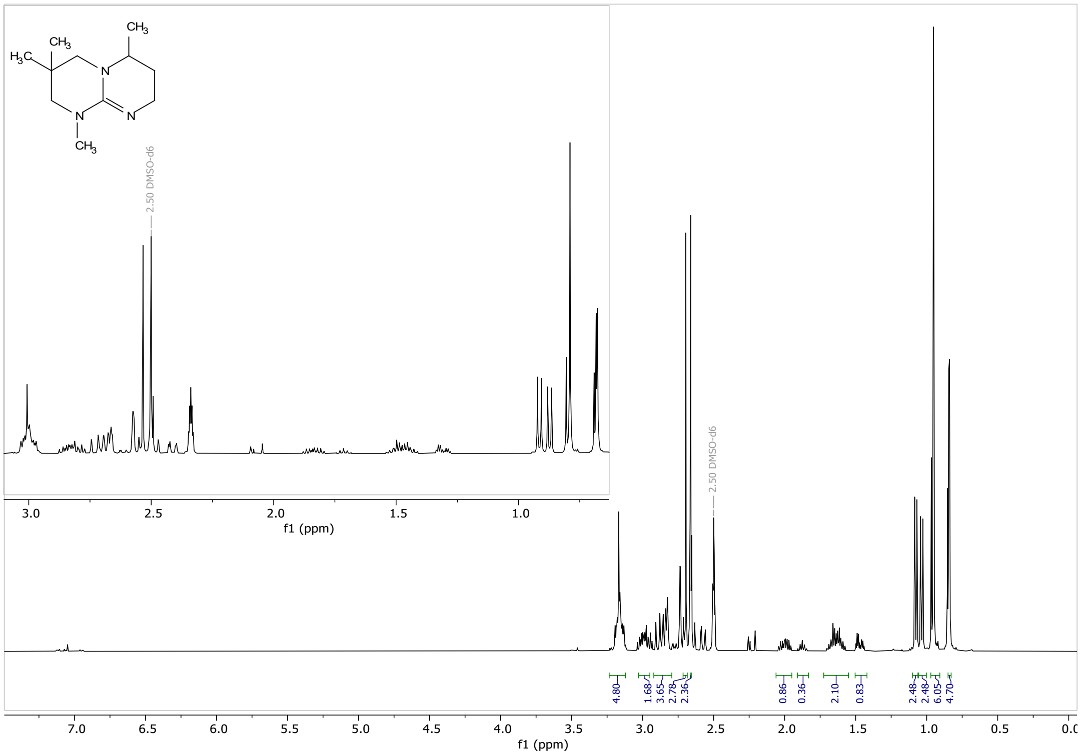

Figure S28: ^{1}H of **7-methyl-3,3-dimethyl-10-methyl-1,5,7-Triazabicyclo[4.4.0]dec-5-ene** in DMSO-d_6.

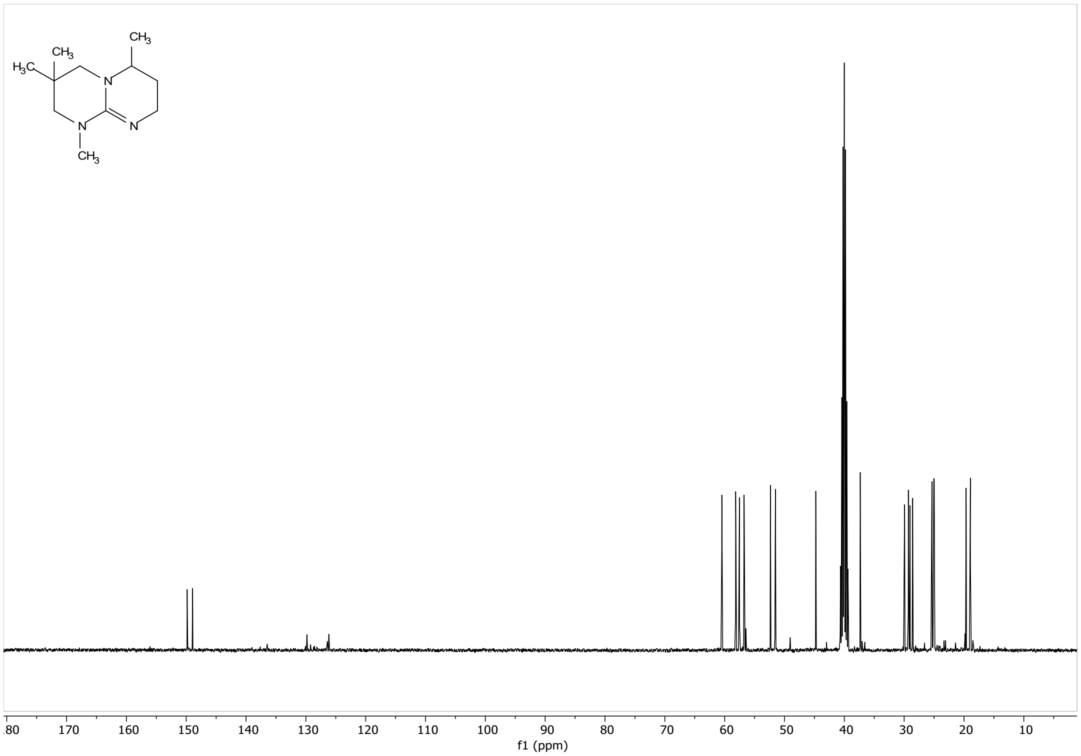

Figure S29: ^{13}C of **7-methyl-3,3-dimethyl-10-methyl-1,5,7-Triazabicyclo[4.4.0]dec-5-ene** in DMSO-d_6.

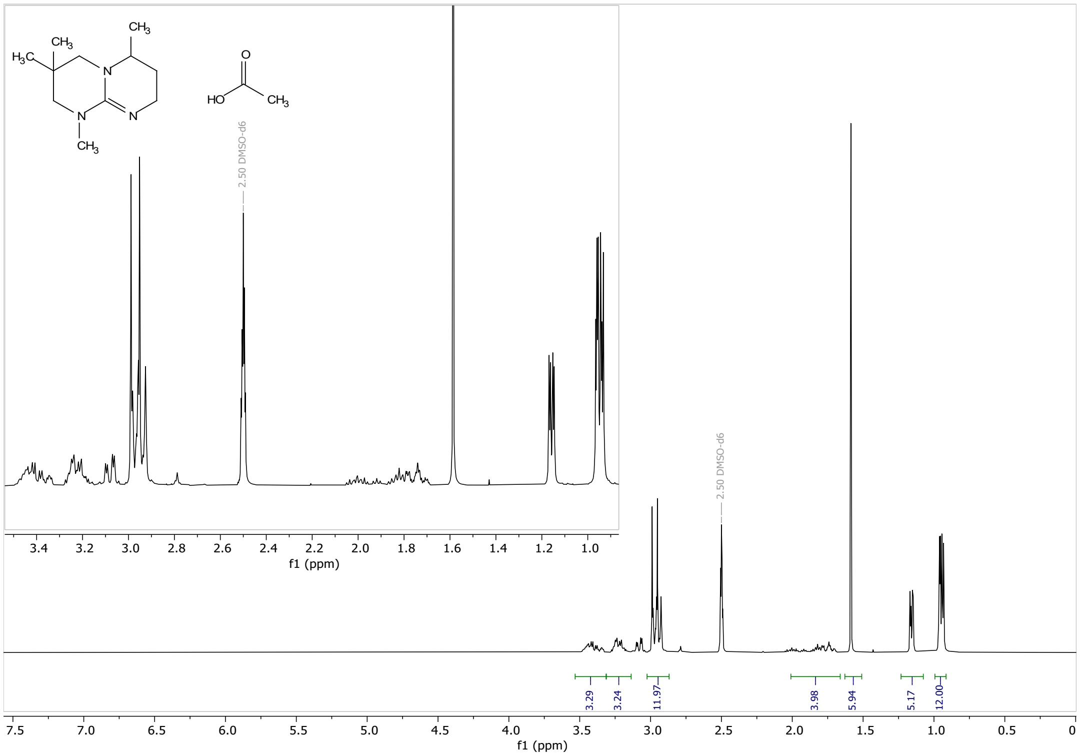

Figure S30: ^{1}H of **7-methyl-3,3-dimethyl-10-methyl-1,5,7-Triazabicyclo[4.4.0]dec-5-enium Acetate** in DMSO-d_6.

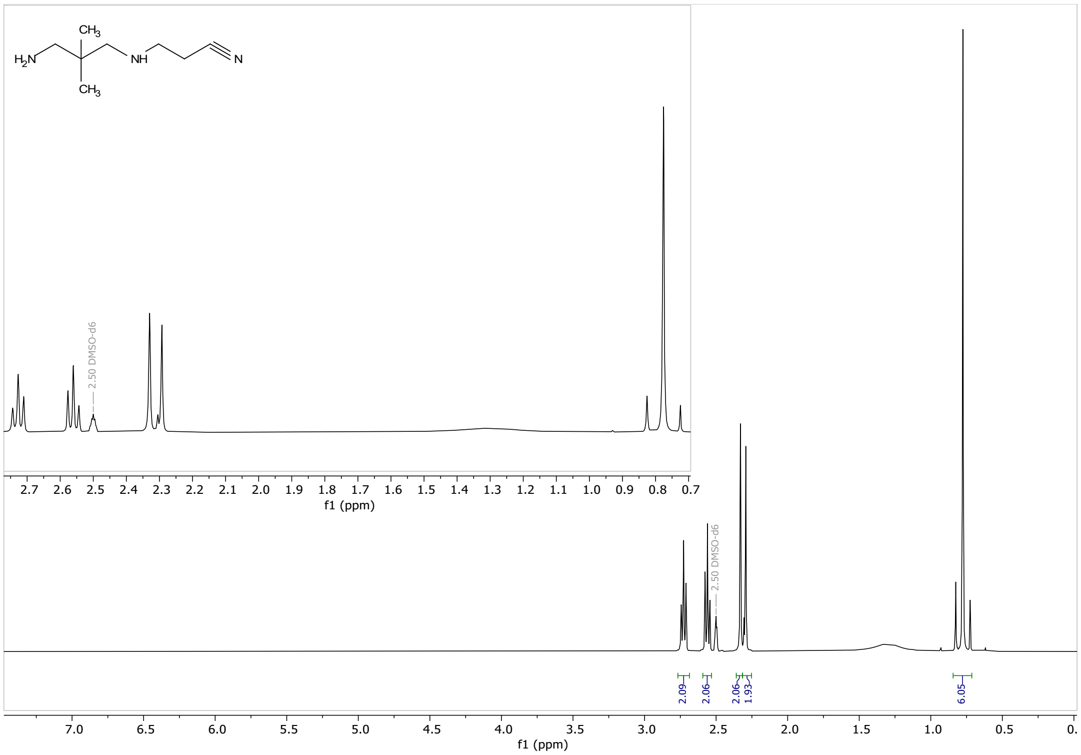

Figure S31: ^{1}H of **3-((3-amino-2,2-dimethylpropyl)amino)propanenitrile** in DMSO-d_6.

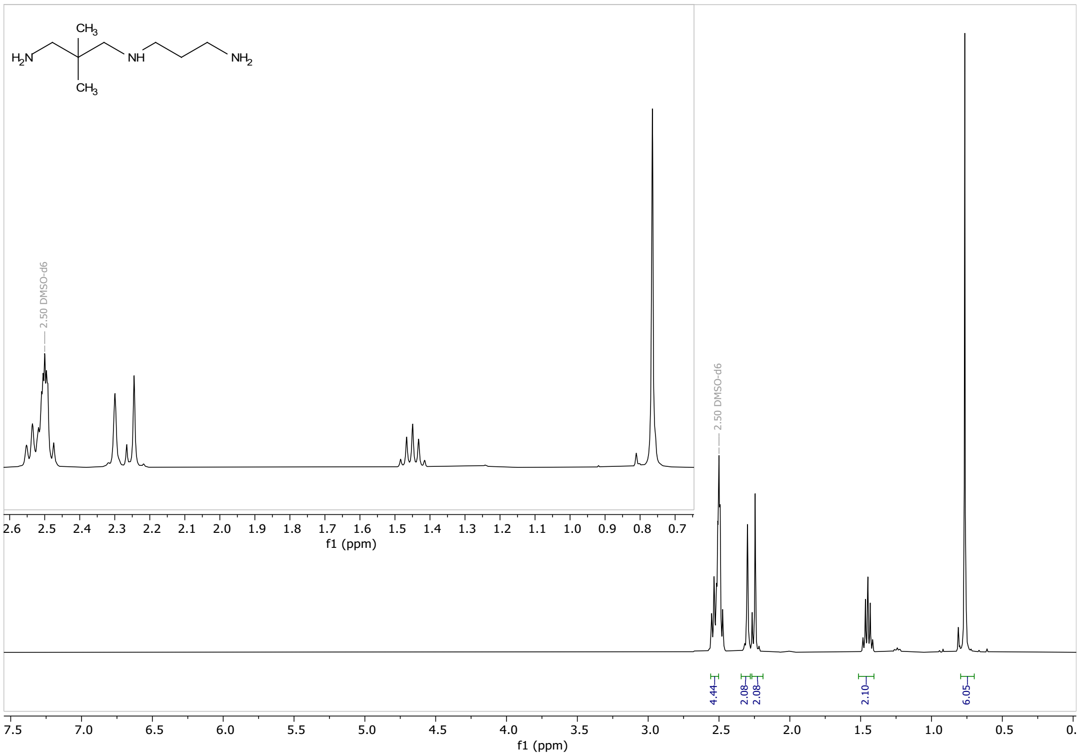

Figure S32: ^{1}H of **Synthesis of N1-(3-aminopropyl)-2,2-dimethylpropane-1,3-diamine** in DMSO-d_6.

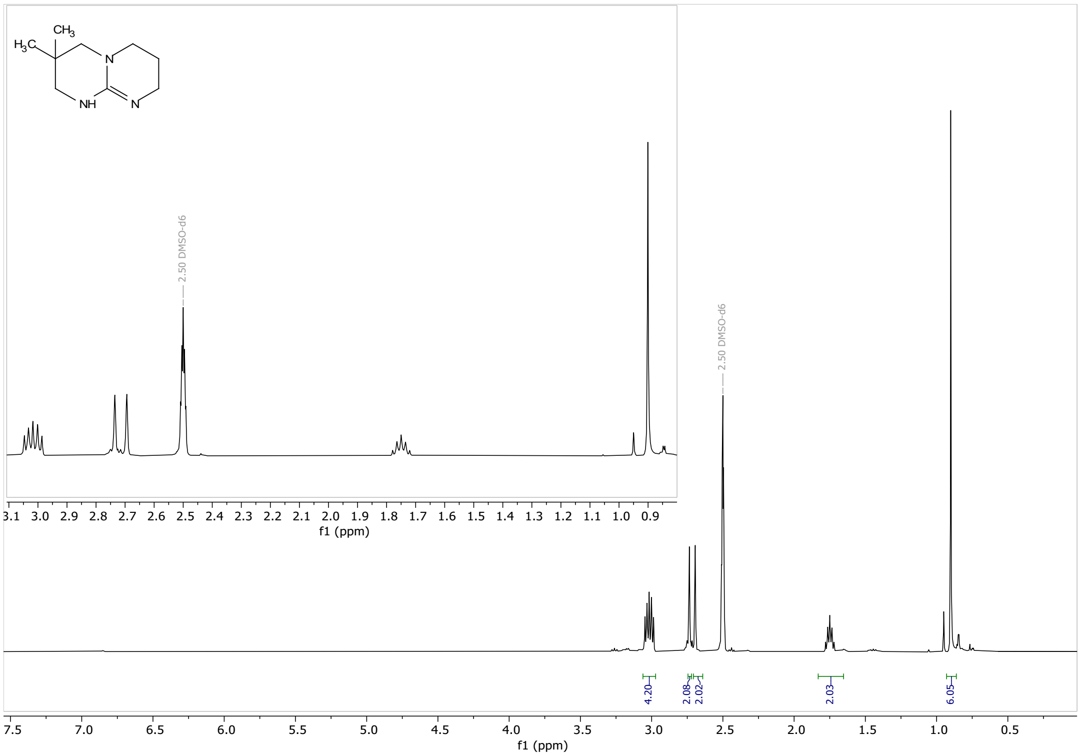

Figure S33: ^{1}H of **3,3-dimethyl-1,5,7-Triazabicyclo[4.4.0]dec-5-ene** in DMSO-d_6.

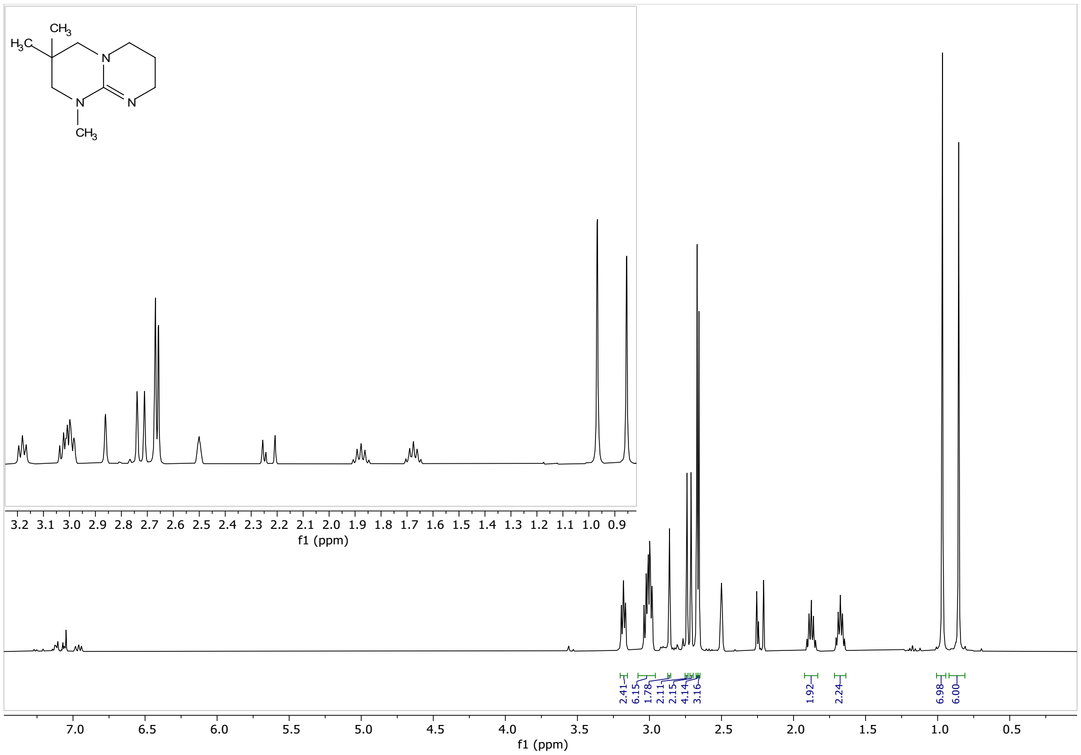

Figure S34: [1]H of **7-methyl-3,3-dimethyl-1,5,7-Triazabicyclo[4.4.0]dec-5-ene** in DMSO-d_6.

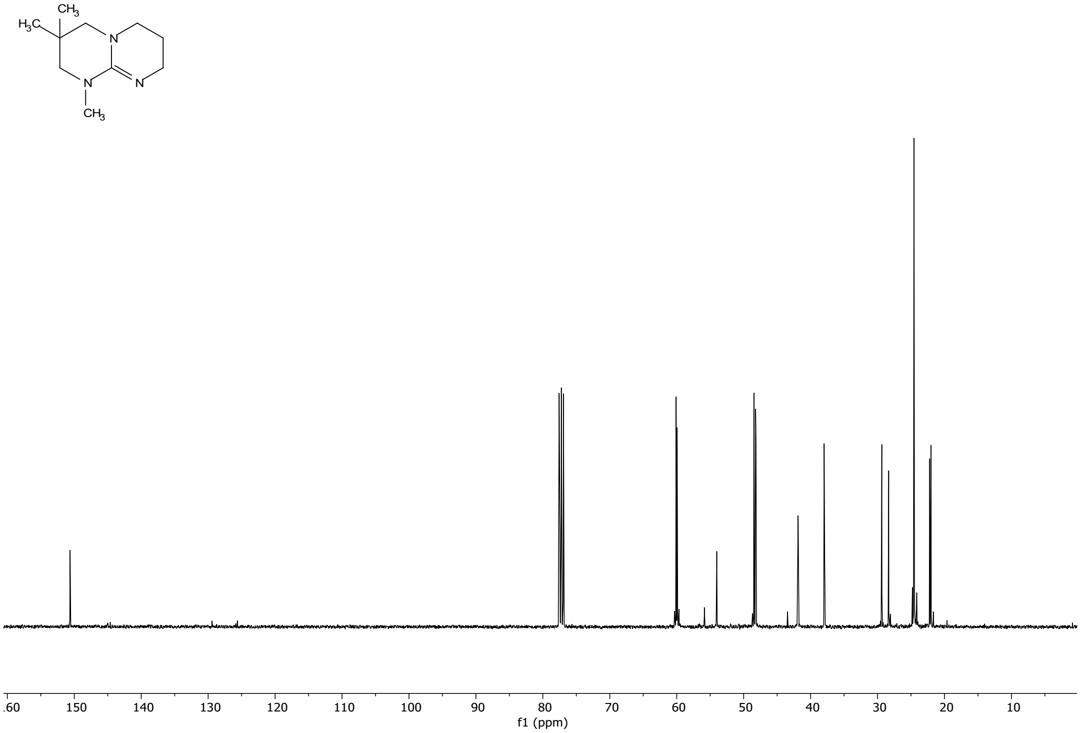

Figure S35: [13]C of **7-methyl-3,3-dimethyl-1,5,7-Triazabicyclo[4.4.0]dec-5-ene** in $CDCl_3$.

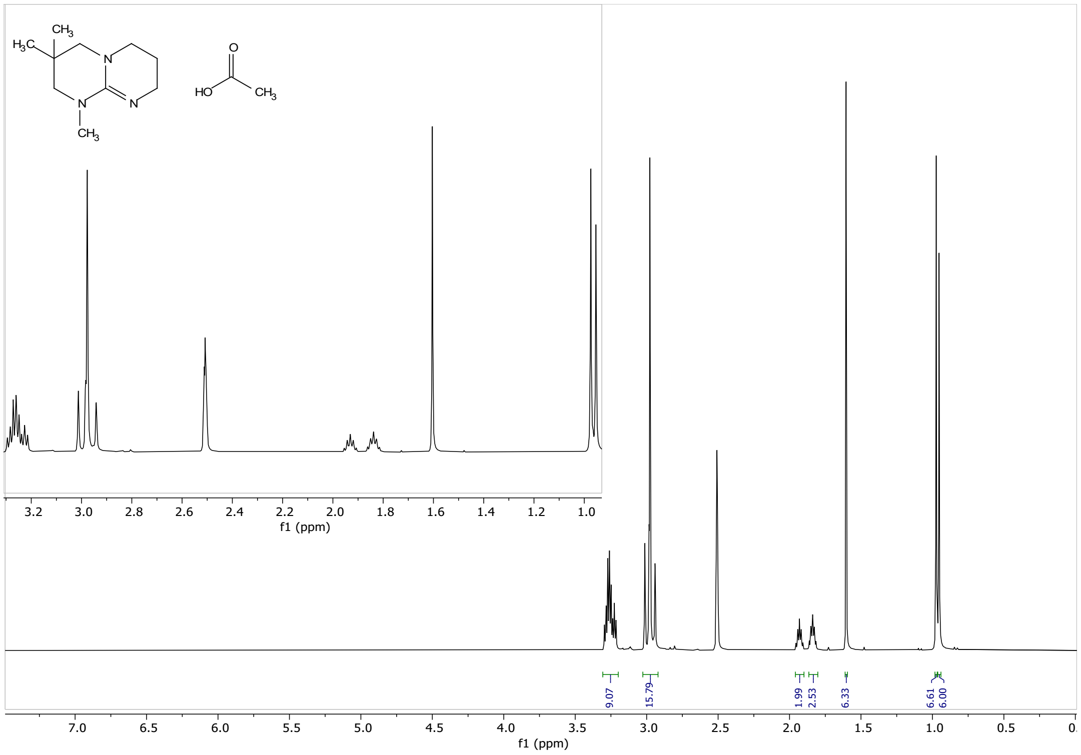

Figure S36: ^{1}H of **7-methyl-3,3-dimethyl-1,5,7-Triazabicyclo[4.4.0]dec-5-enium Acetate** in DMSO-d_6.

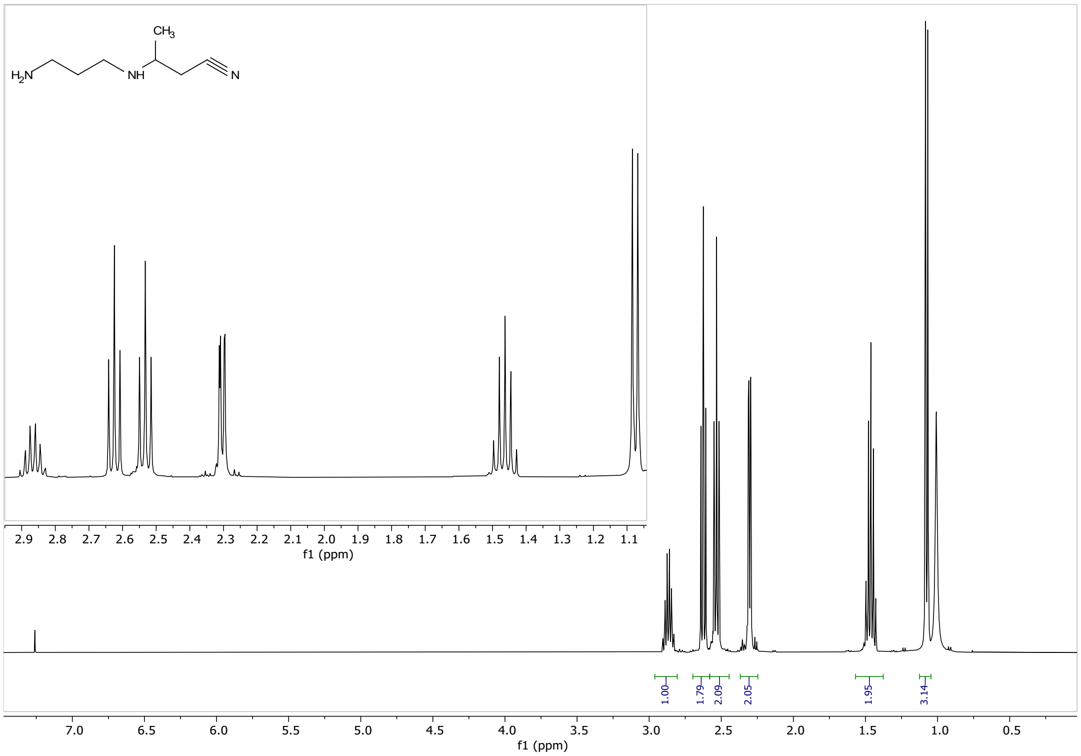

Figure S37: ^{1}H of **3-((3-aminopropyl)amino)butanenitrile** in DMSO-d_6.

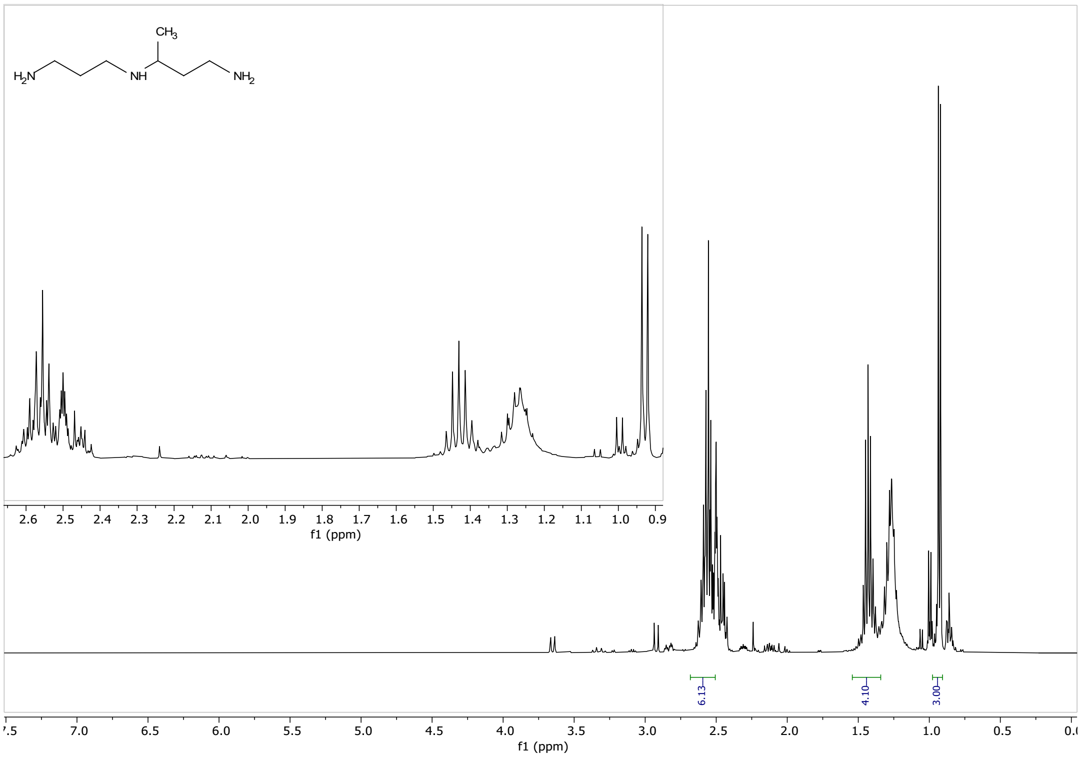

Figure S38: ^{1}H of **N3-(3-aminopropyl)butane-1,3-diamine** in DMSO-d$_6$.

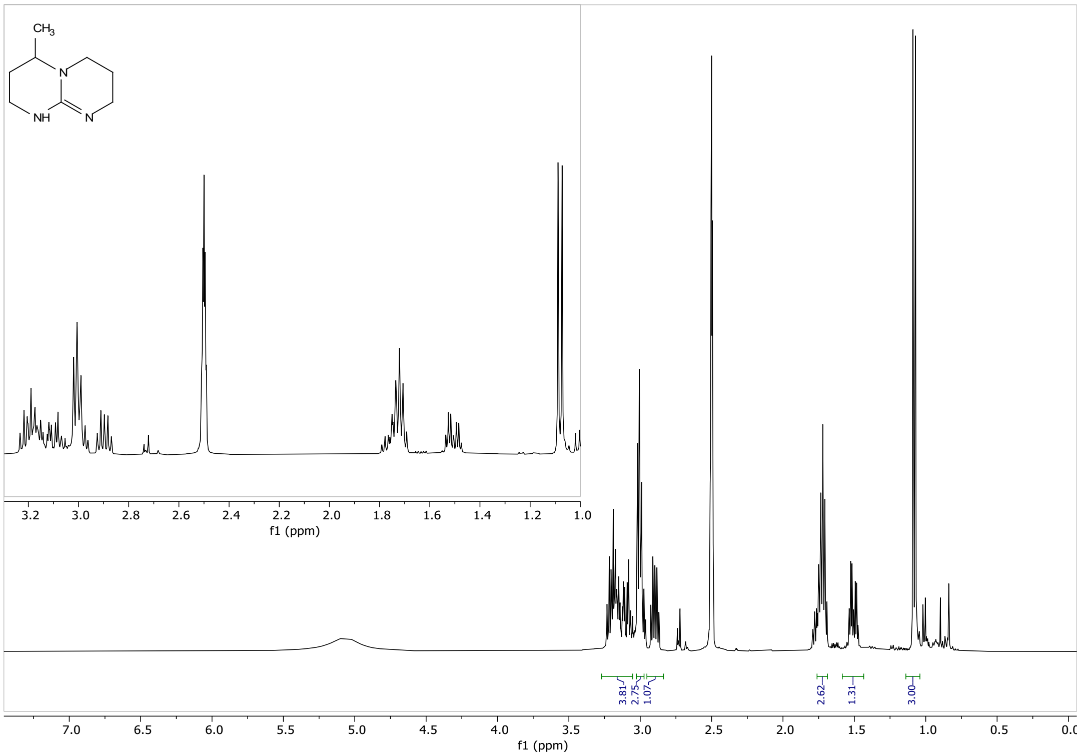

Figure S39: ^{1}H of **2-methyl-1,5,7-Triazabicyclo[4.4.0]dec-5-ene** in DMSO-d$_6$.

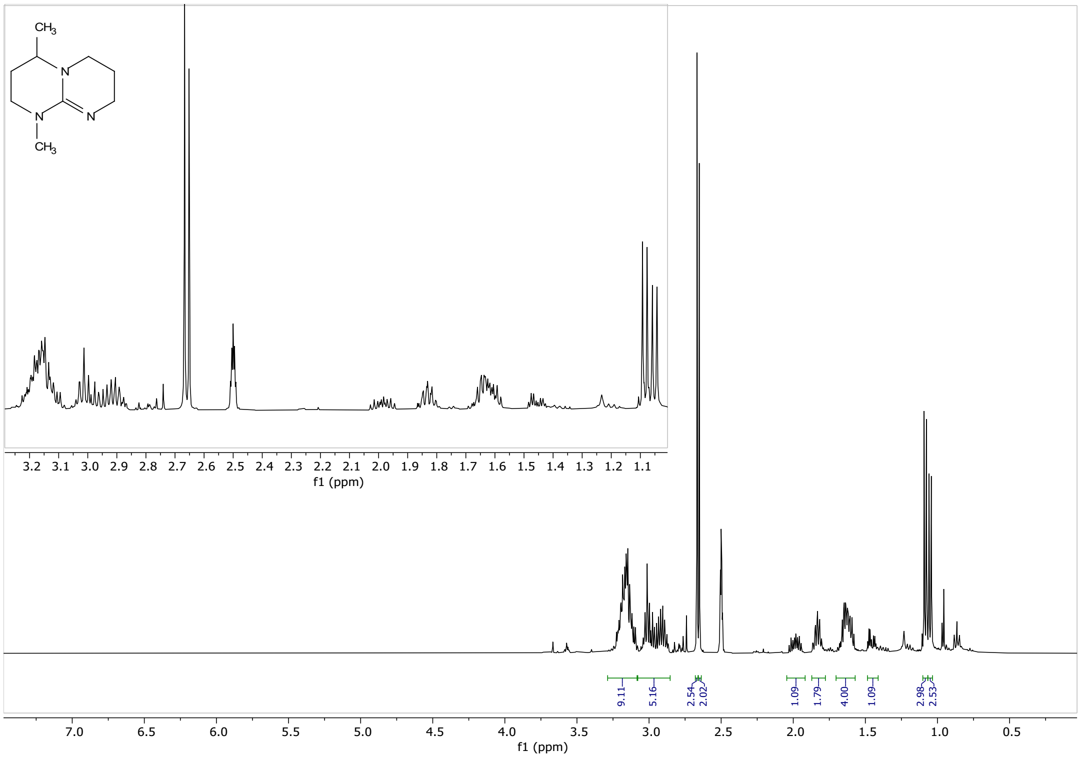

Figure S40: ^{1}H of **7-methyl-2-methyl-1,5,7-Triazabicyclo[4.4.0]dec-5-ene** in DMSO-d_6.

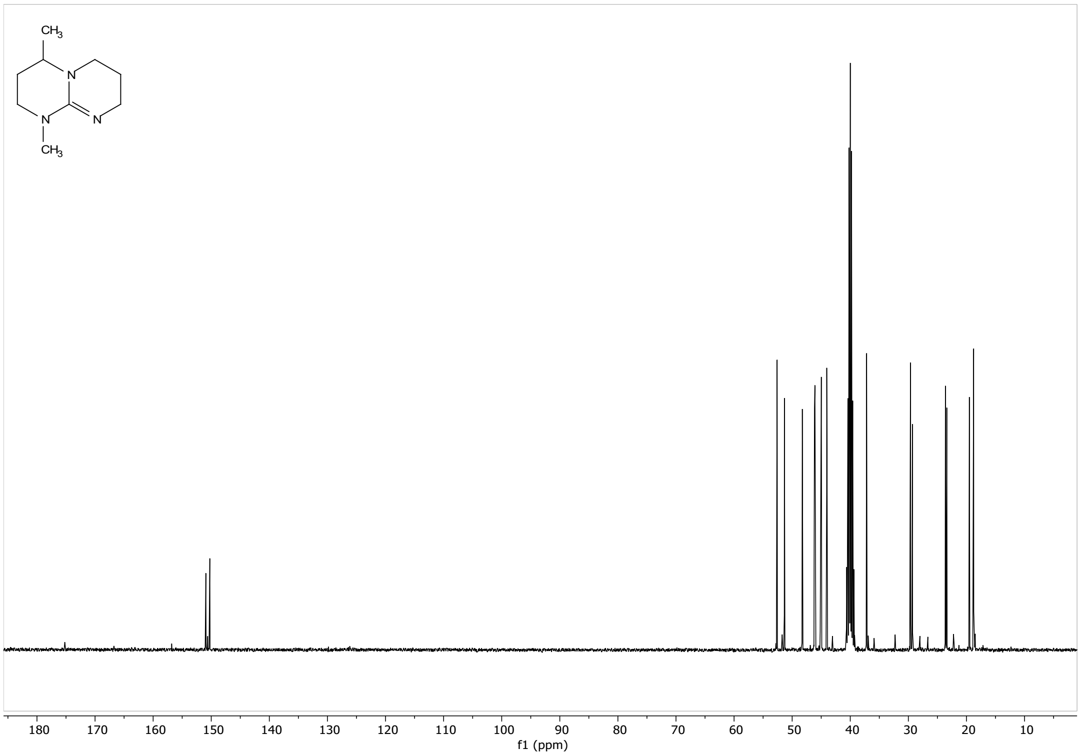

Figure S41: ^{13}C of **7-methyl-2-methyl-1,5,7-Triazabicyclo[4.4.0]dec-5-ene** in DMSO-d_6.

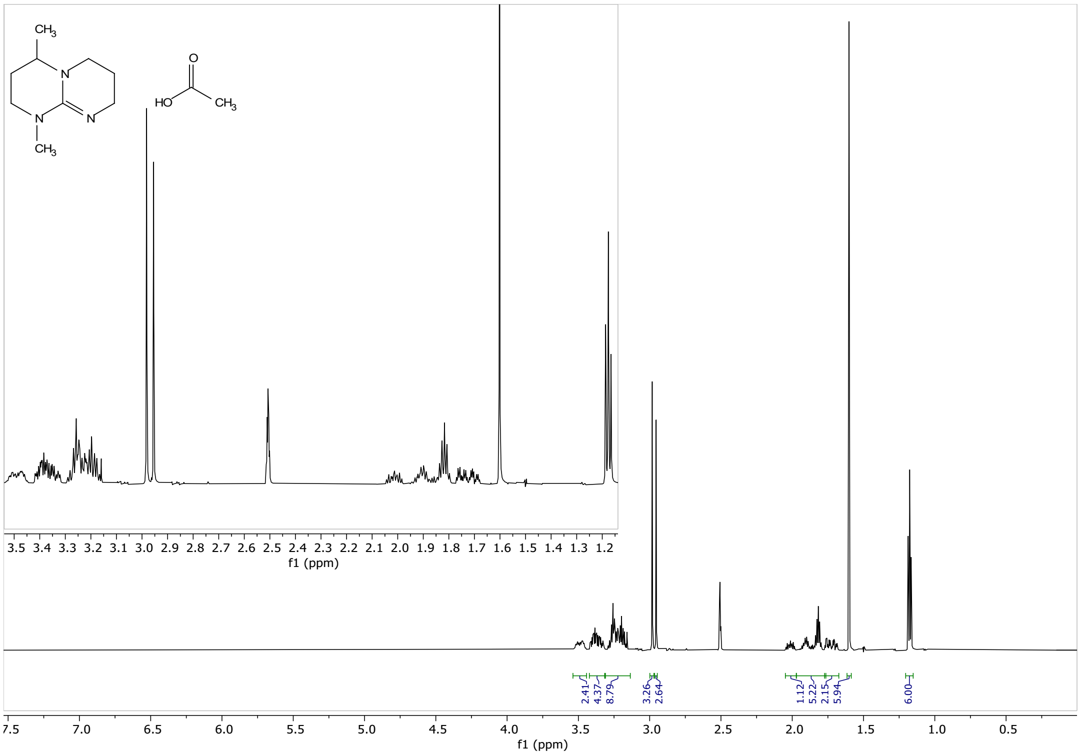

Figure S42: ^{1}H of **7-methyl-2-methyl-1,5,7-Triazabicyclo[4.4.0]dec-5-enium Acetate** in DMSO-d_6.

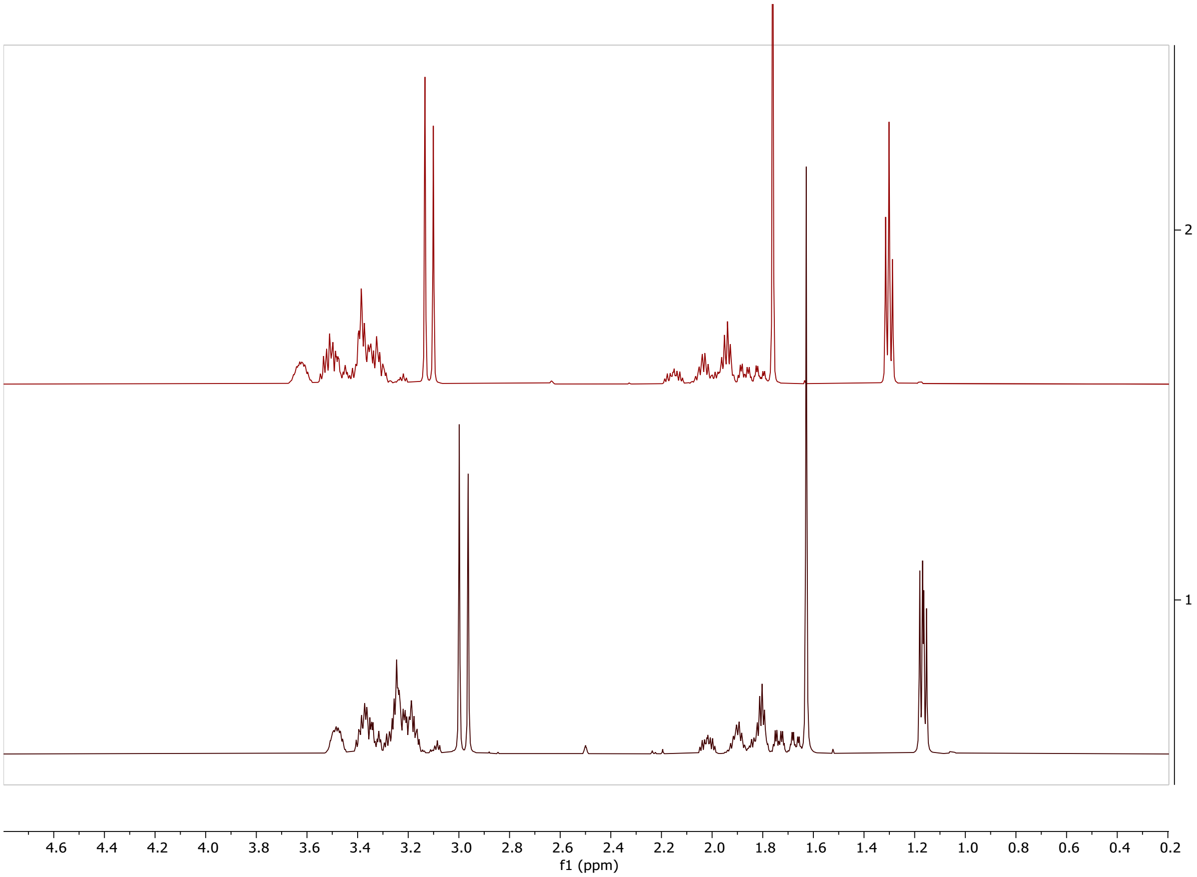

Figure S43: ^{1}H of **7-methyl-2-methyl-1,5,7-Triazabicyclo[4.4.0]dec-5-enium Acetate** and Birch kraft pulp in DMSO-d_6. Top spectrum was run the same day as the dissolution experiment. The down spectrum was run three months later.

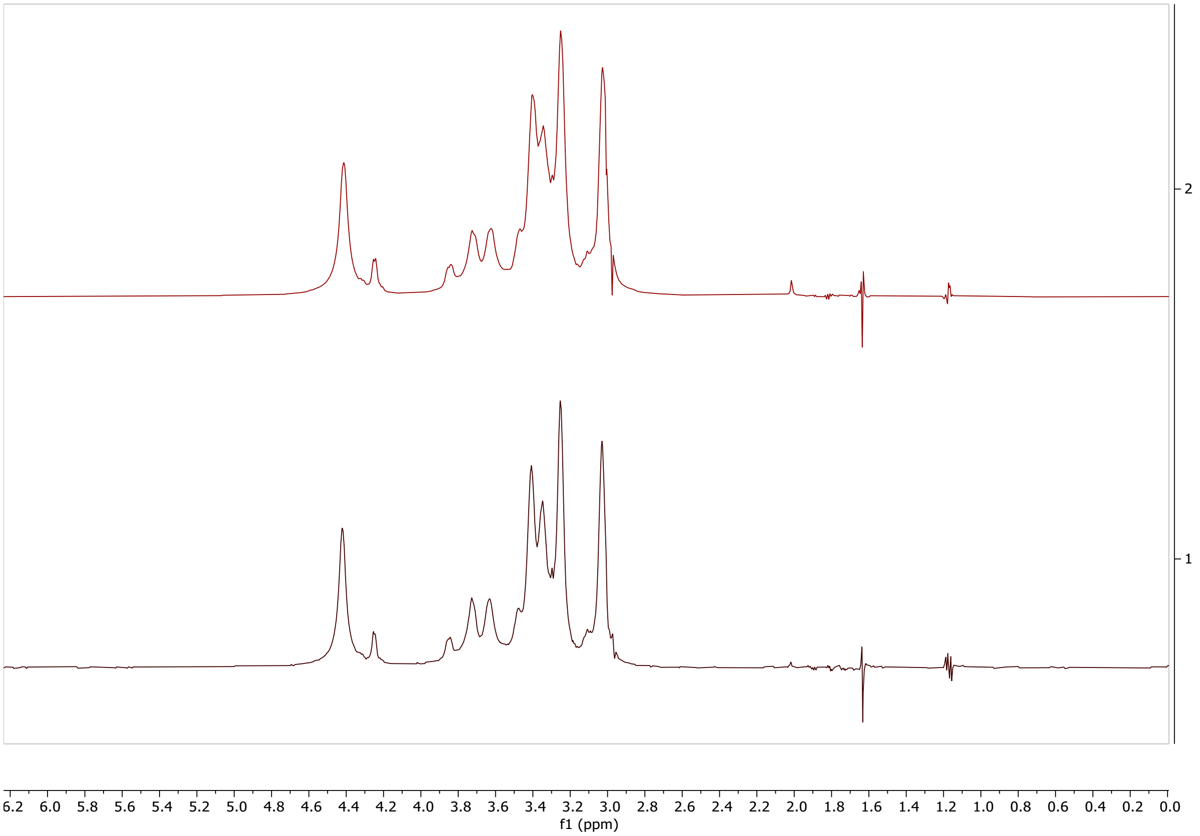

Figure S44: ^{1}H and diffusion-edited ^{1}H of birch kraft pulp dissolved in **7-methyl-2-methyl-1,5,7-Triazabicyclo[4.4.0]dec-5-enium Acetate** in DMSO-d_6. Top spectrum was run the same day as the dissolution experiment. The down spectrum was run three months later.

VI. Microscope images

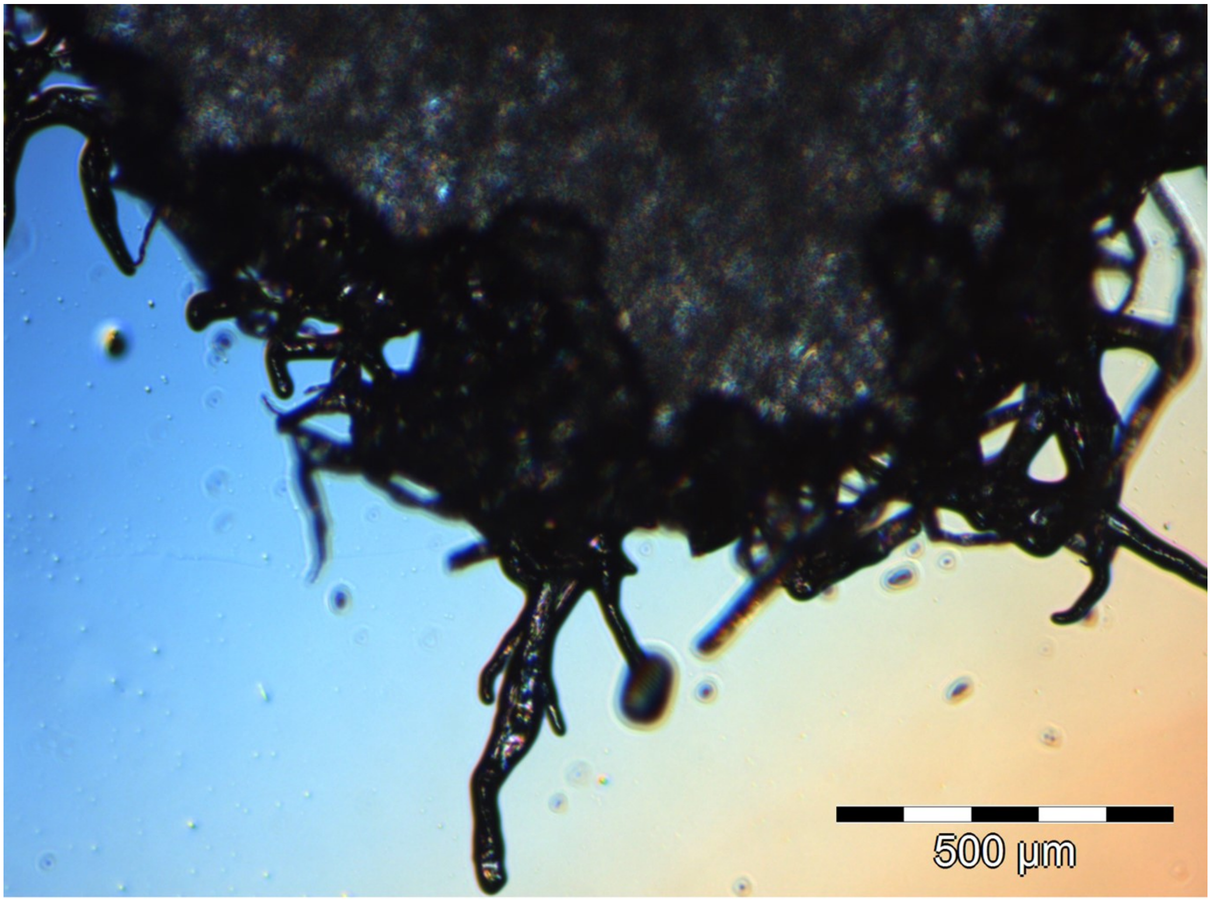

Figure S45: Kraft pulp fibres non dissolved in DMSO, this image is used as a reference material.

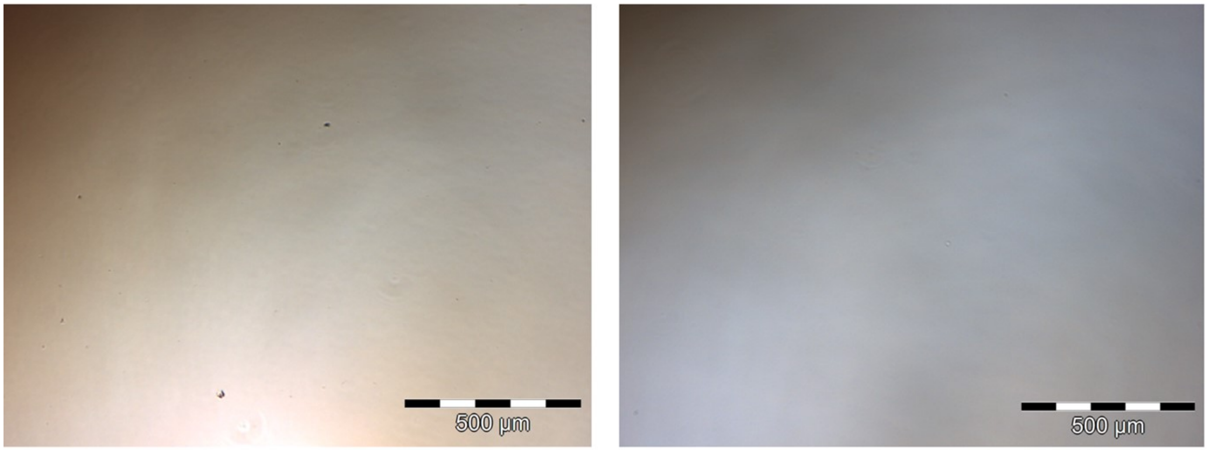

Figure S46: Enocell fibres dissolved in [mTBNH][OAc]

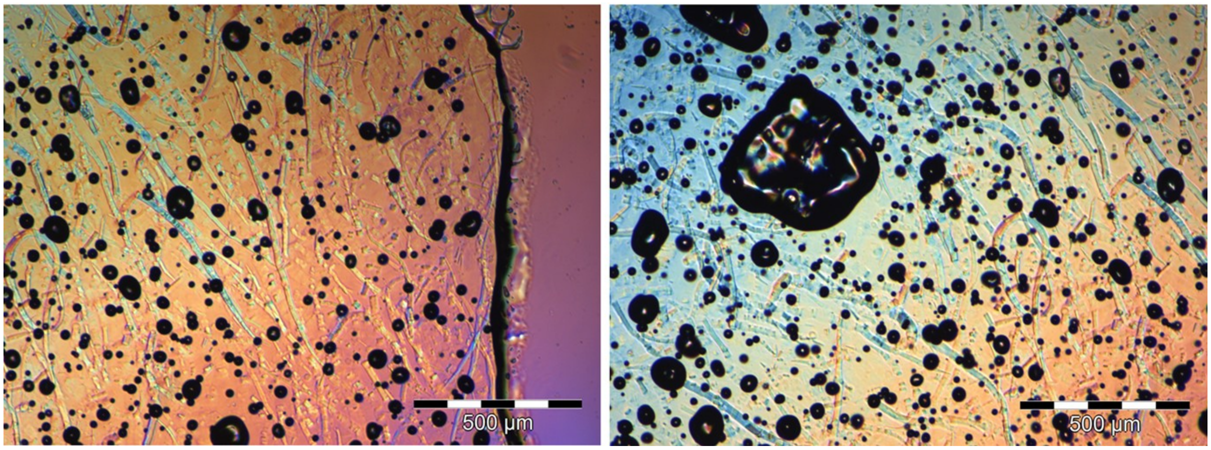

Figure S47: Enocell fibres in [mTBOH][OAc], some fibres are still visible, black spots are air bubbles

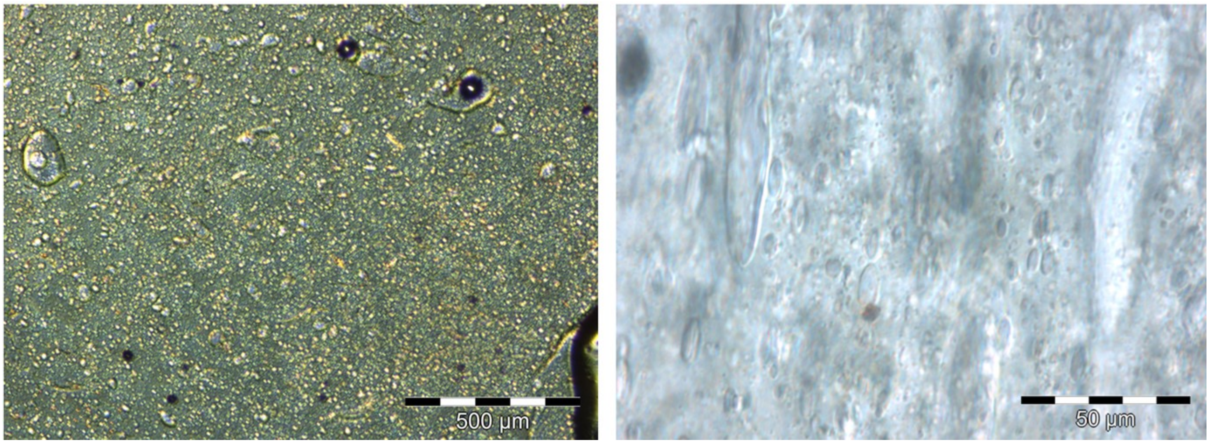

Figure S48: Enocell fibres in [dm3-mTBDH][OAc], no fibres are visible, round spots are air bubbles

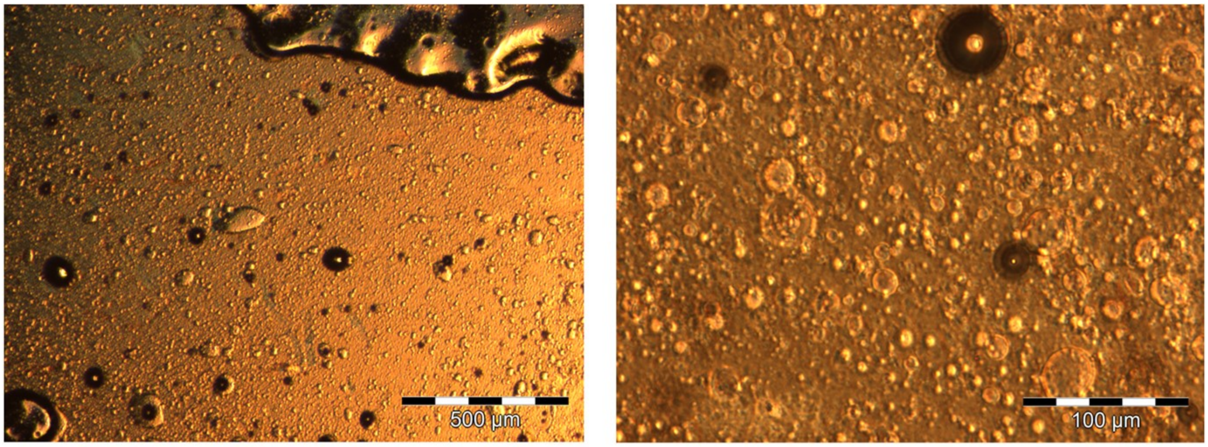

Figure S49: Enocell fibres in [m2-mTBDH][OAc], no fibres are visible, round spots are air bubbles

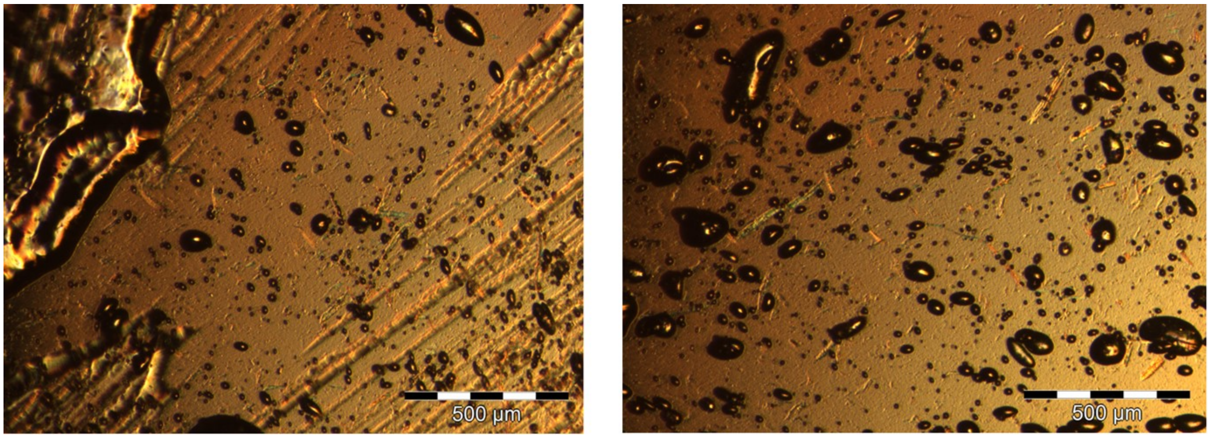

Figure S50: Enocell fibres in [dm3-m9 -mTBDH][OAc], few fibres are visible due to the non-optimal stirring, round spots are air bubbles

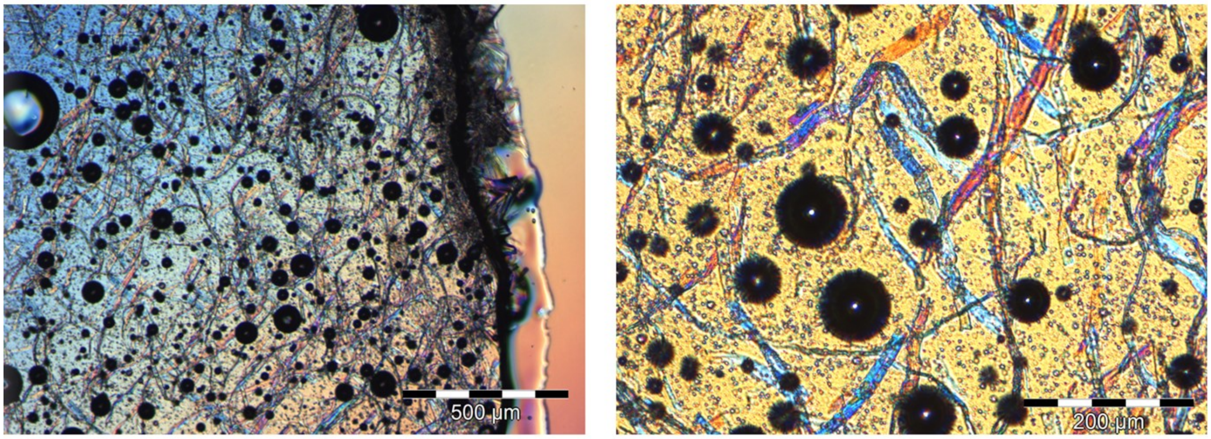

Figure S51: Enocell fibres in [dm39 -mTBDH][OAc] without DMSO, fibres are visible, round spots are air bubbles

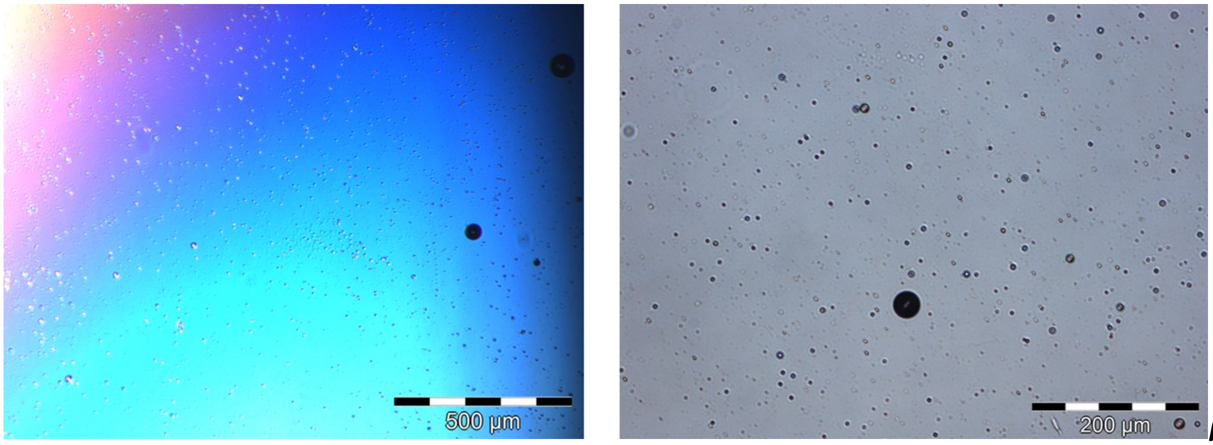

Fig

ure S52: Enocell fibres in [dm39 -mTBDH][OAc] with 10wt% DMSO, fibres are not visible, round spots are air bubbles

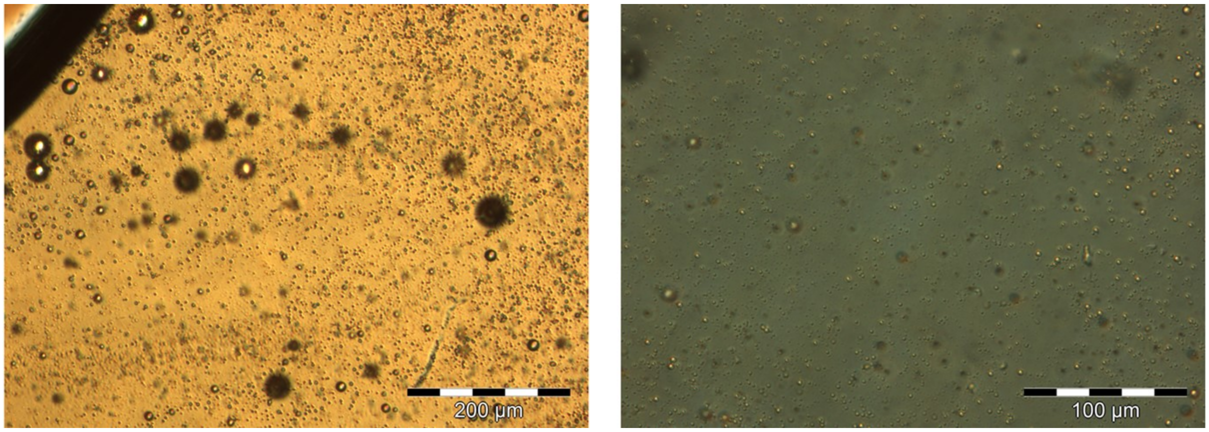

***Figure S53*: Enocell fibres in [mTBUH][OAc] fibres are not visible, round spots are air bubbles

VII. Prediction of hydrolysis free energies with quantum chemistry

a. Computational methods

The free energy of hydrolysis ($\Delta_h G$) of a series of mono-, di- and tetramethyl substituted derivatives of mTBD was calculated with quantum chemical methods (Scheme S1). The amidines DBN and DBU, and the guanidines mTBO, mTBN, mTBU and TBD (Scheme S2) were also included in the analysis for comparison.

Initial search for conformational isomers of each studied base and their hydrolysis products was done in Spartan'16[1] using the Merck Molecular Force Field (MMFF) method[2]. During the conformer search, chair flips and inversion operations were enforced to the rings and sp^3-hybridized nitrogen atoms respectively. Even though these operations may not connect rapidly-interconvertible conformations[3], they were necessary to make sure that the lowest energy structure would be obtained from the sampling. The keywords SEARCHMETHOD=SYSTEMATIC and KEEPALL were added to the options line to ensure that all the conformers found by the software were properly stored in the output. Each set of conformers produced this way was used as an input to COSMO*conf* v'21[4], using a custom job workflow based on the software's BP-TZVPD-FINE-COSMO+GAS_18 template. The job steps involving conformer search and reduction of the number of conformers were removed from the template, in an approach similar to that described by Kurtén et al.[5], and Hyttinen & Prisle[6]. Thus, the workflow used by COSMO*conf* was as follows: Conformer geometries were initially optimized at the BP86/def2-TZVP-COSMO[7–10] level of theory, with subsequent single-point calculation at the BP86/def2-TZVPD-COSMO[11] level of theory with a smooth-radii based isosurface cavity (FINE) parameterization, using TURBOMOLE v7.7[12,13]. At this point, duplicate conformers were identified and removed based on geometry similarity, using COSMO*conf*'s CLUSTER_GEOCHECK algorithm. Each unique conformer was then re-optimized at the same level of theory as described above, but this time without COSMO, yielding gas-phase BP86/def2-TZVPD//BP86/def2-TZVP geometries and electronic energies. Next, the gas-phase geometries were mapped to the COSMO-optimized geometries to ensure that they correspond to the same conformation (within a geometry

similarity threshold), using the MAP_GAS_COSMO algorithm. In the case that a COSMO conformer does not have a corresponding gas-phase conformer mapped to it, COSMO*conf* approximates the gas-phase energy with a BP86/def2-TZVPD single-point calculation done at the COSMO geometry.

Once the BP86/def2-TZVPD//BP86/def2-TZVP COSMO screening surface charge densities and gas-phase energies were obtained for each conformer set, the solvation free energy $(\Delta_{solv}G)$ of each species was obtained with the COnductor like Screening MOdel for Real Solvents (COSMO-RS)[14–16] implemented in COSMO*therm* v'21[17]. The solvent used was a SBIL:H_2O mixture with 1:1 ratio. The SBIL components (BH^+ and AcO^-) were modelled as two individual ions, providing two separate compound inputs with equal concentrations to COSMO*therm* while specifying the solvent mixture, as is recommended in the software's manual. The reference state used for calculating $\Delta_{solv}G$ was 1 bar of ideal gas and 1 mol/L for the liquid solvent. The value of $\Delta_{solv}G$ obtained for each species has a Boltzmann-weighted contribution from all of its conformations. Higher-level gas-phase energies and thermal free energies were also calculated for each species, but using only the conformation with the highest Boltzmann weight in the mixture, as predicted with COSMO-RS. To that end, the COSMO geometry was re-optimized at the ωB97XD/aug-cc-pVTZ level of theory[18,19] using the Gaussian v.16 software[20]. Frequency calculations served to ensure that each geometry sits on a minimum in the potential energy surface (PES), and provided thermal enthalpy and free energy corrections at 298.15 K and 1 bar, under the harmonic oscillator-rigid rotor approximation. Solution-phase free energies of hydrolysis $\Delta_h G_{soln}$ were calculated by combining the gas-phase free energy of hydrolysis $\Delta_h G_{gas}$ with the change in solvation free energies $\Delta\Delta_{solv}G$, as follows[21]:

$$BH^+{}_{(gas)} + H_2O_{(gas)} + AcO^-{}_{(gas)} \rightarrow Prod_{(gas)} + AcOH_{(gas)} :\ \Delta_h G_{gas} = \Delta_h E_{gas} + \Delta G_{therm}$$

$$BH^+{}_{(gas)} + H_2O_{(gas)} + AcO^-{}_{(gas)} \rightarrow BH^+{}_{(soln)} + H_2O_{(soln)} + AcO^-{}_{(soln)} :$$

$$\Delta_{solv}G_{BH^+} + \Delta_{solv}G_{H_2O} + \Delta_{solv}G_{AcO^-}$$

$$Prod_{(gas)} + AcOH_{(gas)} \rightarrow Prod_{(soln)} + AcOH_{(soln)} : \Delta_{solv}G_{Prod} + \Delta_{solv}G_{AcOH}$$

$$\Delta(\Delta_{solv}G) = (\Delta_{solv}G_{Prod} + \Delta_{solv}G_{AcOH}) - \left(\Delta_{solv}G_{BH^+} + \Delta_{solv}G_{H_2O} + \Delta_{solv}G_{AcO^-}\right)$$

$$BH^+{}_{(soln)} + H_2O_{(soln)} + AcO^-{}_{(soln)} \rightarrow Prod_{(soln)} + AcOH_{(soln)} :\ \Delta_h G_{soln}$$

$$\Delta_h G_{soln}{}^{\circ} = \Delta_h E_{gas} + \Delta G_{therm} + \Delta(\Delta_{solv}G)$$

Where $\Delta_h E_{gas}$ is the change in electronic energies and ΔG_{therm} is the change in thermal free energies.

Proton affinities, defined as the negative of the enthalpy of association between the free base and a proton in the gas-phase ($B + H^+ \rightarrow BH^+ : \Delta H_{pr}$), at 298.15 K, were obtained as follows:

$$PA = -\Delta H_{pr} = -\left(H_{BH^+} - \left(H_B + {}^5\!/_2 RT\right)\right)$$

Where $(5/2)\ RT$ is the gas-phase enthalpy of H^+. R is the ideal gas constant, and T is temperature.

b. Investigated systems

Scheme S1: mTBD and its hydrolysis products, N-aminopropyl-N'-methylpropyleneurea (APP) and N-methylaminopropyl-propyleneurea (MAPP), in their neutral and protonated forms. Description of the labels given to the studied mTBD derivatives as well as the numbering scheme for substitution positions are shown.

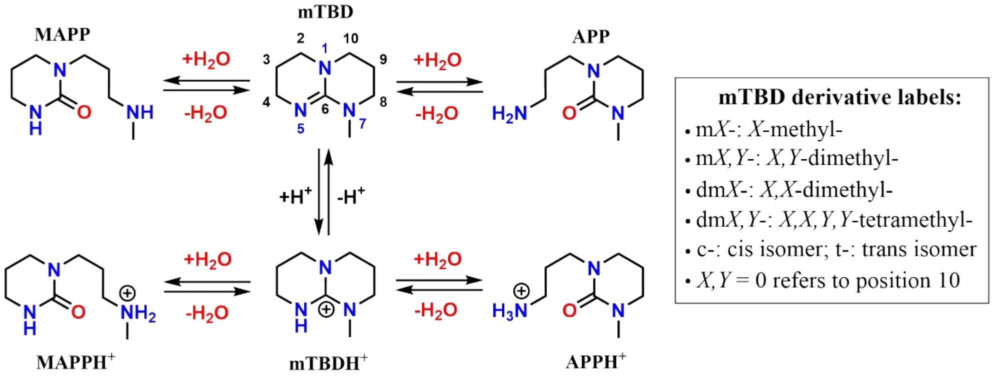

Scheme S2: Hydrolysis products of other studied guanidine and amidine derived superbases. Protonated forms omitted.

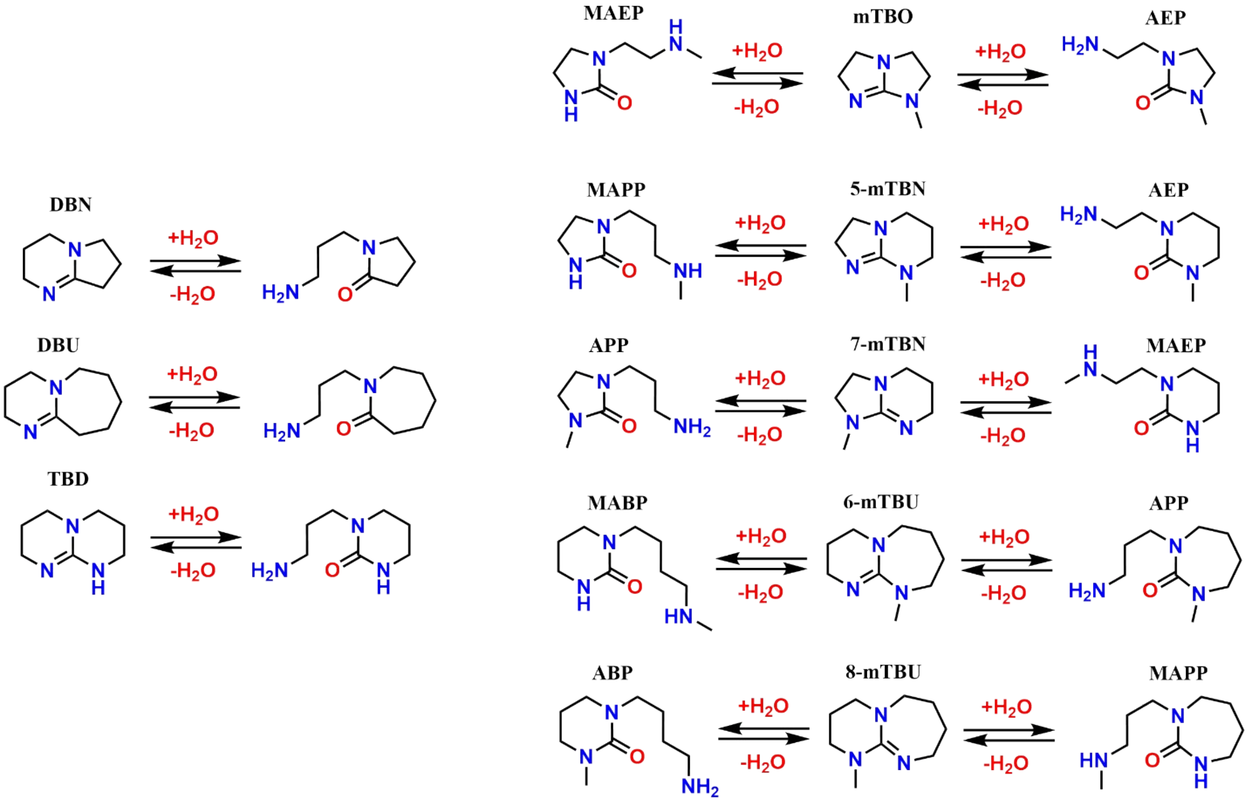

c. Free energies of hydrolysis

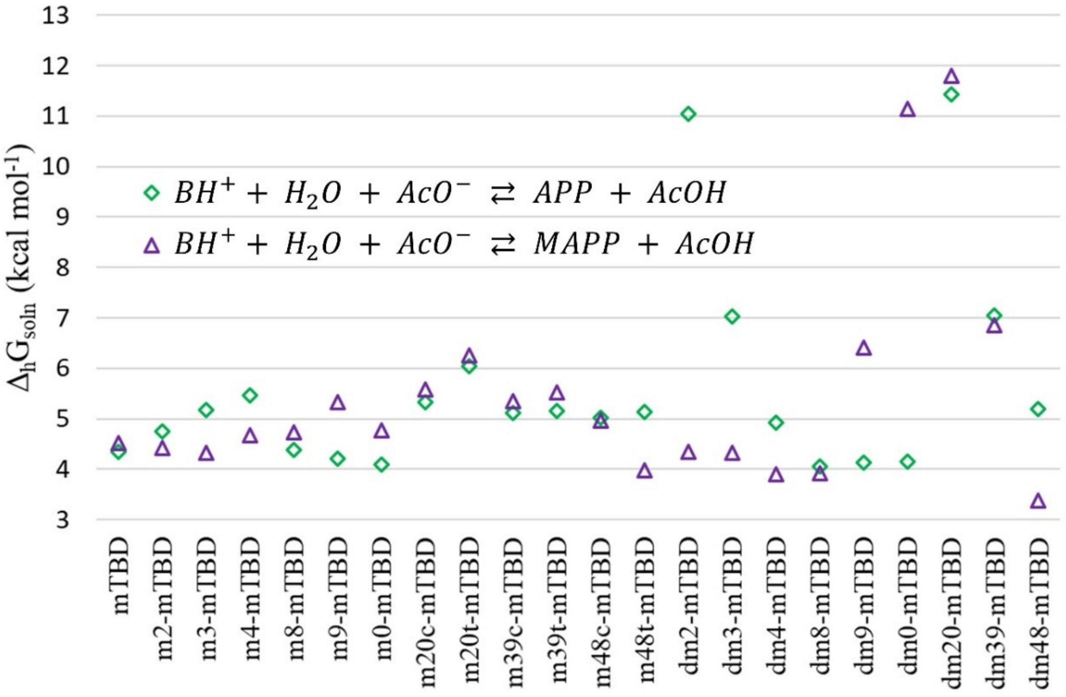

Figure S54: Solution-phase free energy of hydrolysis ($\Delta_h G_{soln}$) of mTBD and its studied derivatives in their neutral form. Electronic and thermal free energies calculated at the ωB97XD/aug-cc-pVTZ level of theory, at 298.15 K and 1 bar, under the harmonic oscillator-rigid rotor approximation. Solvation free energies calculated with COSMO-RS, at the BP-TZVPD-FINE level of theory, with a SBIL:H_2O 1:1 solvent mixture composition. Labelling of derivatives is described in Scheme S1.

Table S3*:* Proton affinities and gas-phase free energy of hydrolysis of other studied amidine (DBN and DBU) and guanidine (TBD, mTBO, mTBN and mTBU) derived superbases. Electronic and thermal free energies calculated at the ωB97XD/aug-cc-pVTZ level of theory, at 298.15 K and 1 atm, under the harmonic oscillator-rigid rotor approximation. Isomers of mTBN and mTBU as well as hydrolysis products are shown in Scheme S2.

**Base (B)**	**Proton affinity (kcal mol-1)**	$\Delta_h G_{soln}$ **(kcal mol-1) at 298.15 K and 1 bar, in a IL:H_2O (1:1) mixture** $BH^+ + H_2O + AcO^- \rightleftarrows Prod + AcOH$	
		$Prod = APP$	$Prod = MAPP$
mTBD	259.04	+4.34	+4.52
m2-mTBD	260.15	+4.76	+4.42
m3-mTBD	259.57	+5.18	+4.32
m4-mTBD	260.15	+5.46	+4.66
m8-mTBD	260.57	+4.38	+4.73
m9-mTBD	259.64	+4.22	+5.32
m0-mTBD	260.50	+4.09	+4.77
m20c-mTBD	261.24	+5.34	+5.58
m20t-mTBD	261.96	+6.04	+6.26
m39c-mTBD	259.90	+5.11	+5.34

m39t-mTBD	260.95	+5.16	+5.52
m48c-mTBD	261.95	+5.02	+4.96
m48t-mTBD	261.27	+5.14	+3.98
dm2-mTBD	261.11	+11.05	+4.35
dm3-mTBD	259.74	+7.02	+4.33
dm4-mTBD	260.79	+4.92	+3.90
dm8-mTBD	261.72	+4.06	+3.92
dm9-mTBD	259.88	+4.14	+6.41
dm0-mTBD	261.69	+4.15	+11.15
dm20-mTBD	261.79	+11.44	+11.80
dm39-mTBD	260.48	+7.04	+6.85
dm48-mTBD	263.81	+5.19	+3.39

***Table S4**:* Proton affinities and solution-phase free energy of hydrolysis of other studied amidine (DBN and DBU) and guanidine (TBD, mTBO, mTBN and mTBU) derived superbases. Gas-phase electronic and thermal free energies calculated at the ωB97XD/aug-cc-pVTZ level of theory, at 298.15 K and 1 bar, under the harmonic oscillator-rigid rotor approximation. Solvation free energies calculated with COSMO-RS, at the BP-TZVPD-FINE level of theory, with a SBIL:H_2O 1:1 solvent mixture composition. Isomers of mTBN and mTBU as well as hydrolysis products are shown in Scheme S2.

Base (B)	Proton affinity (kcal mol^{-1})	Hydrolysis product (Prod)	$\Delta_h G_{soln}$ (kcal mol^{-1}) at 298.15 K and 1 bar, in a IL:H_2O (1:1) mixture $BH^+ + H_2O + AcO^- \rightleftarrows Prod + AcOH$
DBN	257.64	APP	+7.69
DBU	253.58	APP	+9.01
TBD	256.21	APP	+7.39
mTBD	259.04	APP	+4.34
		MAPP	+4.52
mTBO	250.49	AEP	-4.37
		MAEP	-1.40
mTBN5	254.46	AEP	+3.74
		MAPP	+3.54
mTBN7	254.46	APP	+2.76
		MAEP	+5.48
mTBU6	257.52	APP	+4.28
		MABP	-1.94
mTBU8	260.50	ABP	-2.17
		MAPP	+3.97

Proton affinities, defined as the negative of the enthalpy of association between the free base and a proton in the gas-phase ($B + H^+ \rightarrow BH^+ : \Delta H_{pr}$), at 298.15 K, were obtained as follows:

$$PA = -\Delta H_{pr} = -\left(H_{BH^+} - \left(H_B + {}^5\!/_2 RT\right)\right)$$

Where $(5/2)\ RT$ is the gas-phase enthalpy of H^+. R is the ideal gas constant, and T is temperature.

Gas-phase basicity (GB) is the Gibbs free energy analogue of the proton affinity, calculated as:

$GB = -\Delta G_{pr} = -\left(G_{BH^+} - \left(G_B + G_{H^+}\right)\right)$

Table S5: Gas-phase basicity of the different superbases.

**Base (B)**	**Gas-phase basicity (kcal mol^{-1})**
mTBD	252.09
m2-mTBD	252.98
m3-mTBD	252.61
m4-mTBD	253.09
m8-mTBD	253.33
m9-mTBD	252.70
m0-mTBD	253.11
m20c-mTBD	253.48
m20t-mTBD	254.18
m39c-mTBD	253.05
m39t-mTBD	253.50
m48c-mTBD	254.35
m48t-mTBD	254.10
dm2-mTBD	253.84
dm3-mTBD	252.05
dm4-mTBD	251.30
dm8-mTBD	257.55
dm9-mTBD	251.97
dm0-mTBD	254.84
dm20-mTBD	254.20
dm39-mTBD	253.54
dm48-mTBD	256.38
DBN	245.89
DBU	248.31
TBD	250.70
mTBO	243.51
mTBN5	247.94
mTBN7	247.60
mTBU6	249.85
mTBU8	252.96

d. Interpretation of computational results

The results show that the proton affinity of mTBD is slightly increased upon methyl-substitution for all derivatives considered, indicating that the cellulose dissolving effectiveness would be unaffected, as far as their deprotonation potential is concerned. mTBN5, mTBN7 and mTBU6 have shown lower proton affinities than mTBD, but still within the range of values observed to be necessary for cellulose dissolution[22]. Comparison of the obtained hydrolysis free energies revealed some structures with enhanced stability: m20t and dm20 derivatives of mTBD and mTBN7. Among the mTBD derivatives, dm20

showed the largest increase in $\Delta_h G$, however the tetramethyl substitution could prove to be detrimental for the solubility of cellulose in an IL, since the base has a more hydrophobic surface.

It is clear that positions 2 and 10 (Scheme S1) are critical when the target of methyl substitution is to increase the hydrolytic stability of mTBD, but the underlying reasons are still not so clear. A possible explanation is the destabilization of the hydrolysis products relative to the base brought about by steric repulsion between methyl groups on the broken carbon chain and the carbonyl group on the intact ring. This interaction is maximal when the substitution occurs at the carbon adjacent to the cycle. Evidence for this effect is found by comparing the relative favorability of the two different pathways (APP and MAPP) for disubstituted derivatives dm2 and dm0. Substitution at position 2 has a larger effect on the $\Delta_h G$ of the APP pathway, while substitution at position 10 has a larger effect on the $\Delta_h G$ of the MAPP pathway. Derivative isomers m20c and m20t, which are monosubstituted at both 2 and 10 positions, are also granted enhanced stability towards hydrolysis via both the APP and MAPP pathways. However, this effect is partly offset by steric repulsion between the two methyl groups in the cis isomer derivative (m20c), which destabilizes the base relative to the hydrolysis products.

e. Comparison to hydrolysis experiments

Hydrolysis free-energy ($\Delta_h G$) values were estimated from experimental conversion percentages ($conv\%$), assuming the reaction was at equilibrium, as follows:

$$\frac{conv\%}{100} = \frac{[Prod]_{eq}}{[BH^+]_{eq}} = \frac{[Prod]_{eq}}{[BH^+]_0 - [Prod]_{eq}}$$

$$[Prod]_{eq} = \frac{[BH^+]_0(conv\%/100)}{1 + (conv\%/100)}$$

$$[BH^+]_{eq} = [AcO^-]_{eq} = ([BH^+]_0 - [Prod]_{eq})$$

$$[H_2O]_{eq} = [H_2O]_0 - [Prod]_{eq}$$

$$[AcOH]_{eq} = [Prod]_{eq}$$

$$[total]_{eq} = [BH^+]_{eq} + [H_2O]_{eq} + [Prod]_{eq} + [AcO^-]_{eq} + [AcOH]_{eq}$$

$$K_{eq} = \frac{\left([Prod]_{eq}/[total]_{eq}\right) . \left([AcOH]_{eq}/[total]_{eq}\right)}{\left([BH^+]_{eq}/[total]_{eq}\right) . \left([H_2O]_{eq}/[total]_{eq}\right) . \left([AcO^-]_{eq}/[total]_{eq}\right)}$$

Where K_{eq} is the hydrolysis equilibrium constant. The hydrolysis free-energy may then be obtained as:

$$\Delta_h G = - RT\, ln\left(K_{eq}\right)$$

Table S6: Free-energies of hydrolysis of SB-ILs estimated from experimental conversion percentages, in a SB-IL:H_2O mixture (1:1), at 130°C. Computationally predicted free-energies at the same conditions shown for comparison.

$\Delta_h G_{soln}$ (kcal/mol) IL:H2O (1:1) at 130°C	From experimental K_{eq}		$BH^+ + H_2O + AcO^- \rightleftarrows Prod + AcOH$ Predicted $\Delta_h G_{soln}$	
**Base (B)**	$Prod = APP$	$Prod = MAPP$	$Prod = APP$	$Prod = MAPP$
mTBD (1)	+4.33	+3.31	+3.80	+4.14
mTBU6 (2)	+0.85		+3.64	-2.81
mTBU8 (2)			-3.11	+3.47
mTBN5 (3)	+3.04		+3.51	+3.24
mTBN7 (3)	+5.50		+2.06	+5.36
mTBO (4)	-0.22		-4.85	-1.56
dm39-mTBD (5)	---		+7.08	+6.64
dm3-mTBD (7)	+7.64		+6.72	+4.04
dm9-mTBD (7)	+5.09		+3.71	+6.11
m2-mTBD (8)	+5.71		+4.06	+4.05
m0-mTBD (8)	---		+3.54	+4.30

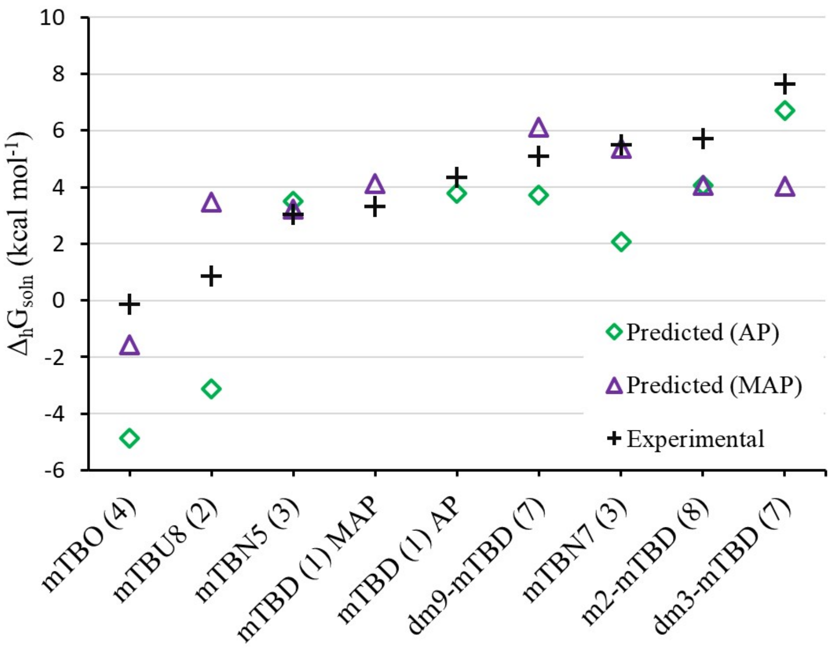

Figure S48: Free-energies of hydrolysis of SB-ILs estimated from experimental conversion percentages, in a SB-IL:H_2O mixture (1:1), at 130°C. Computationally predicted free-energies at the same conditions shown for comparison. Data ordered according to increasing experimental $\Delta_h G$ values.

References

(1) Spartan'16. *Wavefunct. Inc Irvine, CA* **2016**. https://doi.org/https://downloads.wavefun.com/Spartan20Manual.pdf.

(2) Halgren, T. A. MMFF VII. Characterization of MMFF94, MMFF94s, and Other Widely Available Force Fields for Conformational Energies and for Intermolecular- Interaction Energies and Geometries. **2000**, *20* (7), 730–748. https://doi.org/doi.org/10.1002/(SICI)1096-987X(199905)20:7<730::AID-JCC8>3.0.CO;2-T.

(3) Belostotskii, A. M.; Gottlieb, H. E.; Shokhen, M. Nitrogen Inversion in Cyclic Amines and the Bicyclic Effect. *J. Org. Chem.* **2002**, *67* (26), 9257–9266. https://doi.org/10.1021/jo020221i.

(4) BIOVIA COSMOconf. *Dassault Systèmes* **2021**, *http://www*.

(5) Kurtén, T.; Hyttinen, N.; Louise D'Ambro, E.; Thornton, J.; Prisle, N. L. Estimating the Saturation Vapor Pressures of Isoprene Oxidation Products C5H12O6 and C5H10O6 Using COSMO-RS. *Atmos. Chem. Phys.* **2018**, *18* (23), 17589–17600. https://doi.org/10.5194/acp-18-17589-2018.

(6) Hyttinen, N.; Prisle, N. L. Improving Solubility and Activity Estimates of Multifunctional Atmospheric Organics by Selecting Conformers in COSMO Therm. *J. Phys. Chem. A* **2020**, *124* (23), 4801–4812. https://doi.org/10.1021/acs.jpca.0c04285.

(7) Becke, A. . Density-Fnnctional Exchange-Energy Approximation with Correct Asymptotic Behavior. *J. Chem. Phys.* **1988**, *4* (4), 276–282. https://doi.org/10.1063/1.1749835.

(8) Perdew, J. P. Density-Functional Approximation for the Correlation Energy of the Inhomogeneous Electron Gas. *Pay Perform. Heal. Care Methods Approaches* **1986**, *33* (June), 8822–8824. https://doi.org/10.3768/rtipress.2011.bk.0002.1103.12.

(9) Weigend, F. Accurate Coulomb-Fitting Basis Sets for H to Rn. *Phys. Chem. Chem. Phys.* **2006**, *8* (9), 1057–1065. https://doi.org/10.1039/b515623h.

(10) Klamt, A.; Schuurmann, G. *COSMO: A New Approach to Dielectric Screening in Solvents with Explicit Expressions for the Screening Energy and Its Gradient*; 1993.

(11) Rappoport, D.; Furche, F. Property-Optimized Gaussian Basis Sets for Molecular Response Calculations. *J. Chem. Phys.* **2010**, *133* (13), 134105. https://doi.org/10.1063/1.3484283/920872.

(12) GmbH, T. TURBOMOLE V7.7 2022. a Development of University of Karlsruhe and Forschungszentrum Karlsruhe GmbH,. TURBOMOLE GmbH, since 2007. https://www.turbomole.org.

(13) Balasubramani, S. G.; Chen, G. P.; Coriani, S.; Diedenhofen, M.; Frank, M. S.; Franzke, Y. J.; Furche, F.; Grotjahn, R.; Harding, M. E.; Hättig, C.; Hellweg, A.; Helmich-Paris, B.; Holzer, C.; Huniar, U.; Kaupp, M.; Marefat Khah, A.; Karbalaei Khani, S.; Müller, T.; Mack, F.; Nguyen, B. D.; Parker, S. M.; Perlt, E.; Rappoport, D.; Reiter, K.; Roy, S.; Rückert, M.; Schmitz, G.; Sierka, M.; Tapavicza, E.; Tew, D. P.; Van Wüllen, C.; Voora, V. K.; Weigend, F.; Wodyński, A.; Yu, J. M. TURBOMOLE: Modular Program Suite for Ab Initio Quantum-Chemical and Condensed-Matter Simulations. *J. Chem. Phys.* **2020**, *152* (18), 184107. https://doi.org/10.1063/5.0004635.

(14) Klamt, A. Conductor-like Screening Model for Real Solvents: A New Approach to the Quantitative Calculation of Solvation Phenomena. *J. Phys. Chem.* **1995**, *99* (7), 2224–2235. https://doi.org/10.1021/j100007a062.

(15) Klamt, A.; Jonas, V.; Bürger, T.; Lohrenz, J. C. W. Refinement and Parametrization of COSMO-RS. *J. Phys. Chem. A* **1998**, *102* (26), 5074–5085. https://doi.org/10.1021/jp980017s.

(16) Eckert, F.; Klamt, A. Fast Solvent Screening via Quantum Chemistry: COSMO-RS Approach. *AIChE J.* **2002**, *48* (2), 369–385. https://doi.org/10.1002/AIC.690480220.

(17) BIOVIA COSMOtherm. Release 2019; Dassault Systèmes: San Diego 2019. http://www.3ds.com.

(18) Chai, J. Da; Head-Gordon, M. Long-Range Corrected Hybrid Density Functionals with Damped Atom-Atom Dispersion Corrections. *Phys. Chem. Chem. Phys.* **2008**, *10* (44), 6615–6620. https://doi.org/10.1039/b810189b.

(19) Kendall, R. A.; Dunning, T. H.; Harrison, R. J. Electron Affinities of the First-Row Atoms Revisited. Systematic Basis Sets and Wave Functions. *J. Chem. Phys.* **1992**, *96* (9), 6796–6806. https://doi.org/10.1063/1.462569.

(20) M. J. Frisch, G. W. Trucks, H. B. Schlegel, G. E. Scuseria, M. A. Robb, J. R. Cheeseman, G. Scalmani, V. Barone, G. A. Petersson, H. Nakatsuji, X. Li, M. Caricato, A. Marenich, J. Bloino, B. G. Janesko, R. Gomperts, B. Mennucci, H. P. Hratchian, J. V. Ort, and D. J. F. Gaussian v.09. Gaussian, Inc., Wallingford CT, 2016. 2009.

(21) Hellweg, A.; Eckert, F. Brick by Brick Computation of the Gibbs Free Energy of Reaction in Solution Using Quantum Chemistry and COSMO-RS. *AIChE J.* **2017**, *63* (9), 3944–3954. https://doi.org/10.1002/aic.15716.

(22) Parviainen, A.; King, A. W. T.; Mutikainen, I.; Hummel, M.; Selg, C.; Hauru, L. K. J.; Sixta, H.; Kilpeläinen, I. Predicting Cellulose Solvating Capabilities of Acid-Base Conjugate Ionic Liquids. *ChemSusChem* **2013**, *6* (11), 2161–2169. https://doi.org/10.1002/cssc.201300143.
